# A combination of the K-L and S-P approaches for treating acetabular posterior wall factures accompanied by femoral head fractures with open reduction and internal fixation

**DOI:** 10.1186/s12893-022-01597-w

**Published:** 2022-05-10

**Authors:** Shichao Lian, Zhong Yang, Zongliang Hu, Weidong Mu

**Affiliations:** 1 Department of Traumatic Orthopedics, Shandong Provincial Hospital, Shandong University, No. 324 Jingwu Weiqi Road, Jinan, 250012 Shandong China; 2 Zoucheng People’s Hospital, No. 59 Qianquan Road, Zoucheng, 273500 Shandong China; 3 Binzhou Medical University, No. 346 Guanhai Road, Yantai, 264003 Shandong China

**Keywords:** Pipkin type IV fracture, Acetabular posterior wall fracture, Femoral head fracture, Surgical approach, K-L approach combined with S-P approach

## Abstract

**Background:**

In clinical practice, acetabular posterior wall fracture combined with femoral head fracture is rare. However, with the increasing number of engineering and traffic accidents, such fractures, have increased significantly in recent years. This paper aims to explore the clinical efficiency of the Kocher-Langenbeck (K-L) and Smith-Petersen (S-P) combined approaches for open reduction and internal fixation (ORIF) of acetabular posterior wall fractures accompanied by femoral head fractures (Pipkin type IV fractures).

**Methods:**

A retrospective study was conducted on 8 patients who underwent open reduction and internal fixation (ORIF) of Pipkin type IV fractures through the K-L combined with S-P approach in our hospital from January 2015 to January 2020. All 8 patients were successfully operated on without serious complications, such as important blood vessel and nerve damage, with an operation time of 143.8 ± 44.38 min and intraoperative blood loss of 225 ± 70.71 ml. Perioperative data were recorded. The Harris score was used to evaluate the clinical effect. Fracture reduction quality was evaluated according to the Matta radiological standard. The grade of ectopic ossification was evaluated by the Brooker grading method, and the stage of femoral head necrosis was evaluated by Ficat-Arlet staging.

**Results:**

The Harris score increased significantly from 57.38 ± 4.779 at 3 months, to 76.13 ± 3.682 at 6 months, 88.25 ± 3.495 at 12 months, and 92.13 ± 2.232 at 36 months postoperatively. After statistical analysis, compared with the previous observation time point, the data comparison differences between the groups were statistically significant. P < 0.001, P < 0.001, P < 0.05). By the time of the latest follow-up, 6 of the 8 patients had recovered to the level of pre-injury sports capacity. In contrast, the other 2 patients remained below the level of pre-injury sports capacity. In terms of imaging evaluation, the quality of fracture reduction on radiographs was graded as excellent in 6 patients and good in 2 patients according to Matta’s criteria. At the last follow-up, no heterotopic ossification or femoral head necrosis was found in of all the images. In addition, the hip joint space was normal in 6 cases, mildly narrowed in 1 case, and clearly narrowed in 1 case.

**Conclusions:**

The K-L combined with S-P approach provides effective exposure for the reduction and fixation of Pipkin type IV fractures and achieves satisfactory clinical outcomes.

**Supplementary information:**

The online version contains supplementary material available at 10.1186/s12893-022-01597-w.

## Background

In clinical practice, acetabular posterior wall fracture combined with femoral head fracture is rare. Even the dislocation of the hip joint causing femoral head fracture accounts for only 5 − 15% [1]. However, in recent years, with the increasing number of engineering and traffic accidents, such fractures have increased significantly. They are caused mostly by high-energy injuries, such as falling injuries and traffic accident injuries [2]. For acetabular fracture with femoral head fracture, restoring the normal anatomical morphology of the hip joint and obtaining good clinical function while minimizing the occurrence of complications such as femoral head necrosis, malunion and traumatic arthritis of the hip joint is still a major problem for orthopedic doctors. In 1951, Thompson [3] proposed the classification of posterior dislocation of the hip joint and introduced femoral head fracture combined with posterior dislocation of the hip joint as an independent subtype (Thompson Epstein V type) for the first time. In 1957, Pipkin [1] further subdivided the type V fracture in the Thompson Epstein classification into four subtypes, so femoral head fracture combined with posterior dislocation of the hip joint is also called Pipkin fracture. In Pipkin’s classification, besides accompanied by posterior dislocation of the hip joint, type I is the femoral head fracture caudad to fovea capitis, type II is the femoral head fracture cephalad to fovea capitis, and type III is type I or II fracture with associated femoral neck fracture. Pipkin type IV is combined with acetabular fractures based on the anterior 3 types, and it’s also the main difference between it and other 3 types, usually acetabular posterior wall fractures. The typical injury mechanism of Pipkin type IV fracture is that in the state of hip and knee flexions, great violence is transmitted to the femoral head through the femur. The femoral head violently strikes the acetabulum, resulting in the fracture of the femoral head and acetabulum, usually combined with posterior dislocation of the hip joint [4]. The causes of Pipkin type IV fracture’s refractory is the complication as well as sequela more after injury and its operative difficulty. Compared with type I, II and III Pipkin fractures, Pipkin type IV fracture is faced with the problems of not only protecting the blood supply of femoral head, but also dealing with acetabular fracture and protecting hip joint capsule. Especially the fracture of acetabular posterior wall, is easy to cause the instability of hip joint. Therefore, in addition to protecting the blood supply of the femoral head and effectively reducing and fixing the fractures of femoral head and acetabular, many factors such as repairing the hip joint capsule and avoiding excessive peeling of the joint capsule should also be considered. Therefore, the difficulty of operation increases significantly, and a single incision is difficult to meet the above treatment requirements at the same time. At present, most orthopedic scholars have studied more types I, II, and III Pipkin fractures. However, there are few studies and reports on the rare type IV Pipkin fractures. This paper aims to retrospectively explore the clinical effect of K-L combined with the S-P approach in treating posterior acetabular wall combined with femoral head fracture (Pipkin type IV fracture).

## Methods

### Patients

There were 8 patients in this group, all had Pipkin IV fractures, and the fracture lines of the femoral head were located below the central fovea. All patients were male, aged from 24 to 53 years, with an average of 41.38 ± 11.35 years. There were 6 cases of falling injury and 2 traffic accident injury cases. Among them, 3 cases were complicated with sciatic nerve injury (Table 1).

This study was approved by the Shandong Provincial Hospital Medical Science Research Ethics Committee. Informed consent was obtained from all patients. All methods performed in this study were in accordance with the Declaration of Helsinki.

### Surgical technique

**Fig. 1:**
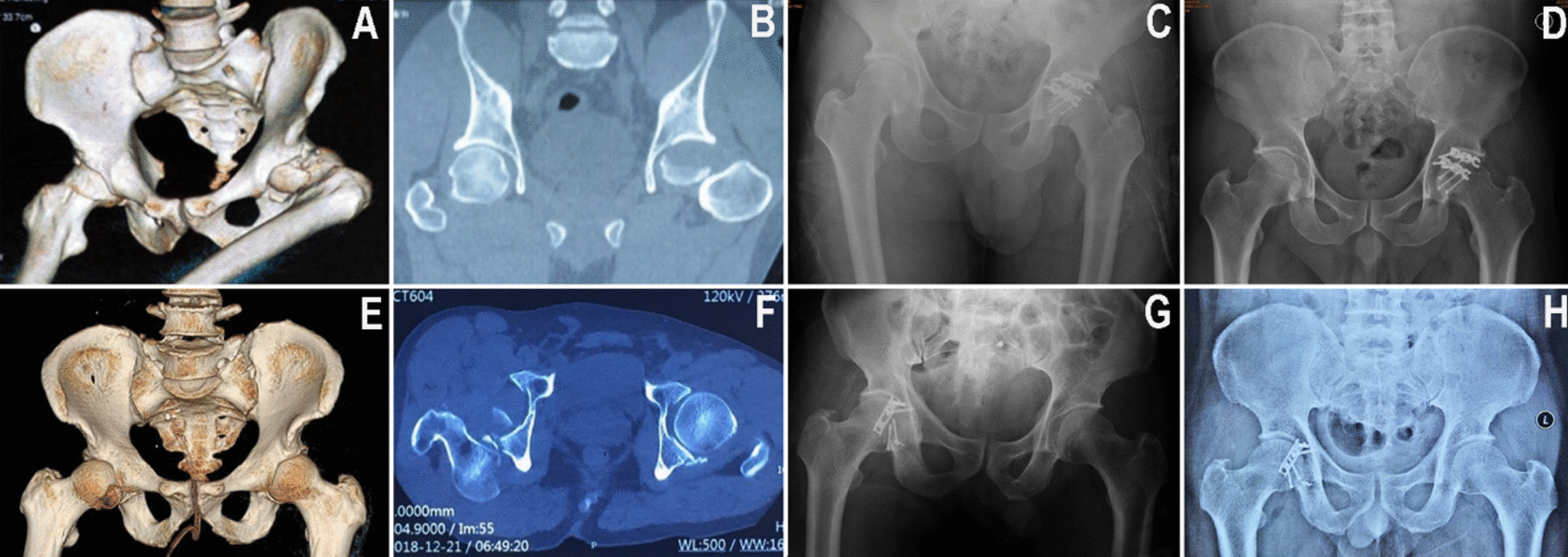
Typical case images. **A**, **B**, **E**, **F** Preoperative CT showed an acetabular posterior wall fracture and posterior dislocation of the hip. The fracture line of the left femoral head was located below the fovea. **C**, **G** X-ray examination immediately after the operation showed that the fracture was anatomically reduced, and the position of internal fixation was good. **D**, **H** X-ray examination at follow-up 1 year after operation showed that the fracture healed, no femoral head necrosis occurred, and the hip joint space was normal

Manual reduction of hip dislocation and bone traction of the tibial tubercle were performed in emergency situations. Improve relevant auxiliary examination and consultation of relevant departments before operation (Fig. 1). Surgical treatment was performed 4–7 days after injury, with an average of 4.875 ± 1.126 days.

**Fig. 2:**
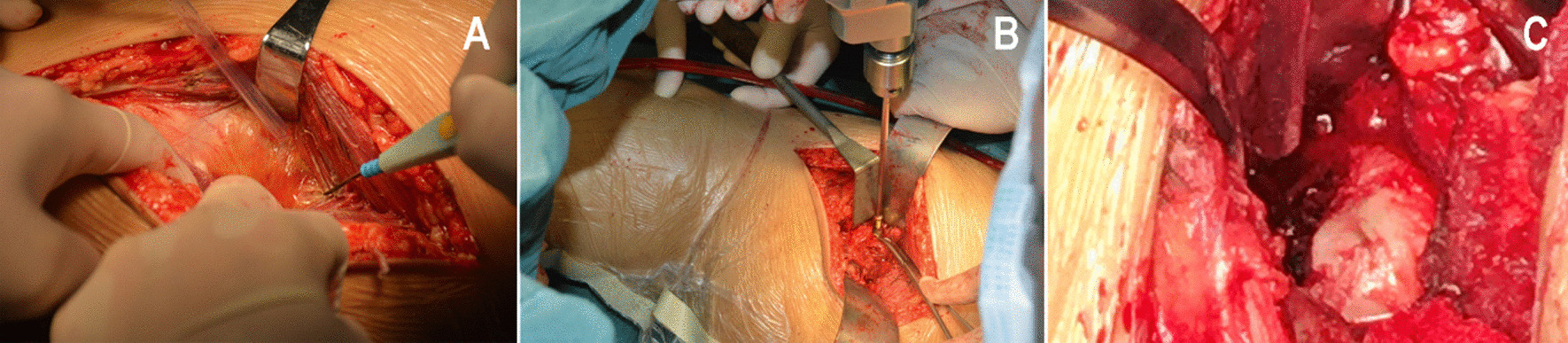
Main surgical steps of the K-L approach combined with the S-P approach for ORIF. **A** Posterior K-L approach. **B** Reduction and fixation of acetabular posterior wall fracture through the K-L approach. **C** Reduction and fixation of femoral head fracture through the S-P approach; *K-L* Kocher-Langenbeck approach, *S-P* Smith-Petersen approach, *ORIF* open reduction and internal fixation

The patient underwent general anesthesia with endotracheal intubation, took the floating position, and used the K-L approach behind the hip joint first. The apex of the greater trochanter was taken as the center, and an incision with a length of approximately 10 cm was made to expose the joint capsule (Fig. 2A). The posterior wall fracture of the acetabulum was displaced and partially comminuted. The residual joint capsule was protected, the posterior wall fracture block was reduced, the flatness of the joint surface was restored, and plate-screw was used to fix the fracture block. When the fracture block is too small, use suture anchor to fix it. Due to the various shapes of fracture blocks, there is no unified standard for the selection of internal fixation. The aim is to preserve the bone mass of the posterior acetabular wall as much as possible to maintain the stability of the hip joint (Fig. 2B). In patients with sciatic nerve injury, the sciatic nerve was released after hip joint reduction. Traction and external rotation of the affected limb revealed the fracture of the femoral head, showing that the free rotation and reduction of the fracture block of the femoral head were difficult. The procedures involve the following: taking the S-P approach in front of the hip joint (Fig. 2C), using a limited open distal part, entering in the gap between the fascia lata and the sartorius muscle, pulling off the straight femoral muscle, exposing and partially cutting the articular capsule, abducting and rotating the affected limb, exposing the femoral head fracture, reducing the fracture and fixing it with a countersunk nail. When the position of the fracture line of the femoral head in some patients is lower and it is difficult to place the implant, the towel forceps should be temporarily and percutaneously fixed under fluoroscopy. Postoperative routine prevention of infection, heterotopic ossification, and deep venous thrombosis. Bone traction was continued for 4 weeks, with a weight of 3–5 kg. Quadriceps femoris contraction and ankle extension and flexion exercises were performed on the 2nd day after the operation. Hip and knee extension and flexion exercises were performed on the 1st week, walking with partial weight by the aid of walking aids for 6–8 weeks and gradually walking with full weight after 3 months.

### Evaluating indicator

Perioperative data were recorded. The Harris [5] score was used to evaluate the clinical effect. The quality of fracture reduction was evaluated according to the Matta [6] radiological standard, the ectopic ossification grade was evaluated by the Brooker [7] grading method, and the stage of femoral head necrosis was evaluated by Ficat-Arlet [8] staging.

### Statistical analysis

SPSS 25.0 software was used for statistical analysis. The measurement data are expressed as xˉ ± s (mean ± SD). The data at more than three times points and the pairwise comparison between them were analyzed by one-way ANOVA. P < 0.05 indicates that the difference is statistically significant.

## Results

### Clinical outcomes and complication management

All patients completed the operation without serious complications, such as important blood vessels and nerve injury. The operation time was 143.8 ± 44.38 min. The average amount of intraoperative bleeding was 225 ± 70.71 ml. All incisions healed in Grade A without infection.

Up to the last follow-up, no complications, such as avascular necrosis of the femoral head, traumatic arthritis of the hip, fracture of internal fixation, loss of fracture reduction or fracture nonunion, occurred in all cases, and no patients needed reoperation.

**Fig. 3:**
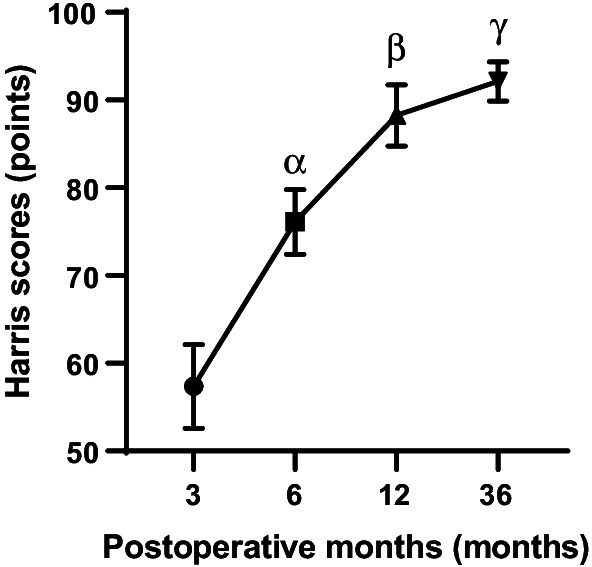
Pairwise comparison of Harris scores during 12 to 36 months of follow-up. P(α) < 0.001, 6 months versus 3 months, Harris scores improved significantly. P(β) < 0.001, 12 months versus 6 months, Harris scores improved significantly. P(γ) < 0.05, 36 months versus 12 months, the improvement in Harris scores was still statistically significant

All 8 patients were followed up for more than 36 months. Three patients who had sciatic nerve injury before the operation and showed a weakness of the ankle joint and toe dorsum were treated with mecobalamin and other nutritional nerve drugs. Their sciatic nerve injury symptoms gradually disappeared and returned to normal 6–12 weeks after the operation. All combined injuries were treated satisfactorily, and other fractures healed within 6 months after the operation. One patient had a common peroneal nerve injury before the operation, did not fully recover at the last follow-up, and had lower limb numbness and foot drooping. However, it did not affect the function of the hip joint, so it did not affect the hip functional evaluation. After the operation, nutritional nerve and rehabilitation treatment were continued for common peroneal nerve injury; the other 7 patients recovered well in walking and labor ability. The Harris score increased significantly from 57.38 ± 4.779 at 3 months to 76.13 ± 3.682 at 6 months (P < 0.001) and from 88.25 ± 3.495 at 12 months to 92.13 ± 2.232 at 36 months (P < 0.05). At the last follow-up, of the 8 patients, 6 recovered to the preinjury motor ability level, and 2 still did not recover to the preinjury motor ability level. (Table 2; Fig. 3)

### Image evaluation

According to the Matta evaluation standard, postoperative X-ray and CT showed that the quality of fracture reduction was excellent in 6 cases and good in 2 cases. According to the Brooker criteria, no ectopic ossification was found. At the last follow-up, 8 patients were rated as grade 0 according to Ficat-Arlet staging, and no femoral head necrosis was found. At the last follow-up, the hip joint space was normal in 6 cases, mild stenosis in 1 case and evidentstenosis in 1 case (Table 3). Typical case images are shown in Fig. 1.

## Discussion

The Pipkin classification is a commonly used classification system for femoral head fractures. Pipkin IV refers to the anterior 3 types combined with acetabular fractures. Asghar et al. [9] believe that this type of fracture needs surgery if it meets any of the following conditions: displacement of articular fracture surface > 1 mm, instability after joint reduction, and fracture range of acetabular wall > 20%; Wang et al. [4] indicated that it is very important to restore the consistency and stability of the hip joint and remove small and comminuted intra-articular fractures as soon as possible. In addition, if the articular surface fracture of the femoral head cartilage is involved in the weight-bearing area, even small fracture blocks should be fixed and repaired. However, if the cartilage surface is defective or cannot be fixed, femoral head cartilage transplantation can reduce complications and obtain good results [10]. Ahmed et al. [11] A delayed reduction was associated with a higher rate of femoral head necrosis. Crock et al. [12] noted that avascular necrosis of the femoral head is an important factor affecting the prognosis of femoral head fracture, which is closely related to the injury of blood vessels supplying the femoral head, especially the medial circumflex femoral artery. Therefore, surgery is recommended as soon as possible. Some scholars reported that the operation should be performed within 3 days after injury. The author’s clinical data showed that early traction closed reduction of the hip joint and operation within 7 days after the injury did not increase the incidence of femoral head necrosis.

In this study, a limited S-P incision combined with a posterior K-L incision was used to treat Pipkin type IV fractures. The procedures are as follows: take the posterior K-L approach first, expose but not free the sciatic nerve to protect the sciatic nerve to the greatest extent, fix the fracture of the posterior wall of the acetabulum, protect (or repair) the joint capsule attached to the posterior wall of the acetabulum, determine the fracture position of the femoral head by rotating the lower limbs, and then take the distal part of the anterior S-P approach to fully expose and fix the fracture. Studies have shown that the distal part of the S-P approach is sufficient for the exposure and repair of femoral head fractures [13].

Key points and significance of operation: (1) Because only the distal part of the S-P approach is selected, the stable structure in front of the hip joint can be protected to the greatest extent, and the stripping of muscle tissue can be reduced to lower the risk of ectopic ossification; (2) When the femoral head fracture block is reduced and fixed through partial S-P incision, the “4” position of hip flexion and abduction can be maintained to expose the anterior and inferior fracture block when the joint capsule and synovial tissue connected to it are protected to the greatest extent; (3) The partial S-P incision for femoral head fracture fixation keeps away from the branch of femoral head blood supply mainly by the posterior medial femoral circumflex artery (MFCA). Thus, the purpose of reducing the risk of femoral head necrosis may be achieved. (4) The exposure of the anterior approach is clear, which is conducive to the reconstruction of the femoral head with bone graft and the matching and fixation of the head and acetabulum. (5) Studies have shown that the sub-branch of the MFCA penetrates the bone cartilage junction of the femoral head along the posterior upper part of the femoral neck to nourish the femoral head [14]. In the operation of the posterior approach, we tried to reduce the separation of the short external rotator muscle group, especially protecting the muscle group below the level of the external obturator muscle and the internal obturator muscle. Pay attention to the M-shaped incision of the joint capsule and avoid disturbing the synovial branch of the MFCA when resetting the posterior wall of the acetabulum. At the same time, without posterior reduction of the femoral head fracture, it also reduces the possibility of interfering with the MFCA and protects the blood supply of the femoral head.

At present, the commonly used surgical approaches for the treatment of Pipkin type IV fractures mainly include the simple Kocher-Langenbeck (K-L) and surgical dislocation of the hip joint (Ganz) approaches. The simple K-L approach has a wide exposure range and can address fractures of the posterior acetabulum and femoral head simultaneously, which is favored by many scholars [15]. However, its surgical trauma is large, the incidence of complications such as vascular and nerve injury, avascular necrosis of the femoral head, and heterotopic ossification is high. It is difficult to reduce and fix under direct vision for anterior and inferior femoral head fracture blocks. Ganz et al. [16] proposed the improved K-L approach in 2001, also known as the surgical dislocation of hip joint (Ganz) approach. This approach can protect the blood supply of the femoral head fully expose the fracture site and achieve complete reduction and fixation. It has obtained good curative effects treating Pipkin type IV fractures [17, 18]. However, the operation of this approach is complex, the learning curve is long, and the surgical trauma is large. The incidence of postoperative femoral head necrosis is 12.5% [15], and the incidence of ectopic ossification is as high as 20–60% [19–21]. At the same time, because this approach requires femoral greater trochanter osteotomy, there is a risk of nonunion of the osteotomy block.

There are relatively few cases of Pipkin IV fracture treated by the combined anterior and posterior approach. Ellis et al. [22] have made relevant reports and agreed with its curative effect. Some scholars have conducted a 15-year follow-up study on a patient with a Pipkin IV fracture treated by a combined anterior and posterior approach and found that it can be used as an appropriate alternative method to treat Pipkin IV fractures under appropriate circumstances [23]. Although it has been reported that the modified Gibson approach has the advantage of treating two parts of fractures with one incision simultaneously [24], two approaches from one incision have the disadvantage of mutual interference due to their close distance and may aggravate the damage to soft tissue. In this study, the exposition of fractures through two incisions increased some trauma, but it did not increase the traction injury of soft tissue, and the exposition was clearer. Our latest research also shows that the clinical effect of a direct anterior combined with direct posterior approach, which represents the idea of a combined approach, is also satisfactory.

Shortcomings of this study: To date, none of the patients in this group have serious surgical complications. However, the follow-up time of this study was 3 ~ 36 months, which is much shorter than the 81 months of Oransky et al. [25]. Therefore, there is still the possibility of complications such as avascular necrosis of the femoral head and traumatic arthritis of the hip in the later stage of this group, which needs further follow-up observation. Meanwhile, the number of Pipkin type IV fracture with femoral neck fracture cases is very small, and these cases was not included in this study. Brumback [26] proposed an improved classification system in 1987 and classified this kind of fracture as Brumback 3B fracture. Such cases maybe have a worse prognosis, and the treatment methods would be changed accordingly. Next, We will collect these cases separately for study, summarize appropriate treatment methods and reduce complications.

## Conclusions

Our retrospective study showed that the K-L approach combined with the limited S-P approach clearly expose the fracture site, conducive to anatomical reduction and strong fixation, and the clinical effect is satisfactory.

**Table 1:** Demographic data of eight patients with Pipkin type IV fractures undergoing the K-L approach combined with the S-P approach for ORIF

Category (n = 8)	Mean ± SD/ n(%)
Age	41.38 ± 11.35
Preoperative time (day)	4.875 ± 1.126
Intraoperative bleeding (ml)	225.0 ± 70.71
Operation time (min)	143.8 ± 44.38
Intraoperative bleeding	
Red blood cell (U)	2.75 ± 1.035
Plasma (ml)	200.0 ± 0.00
Sex	
Male	8 (100.0%)
Female	0 (0.0%)
Cause of injury	
Traffic accident injury	2 (25.0%)
Falling injury	6 (75.0%)
Combined with sciatic nerve injury	
Yes	3 (37.5%)
No	5 (62.5%)

*K-L* Kocher-Langenbeck approach, *S-P* Smith-Petersen approach, *ORIF* open reduction and internal fixation

**Table 2:** Harris scores of eight patients postoperatively

Category (n = 8)	Mean ± SD
3 months	57.38 ± 4.779
6 months	76.13 ± 3.682
12 months	88.25 ± 3.495
36 months	92.13 ± 2.232

**Table 3:** Imaging evaluation of eight patients up to the last follow-up

Category (n = 8)	n (%)
Matta standard	
Excellent (A)	6 (75.0%)
Good (B)	2 (25.0%)
Joint space stenosis	
Normal	6 (75.0%)
Mild stenosis	1 (12.5.0%)
Obvious stenosis	1 (12.5.0%)
Brooker criteria	
Heterotopic ossification	0
Without heterotopic ossification	8 (100.0%)
Ficat–Arlet staging	
Femoral head necrosis	0
Without necrosis of femoral head	8 (100.0%)

## Supplementary information

**Additional file 1.** Imaging data.

**Additional file 2.** Statistical data.

**Additional file 3: ** Table data.

## Data Availability

All data generated or analysed during this study are included in this published article [and its Additional files 1, 2 and 3].

## Abbreviations

K-L: Kocher-Langenbeck approach
S-P: Smith-Petersen approach
ORIF: open reduction and internal fixation
MFCA: medial femoral circumflex artery
