# Supplementary material for: A combination of the K-L and S-P approaches for treating acetabular posterior wall factures accompanied by femoral head fractures with open reduction and internal fixation
Source: BMC Surg. 2022 May 10;22:165. doi: 10.1186/s12893-022-01597-w (PMC9092786; doi:10.1186/s12893-022-01597-w)
Supplement: Supplementary file 1 — Additional file 1. Imaging data. [file 12893_2022_1597_MOESM1_ESM.pdf]

This document is a summary of imaging data of 8 patients.

Follow-up Results

- All 8 patients were followed up for more than 36 months.
- 3 patients were complicated with sciatic nerve injury before operation.
- Up to the last follow-up, all 8 patients had good fracture healing and no internal fixation loosening.
- Functionally, by the time of latest follow-up, 6 of the 8 patients had recovered to the level of pre-injury sports capacity, whereas the other 2 patients remained under the level of pre-injury sports capacity.
- To the latest follow-up, no heterotopic ossification and femoral head necrosis on images were found in anyone of them, in addition, the hip joint space was normal in 6 cases, mildly narrowed in 1 cases, and obviously narrowed in 1 case.
- According to Matta's criteria, in terms of imaging evaluation, quality of fracture reduction on radiographs was graded as excellent in 6 patients and good in 2 patient.
- Harris score results are reflected in data statistical analysis.

CASE 1

- -LIU, MALE, 42Y, Traffic Accident Injury, LEFT.

Preoperative

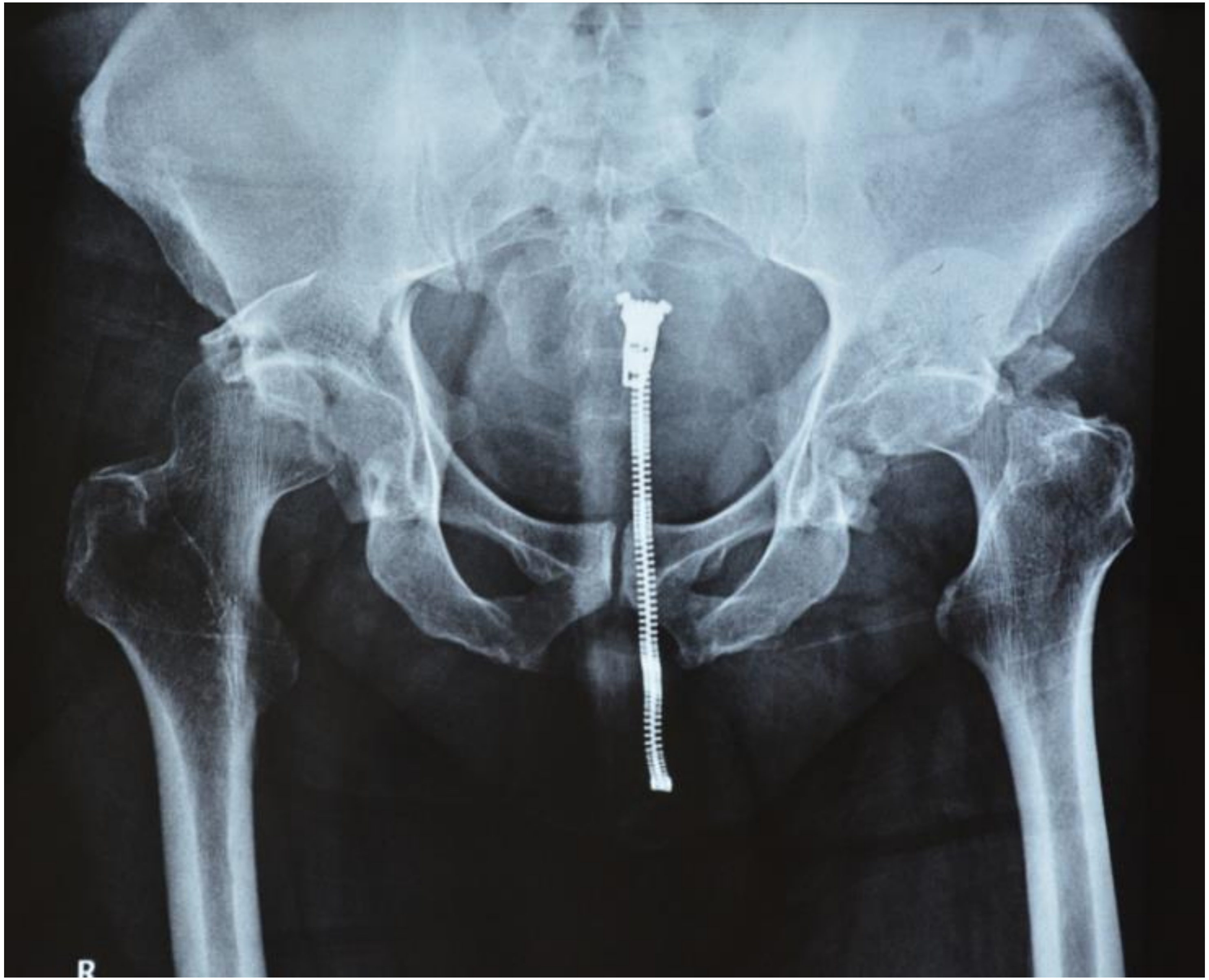

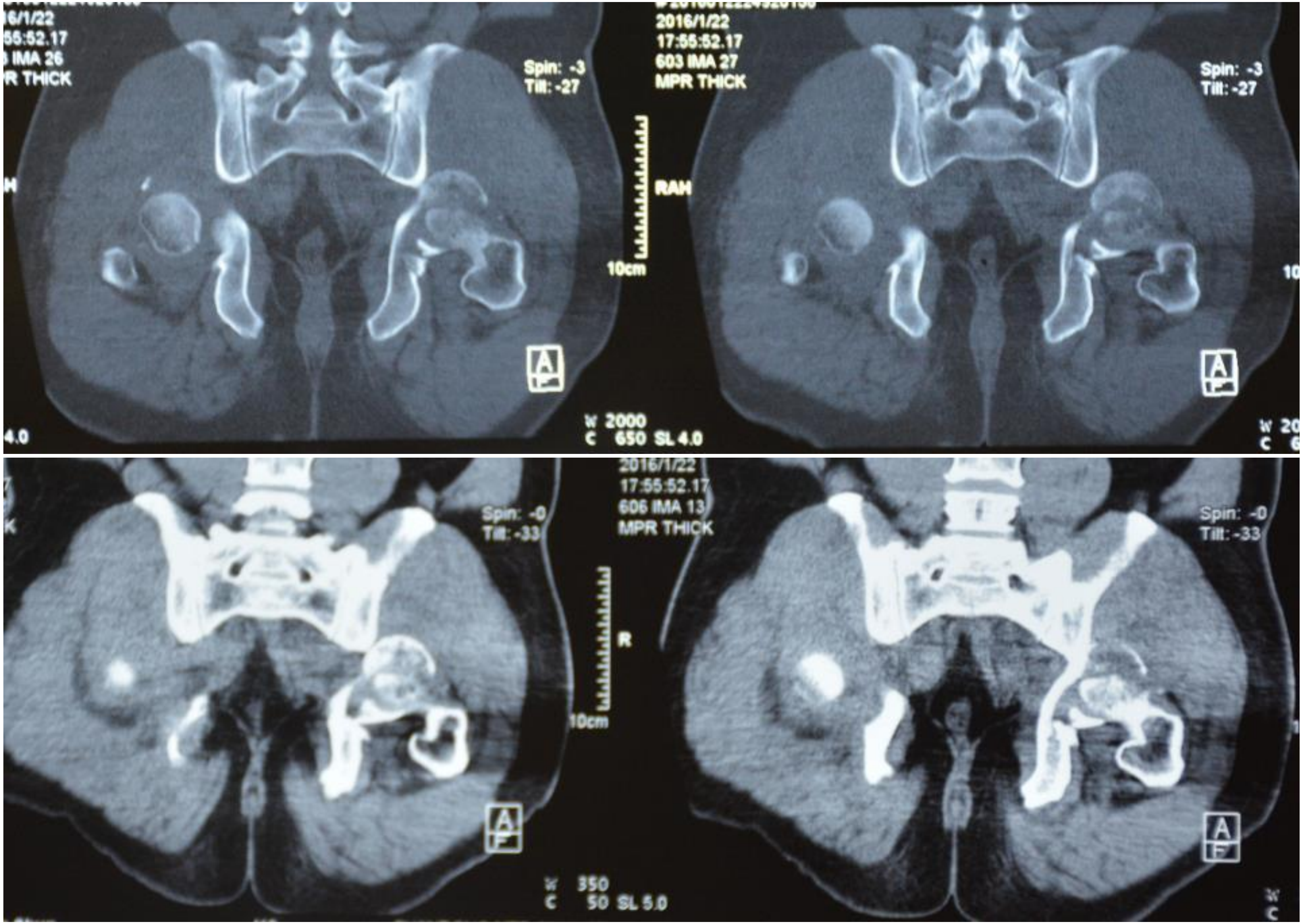
2016/1/22
17:55:52.17
603 IMA 27
MPR THICK
Spin: -3
Till: -27
RAH
10cm
W 2000
C 650 SL 4.0
2016/1/22
17:55:52.17
606 IMA 13
MPR THICK
Spin: -0
Till: -33
R
10cm
W 350
C 50 SL 5.0

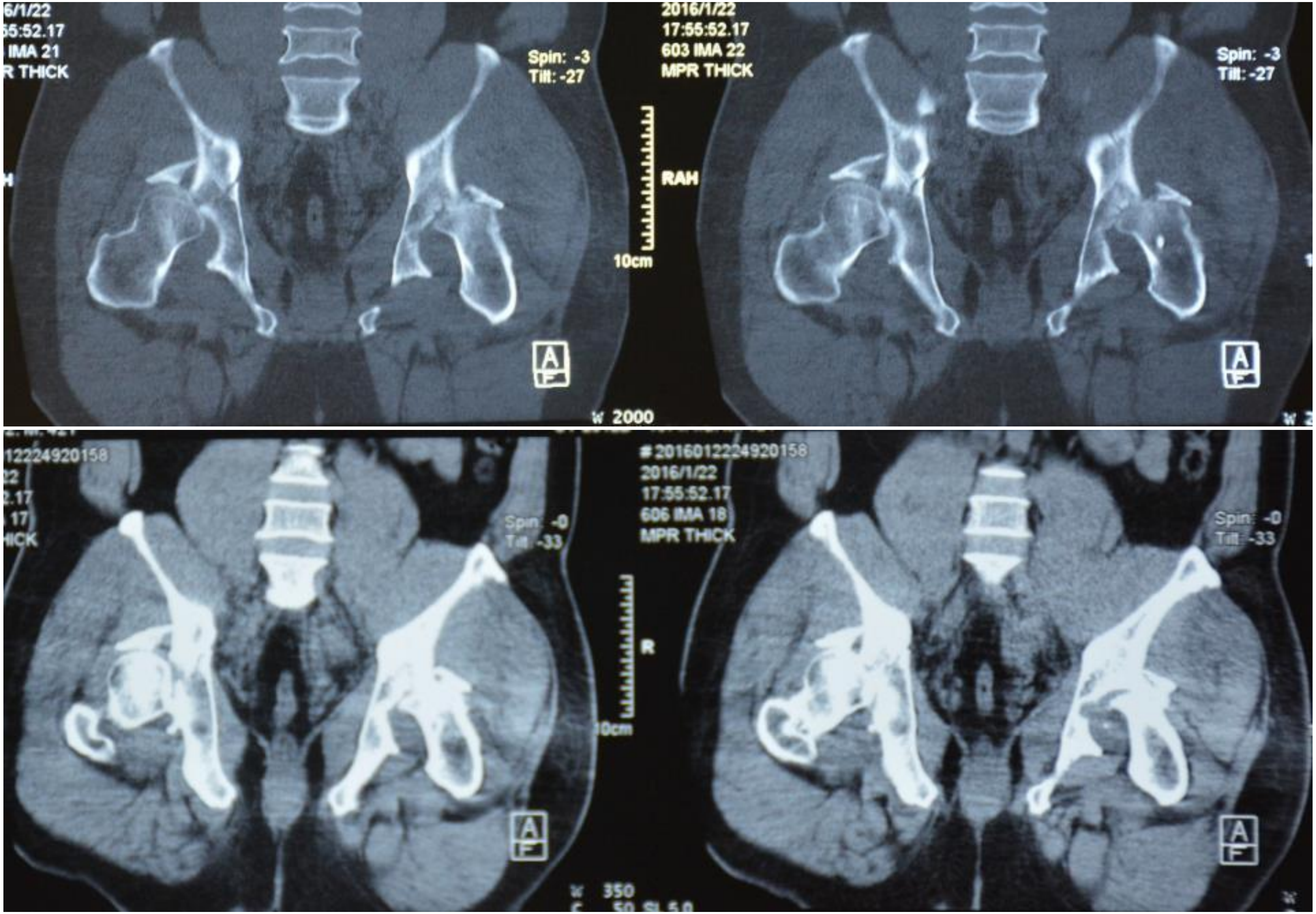
2016/1/22
17:55:52.17
603 IMA 22
MPR THICK
Spin: -3
Tilt: -27
RAH
10cm
W 2000
#20160122249 20158
2016/1/22
17:55:52.17
606 IMA 18
MPR THICK
Spin: -0
Tilt: -33
R
10cm
W 350

Left Femoral Head

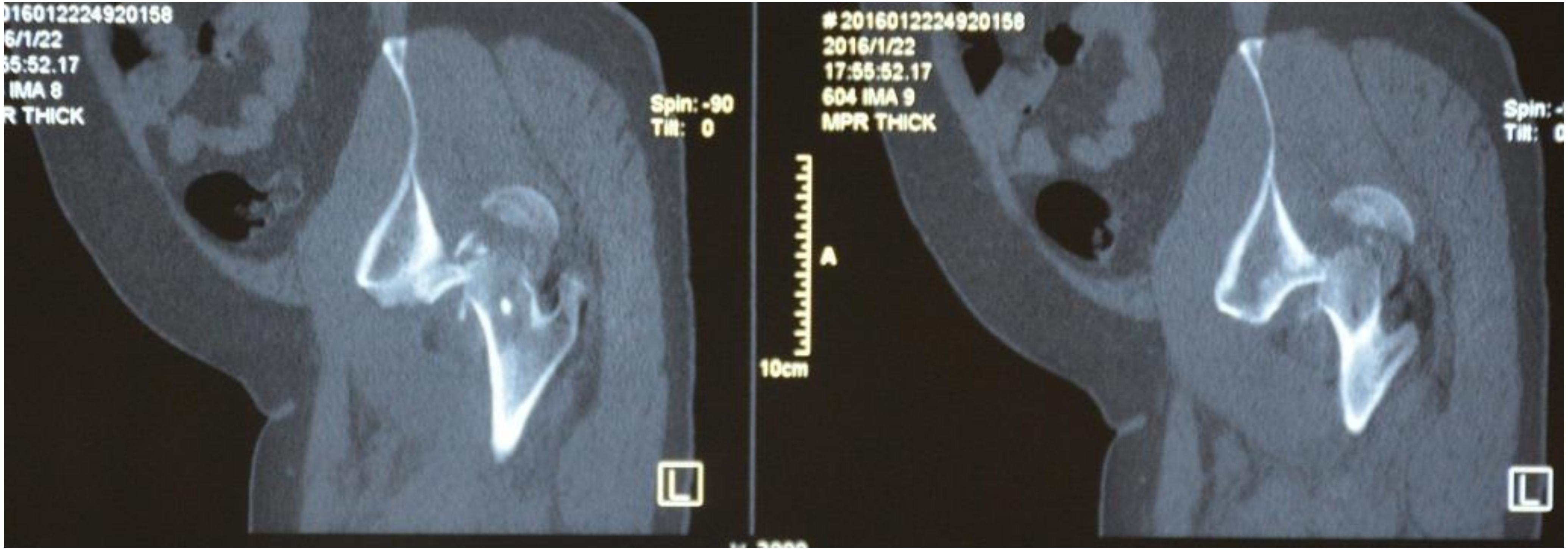

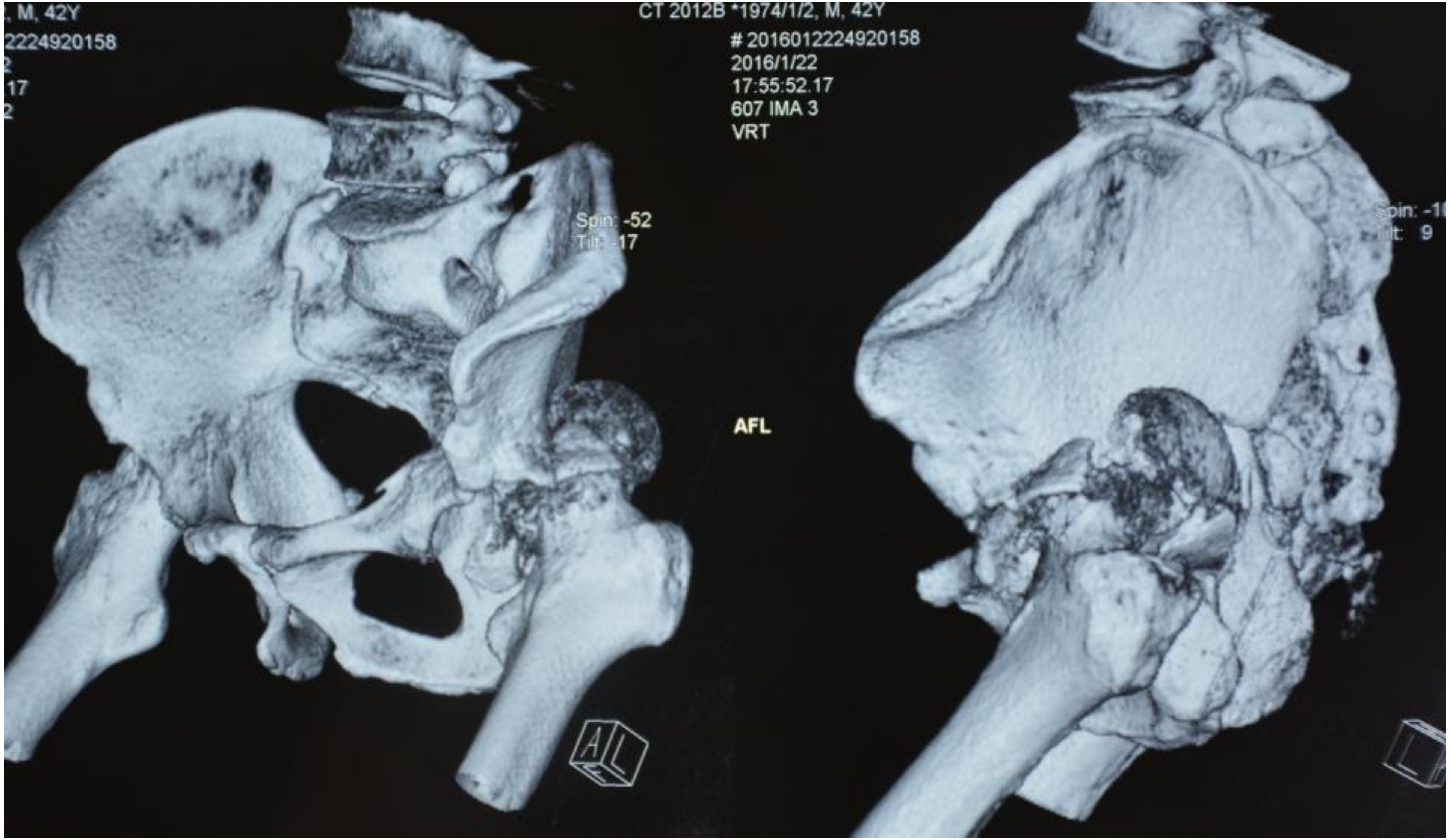
, M, 42Y
2224920158
17
CT 2012B *1974/1/2, M, 42Y
20160122249201 58
2016/1/22
17:55:52.17
607 IMA 3
VRT
Spin: -52
Tilt: -17
AFL
Tilt: 9

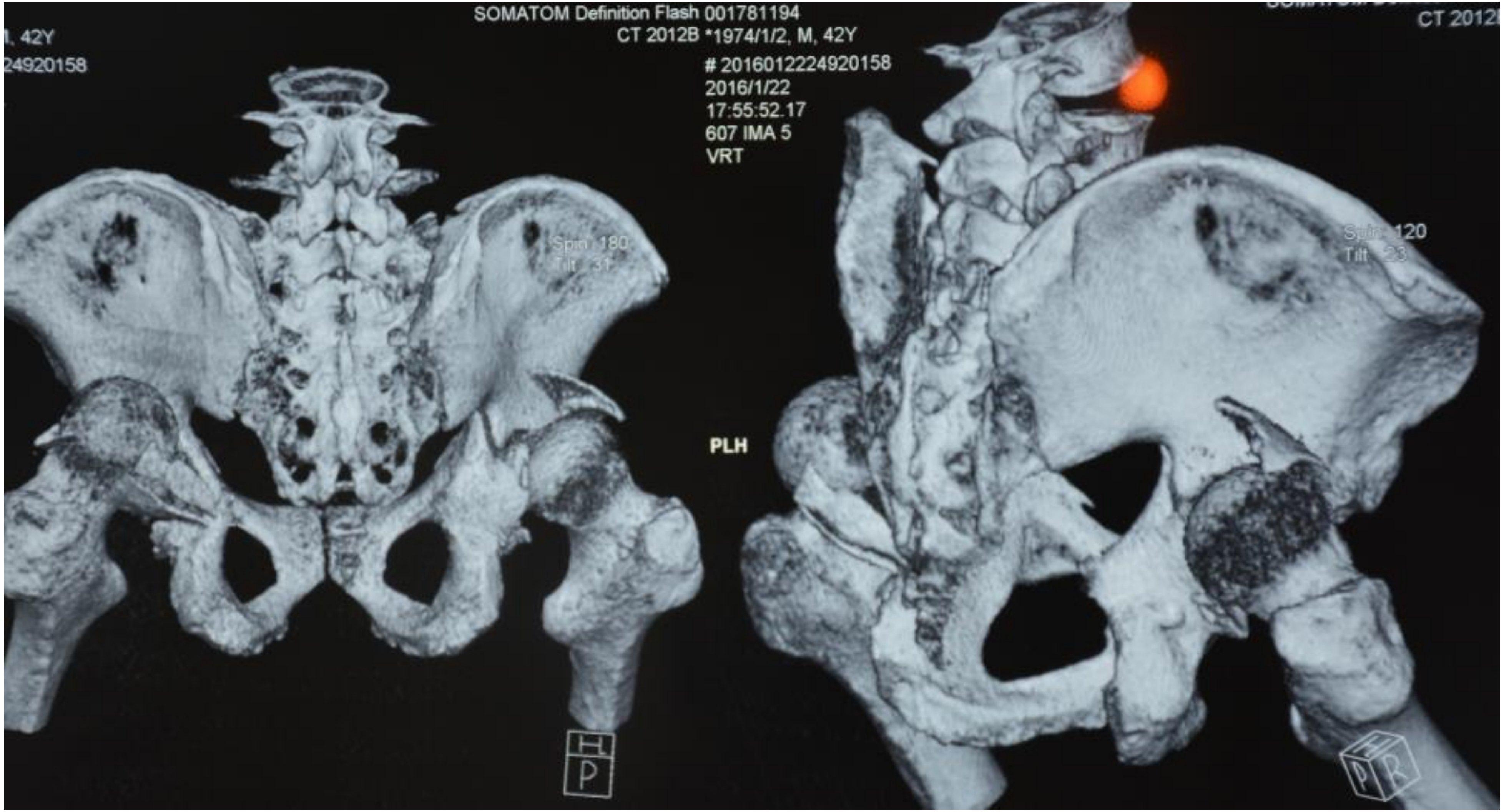
SOMATOM Definition Flash 001781194
CT 2012B *1974/1/2, M, 42Y
2016012224920158
2016/1/22
17:55:52.17
607 IMA 5
VRT
PLH
Spin 120
CT 2012

Intraoperative

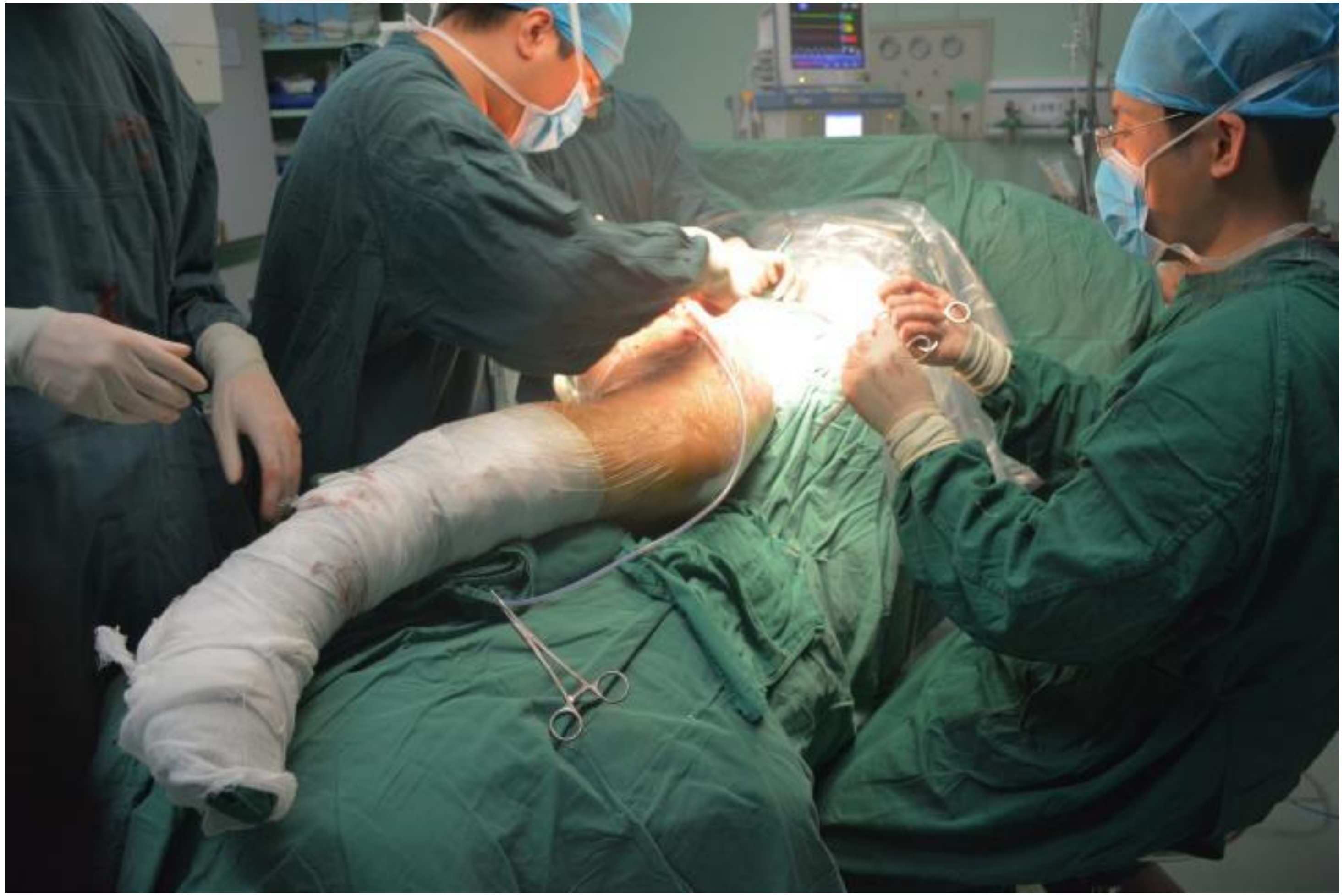

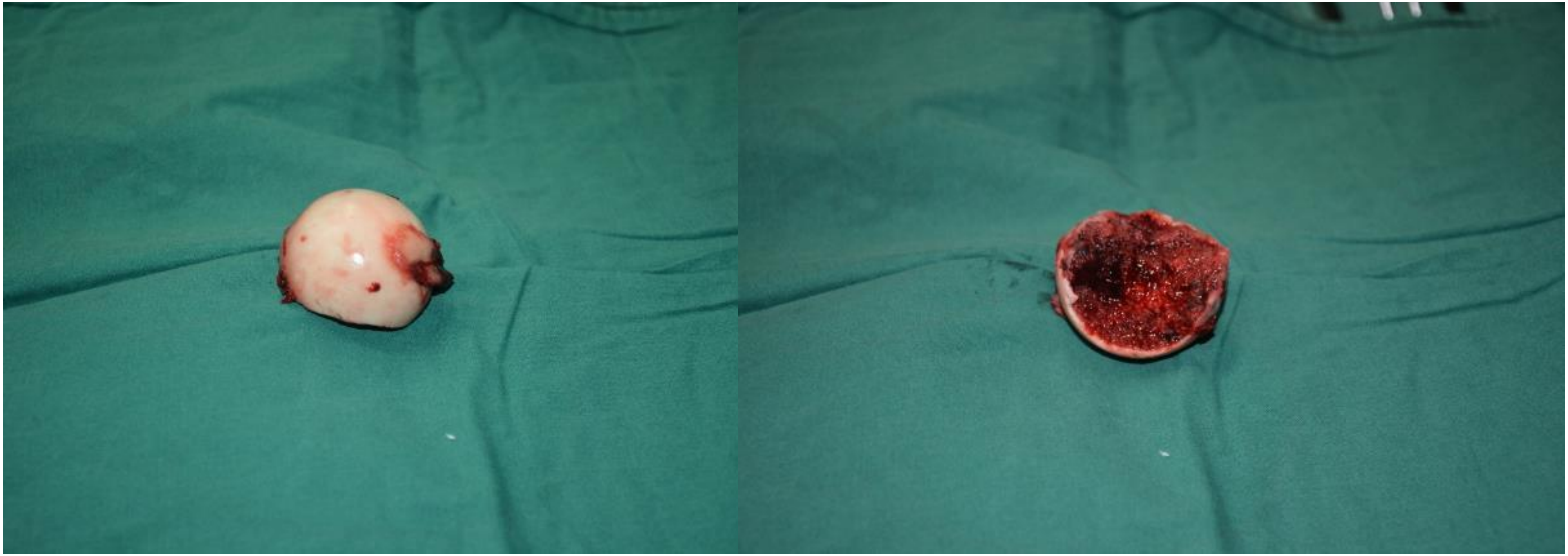

Postoperative

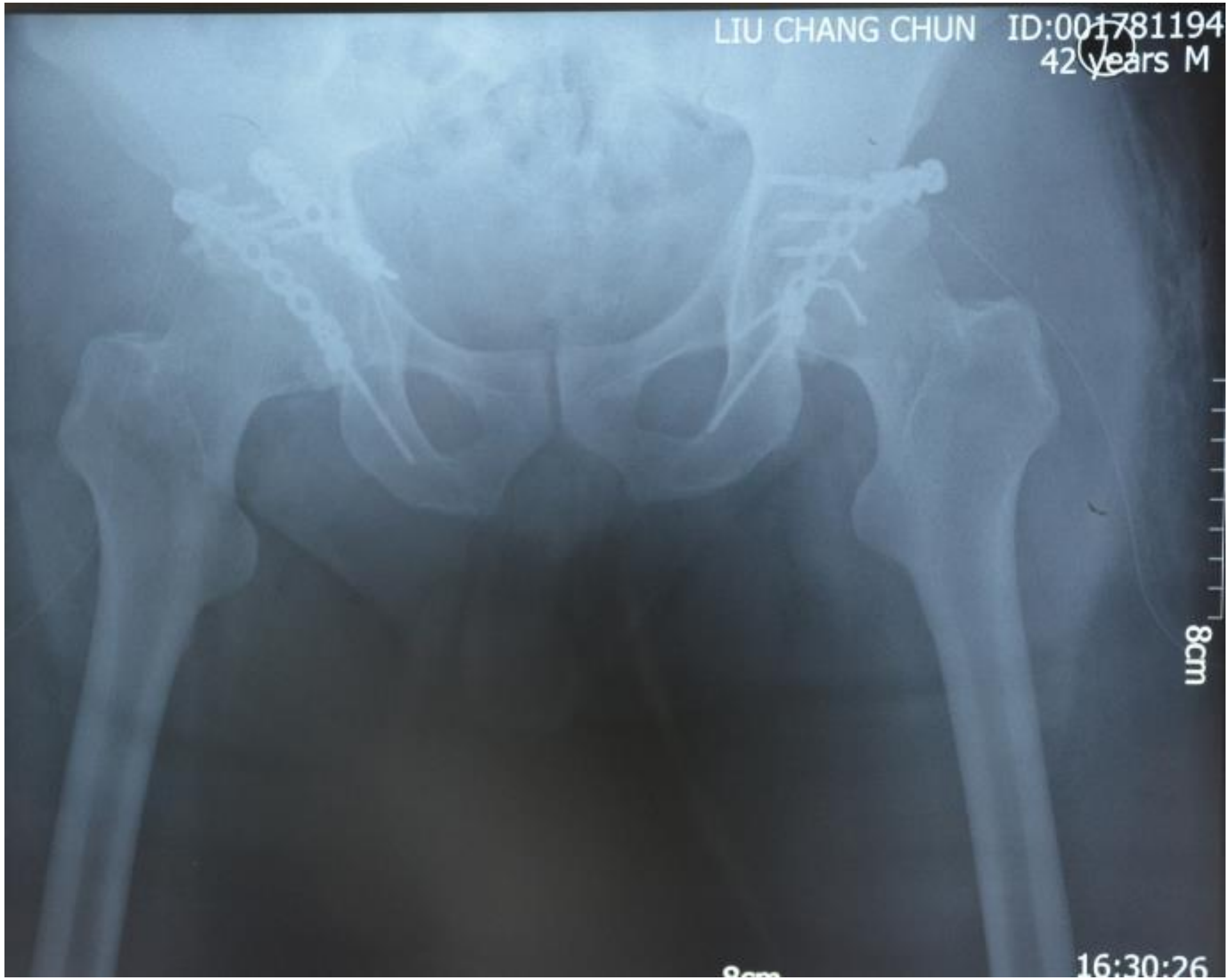

CASE 2

- -WANG, MALE, 46, Falling Injury, RIGHT

Preoperative

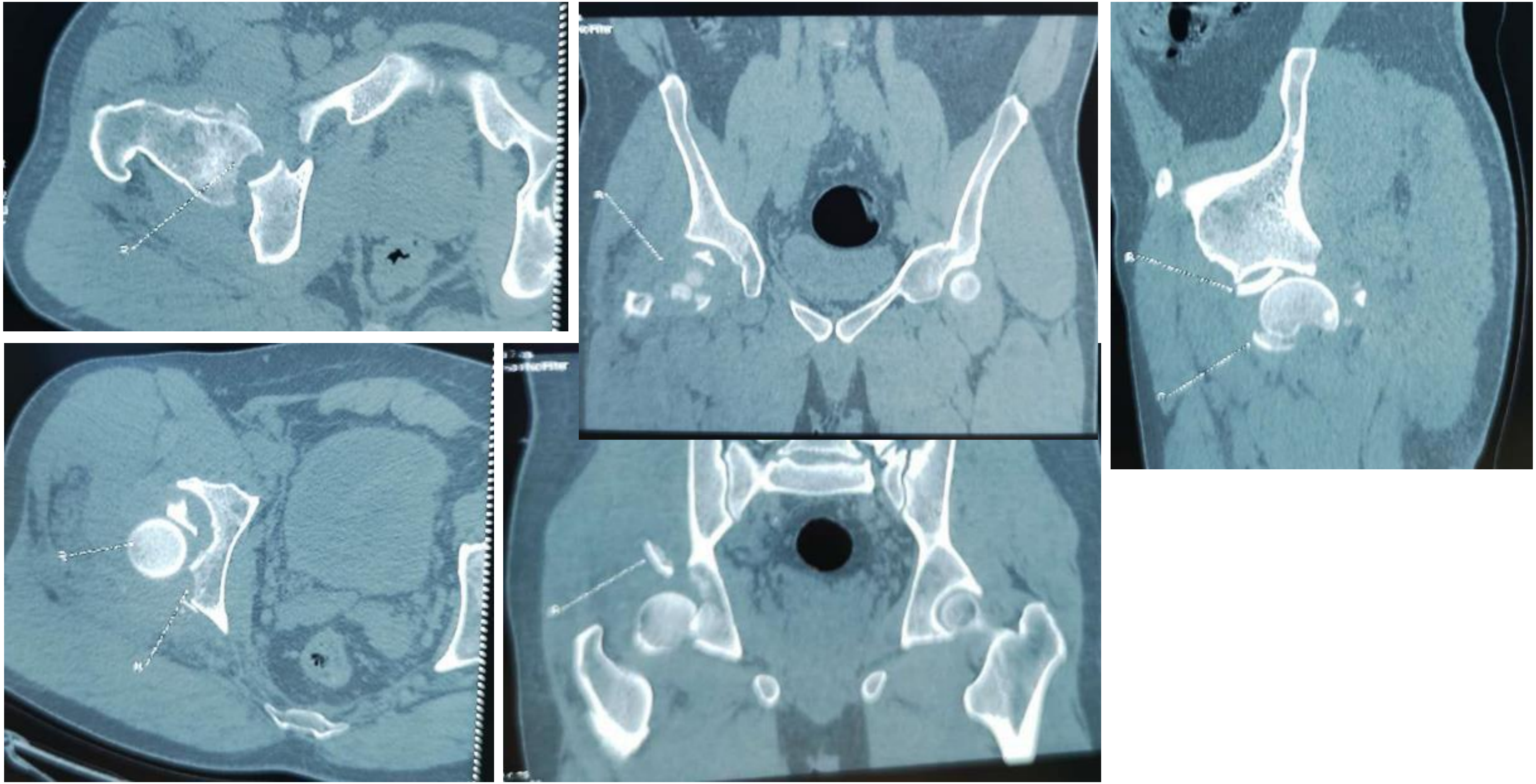

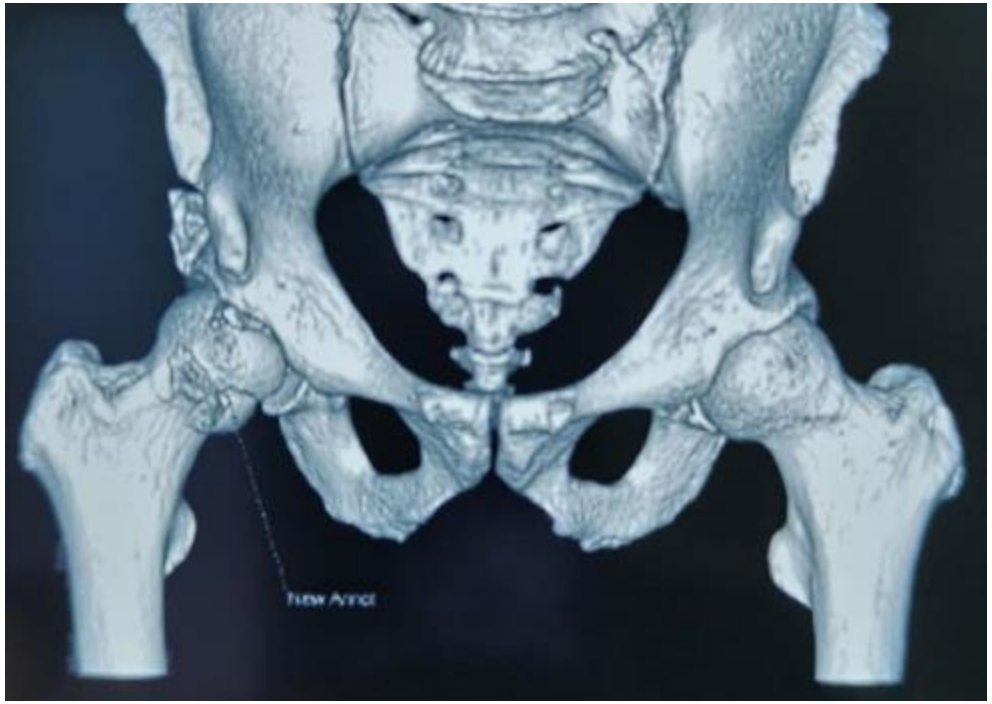

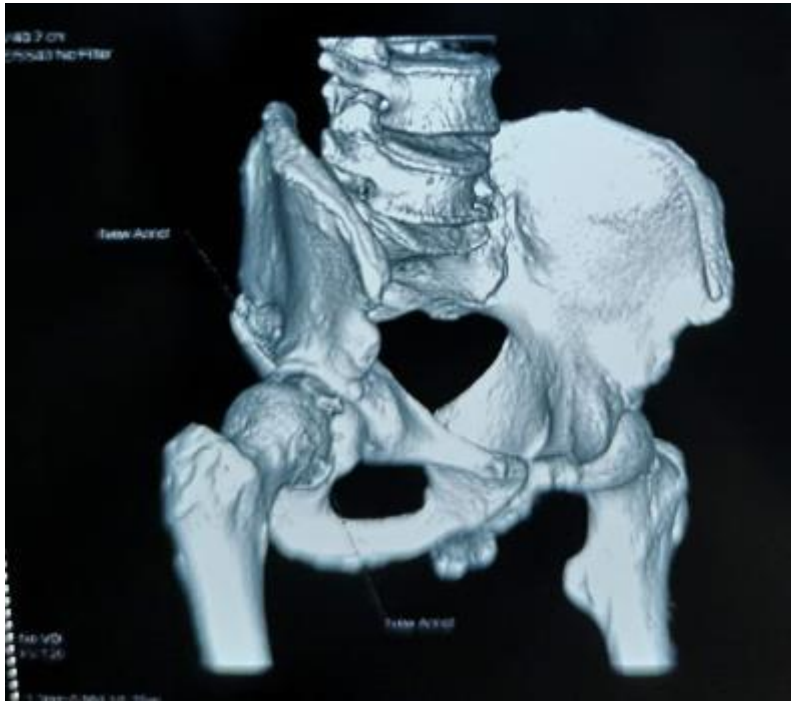

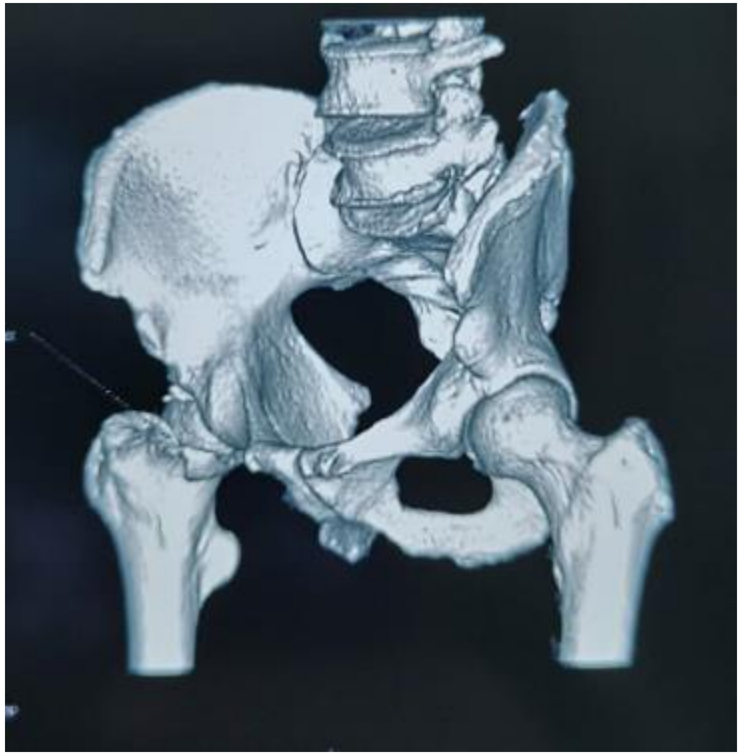

Intraoperative

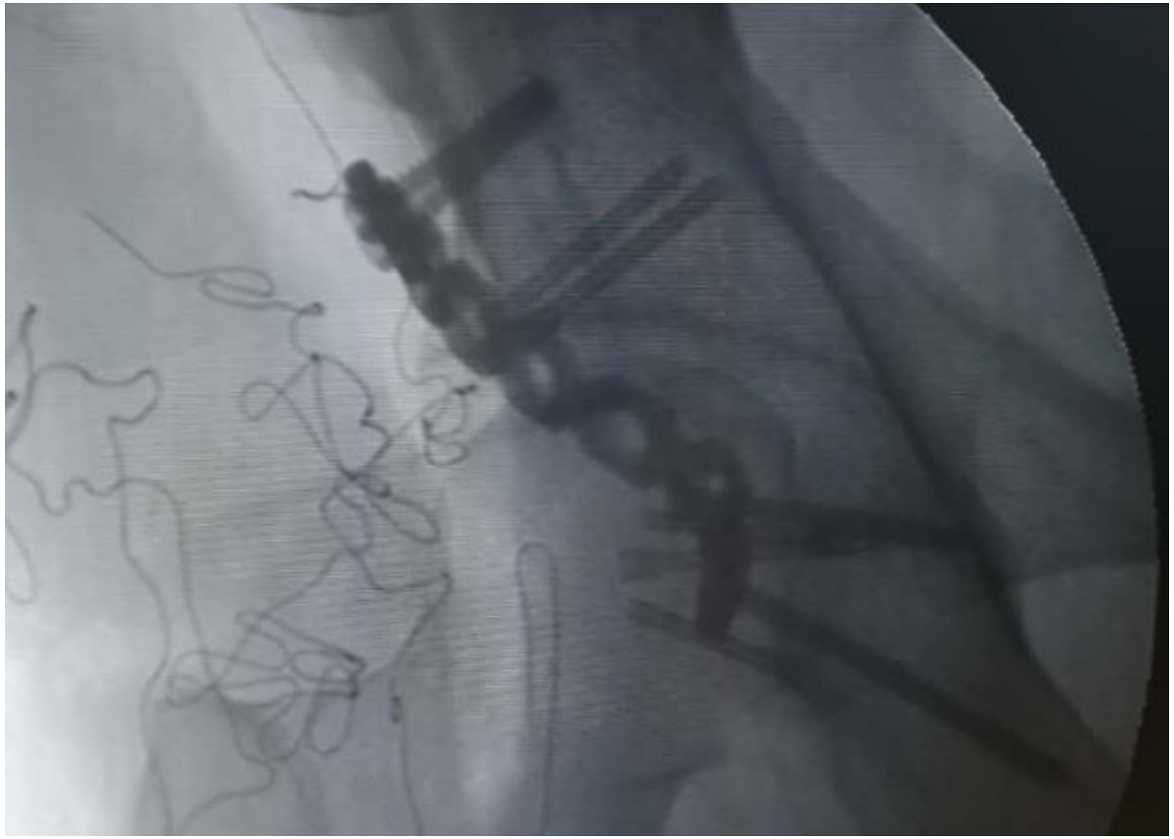

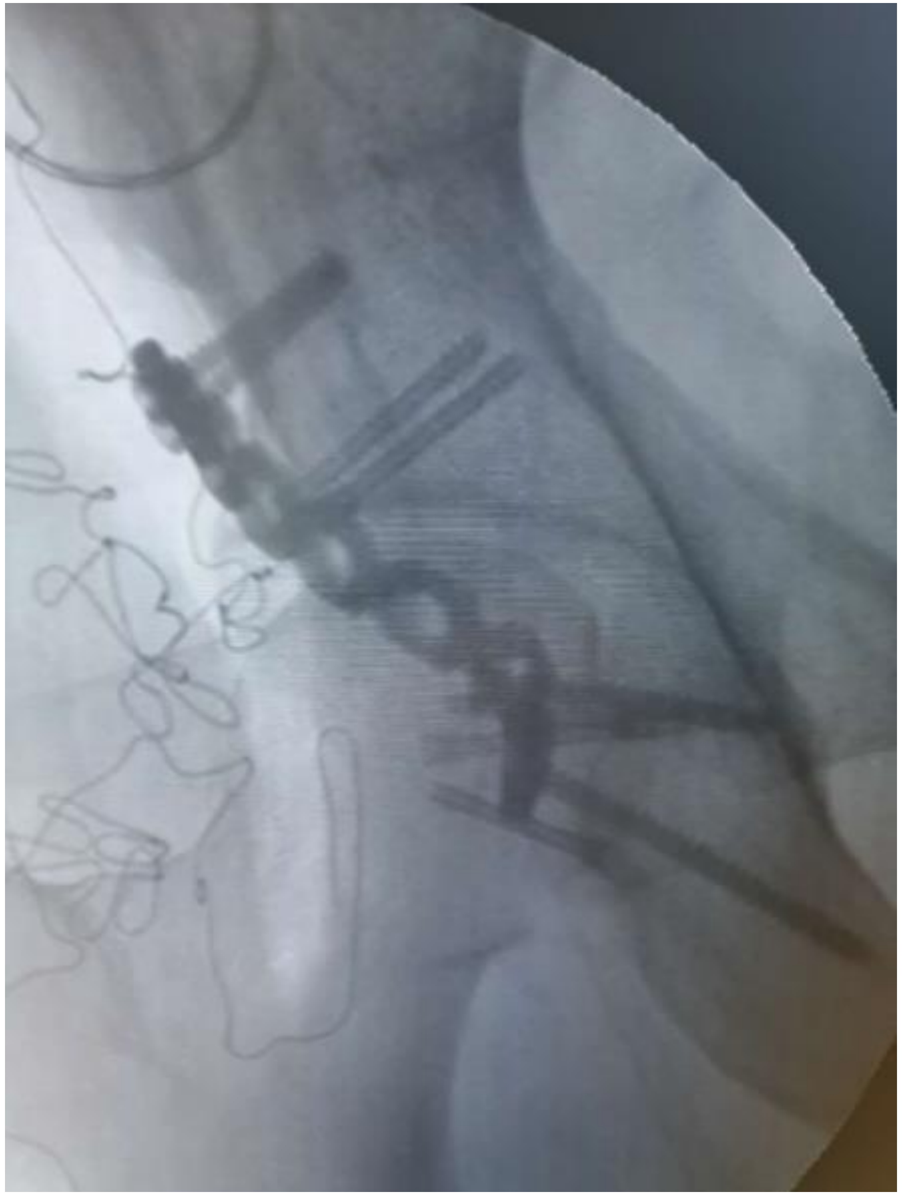

CASE 3

- -CHEN, MALE, 52Y, Falling Injury, RIGHT.

Preoperative

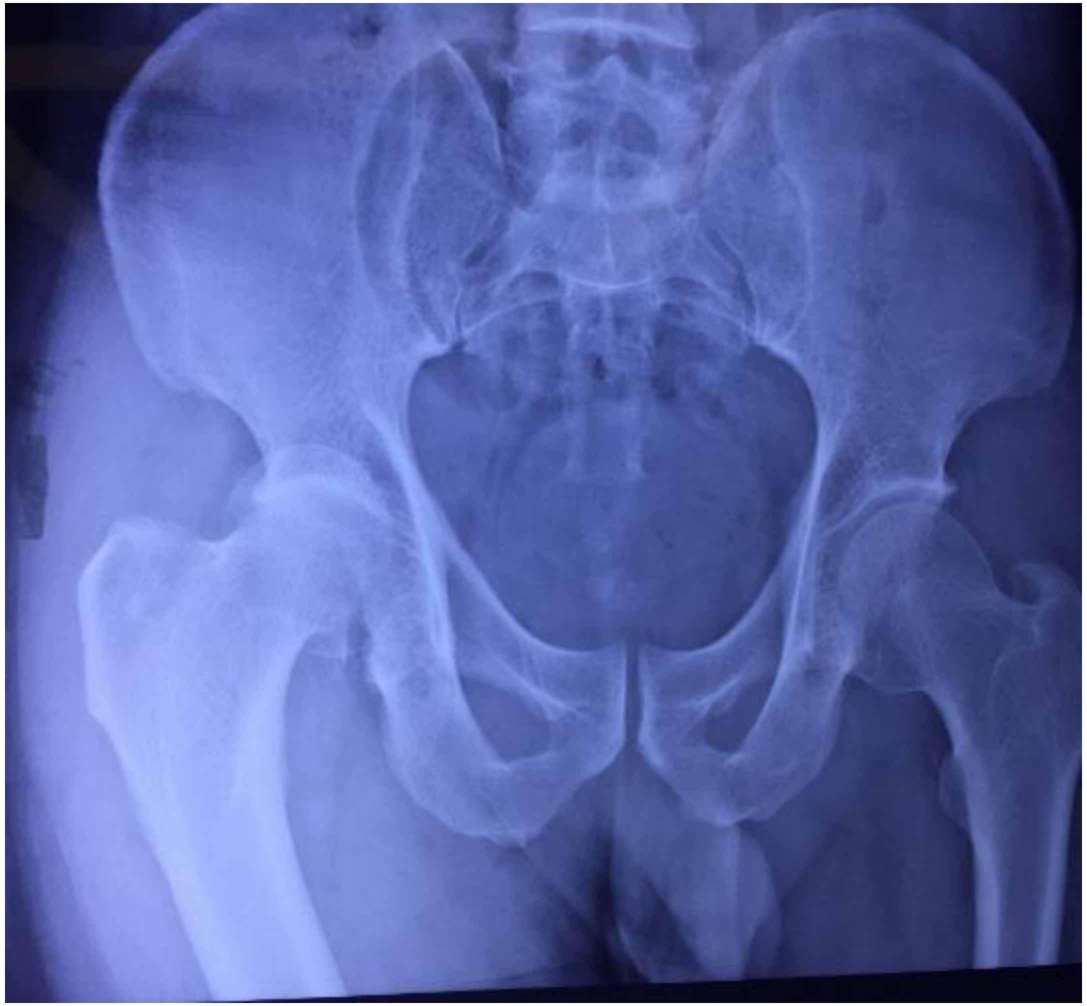

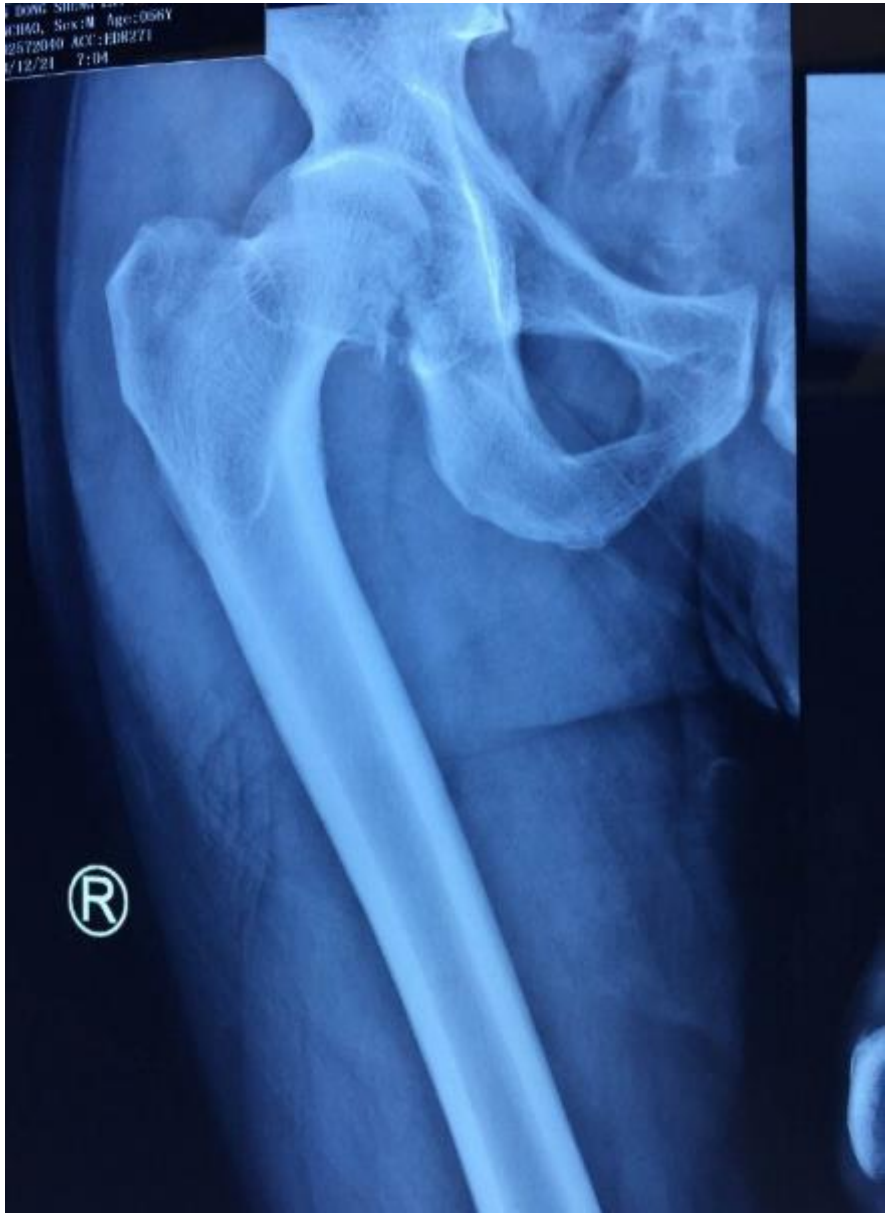

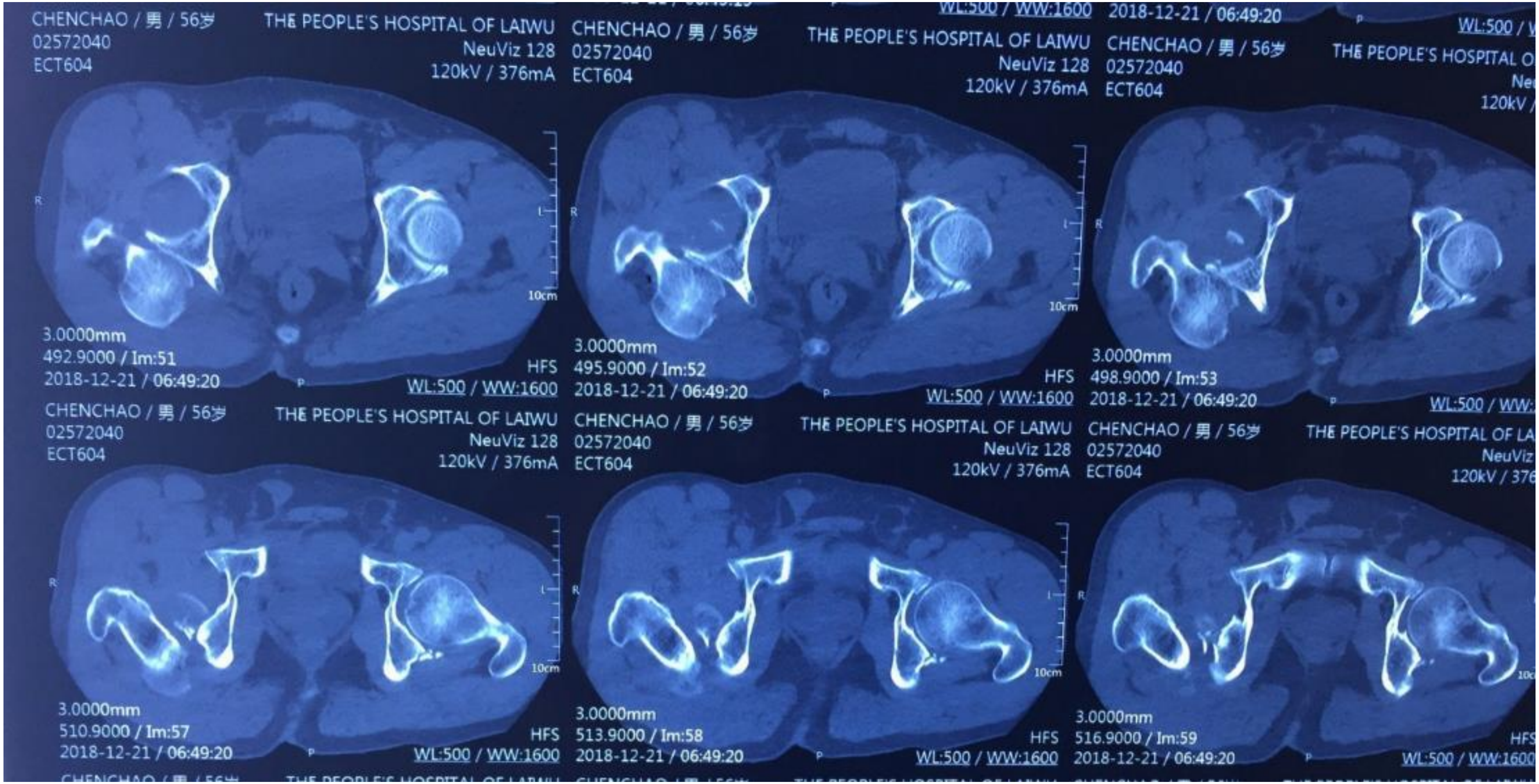
CHENCHAO / 男 / 56岁
02572040
ECT604
THE PEOPLE'S HOSPITAL OF LAIWU
NeuViz 128
120kV / 376mA
10cm
3.0000mm
492.9000 / Im:51
2018-12-21 / 06:49:20
HFS
WL:500 / WW:1600
CHENCHAO / 男 / 56岁
02572040
ECT604
THE PEOPLE'S HOSPITAL OF LAIWU
NeuViz 128
120kV / 376mA
10cm
3.0000mm
495.9000 / Im:52
2018-12-21 / 06:49:20
HFS
WL:500 / WW:1600
2018-12-21 / 06:49:20
CHENCHAO / 男 / 56岁
02572040
ECT604
3.0000mm
498.9000 / Im:53
2018-12-21 / 06:49:20
CHENCHAO / 男 / 56岁
02572040
ECT604
THE PEOPLE'S HOSPITAL OF LAIWU
NeuViz 128
120kV / 376mA
10cm
3.0000mm
510.9000 / Im:57
2018-12-21 / 06:49:20
HFS
WL:500 / WW:1600
CHENCHAO / 男 / 56岁
02572040
ECT604
THE PEOPLE'S HOSPITAL OF LAIWU
NeuViz 128
120kV / 376mA
10cm
3.0000mm
513.9000 / Im:58
2018-12-21 / 06:49:20
HFS
WL:500 / WW:1600
CHENCHAO / 男 / 56岁
02572040
ECT604
3.0000mm
516.9000 / Im:59
2018-12-21 / 06:49:20
WL:500 / WW:1600

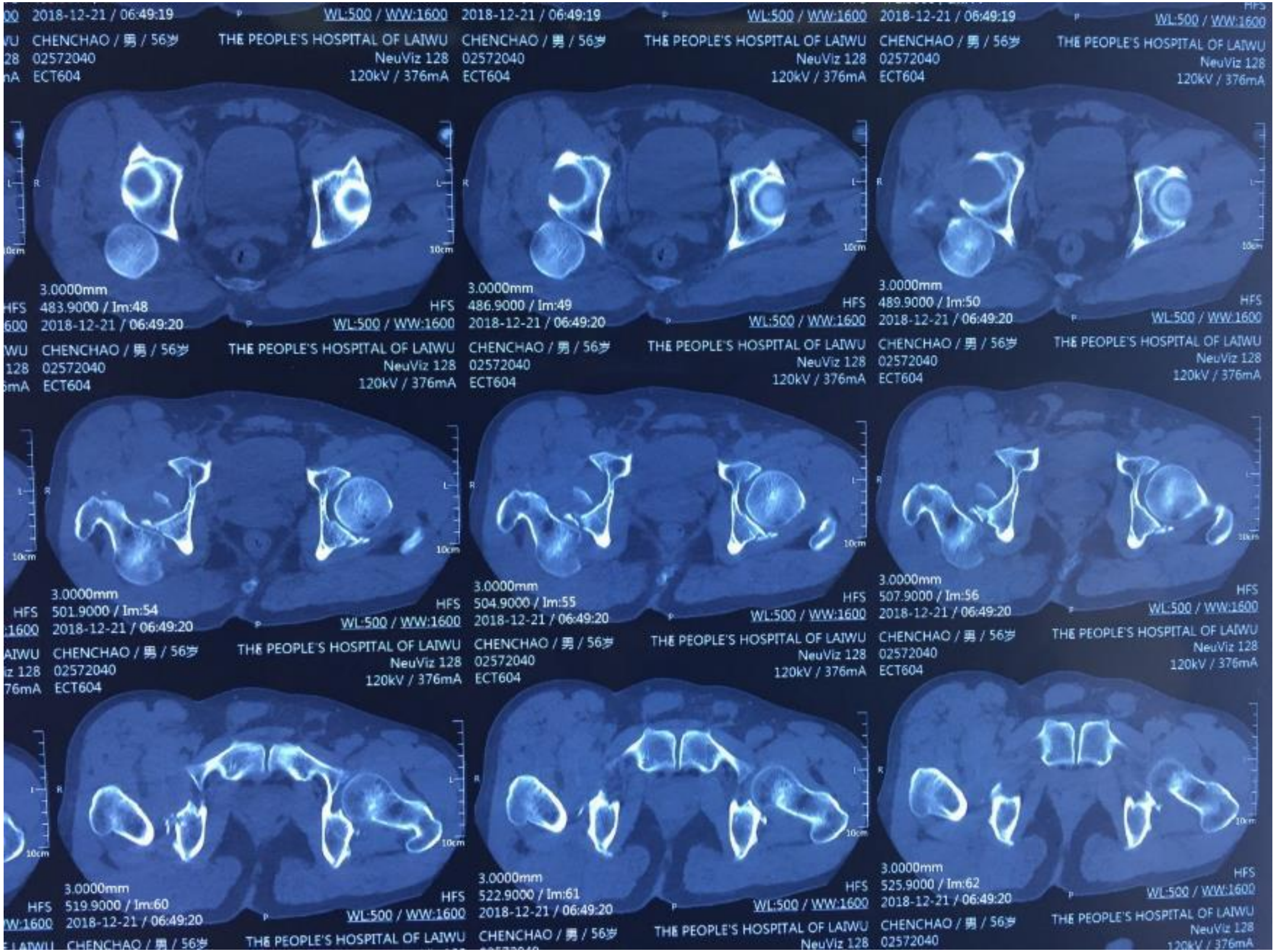
2018-12-21 / 06:49:19
WL:500 / WW:1600
CHENCHAO / 男 / 56岁
02572040
ECT604
THE PEOPLE'S HOSPITAL OF LAIWU
NeuViz 128
120kV / 376mA
2018-12-21 / 06:49:19
WL:500 / WW:1600
CHENCHAO / 男 / 56岁
02572040
ECT604
THE PEOPLE'S HOSPITAL OF LAIWU
NeuViz 128
120kV / 376mA
2018-12-21 / 06:49:19
WL:500 / WW:1600
CHENCHAO / 男 / 56岁
02572040
ECT604
THE PEOPLE'S HOSPITAL OF LAIWU
NeuViz 128
120kV / 376mA
3.0000mm
483.9000 / Im:48
2018-12-21 / 06:49:20
HFS
WL:500 / WW:1600
CHENCHAO / 男 / 56岁
02572040
ECT604
THE PEOPLE'S HOSPITAL OF LAIWU
NeuViz 128
120kV / 376mA
3.0000mm
486.9000 / Im:49
2018-12-21 / 06:49:20
HFS
WL:500 / WW:1600
CHENCHAO / 男 / 56岁
02572040
ECT604
THE PEOPLE'S HOSPITAL OF LAIWU
NeuViz 128
120kV / 376mA
3.0000mm
489.9000 / Im:50
2018-12-21 / 06:49:20
HFS
WL:500 / WW:1600
CHENCHAO / 男 / 56岁
02572040
ECT604
THE PEOPLE'S HOSPITAL OF LAIWU
NeuViz 128
120kV / 376mA
3.0000mm
501.9000 / Im:54
2018-12-21 / 06:49:20
HFS
WL:500 / WW:1600
CHENCHAO / 男 / 56岁
02572040
ECT604
THE PEOPLE'S HOSPITAL OF LAIWU
NeuViz 128
120kV / 376mA
3.0000mm
504.9000 / Im:55
2018-12-21 / 06:49:20
HFS
WL:500 / WW:1600
CHENCHAO / 男 / 56岁
02572040
ECT604
THE PEOPLE'S HOSPITAL OF LAIWU
NeuViz 128
120kV / 376mA
3.0000mm
507.9000 / Im:56
2018-12-21 / 06:49:20
HFS
WL:500 / WW:1600
CHENCHAO / 男 / 56岁
02572040
ECT604
THE PEOPLE'S HOSPITAL OF LAIWU
NeuViz 128
120kV / 376mA
3.0000mm
519.9000 / Im:60
2018-12-21 / 06:49:20
HFS
WL:500 / WW:1600
CHENCHAO / 男 / 56岁
THE PEOPLE'S HOSPITAL OF LAIWU
3.0000mm
522.9000 / Im:61
2018-12-21 / 06:49:20
HFS
WL:500 / WW:1600
CHENCHAO / 男 / 56岁
THE PEOPLE'S HOSPITAL OF LAIWU
NeuViz 128
3.0000mm
525.9000 / Im:62
2018-12-21 / 06:49:20
HFS
WL:500 / WW:1600
CHENCHAO / 男 / 56岁
02572040
THE PEOPLE'S HOSPITAL OF LAIWU
NeuViz 128
120kV / 376mA

3D-CT, Preoperative

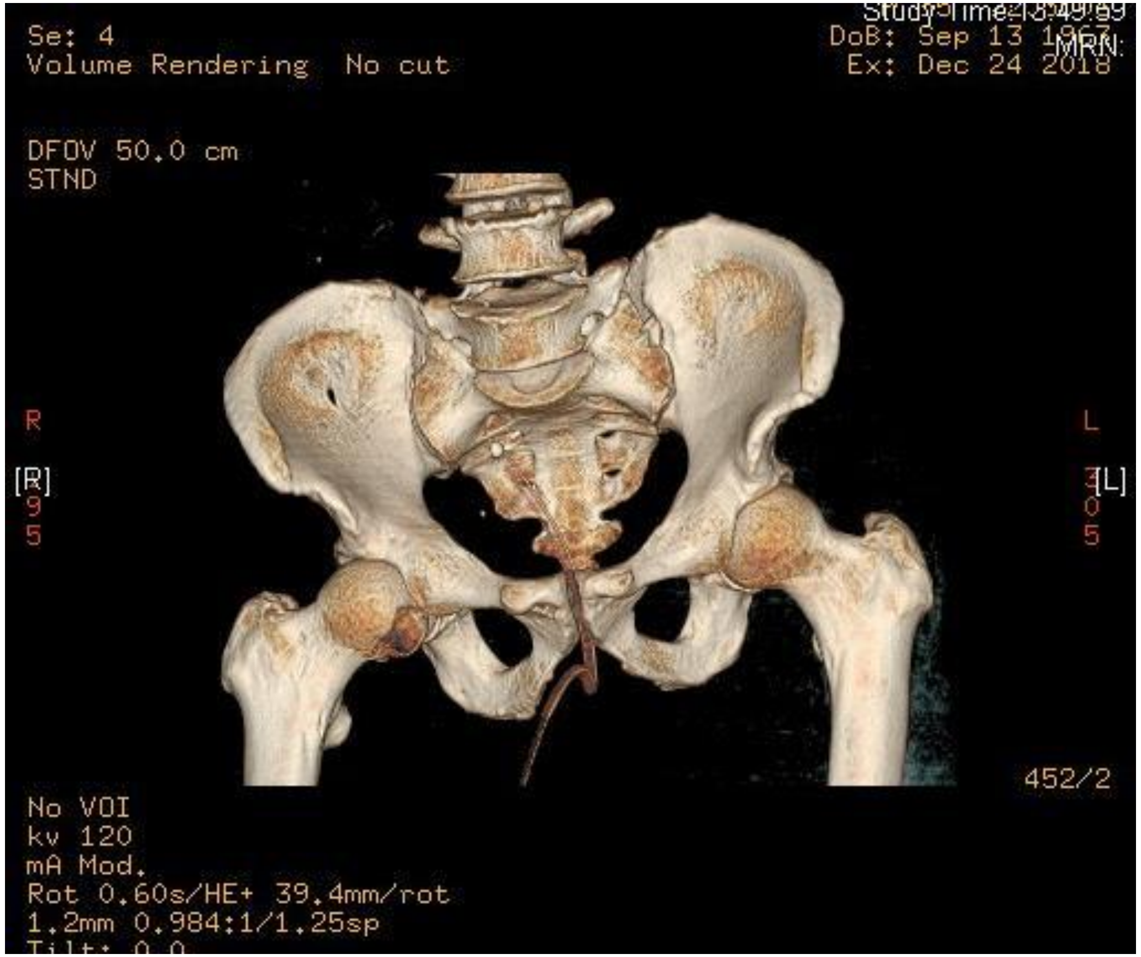

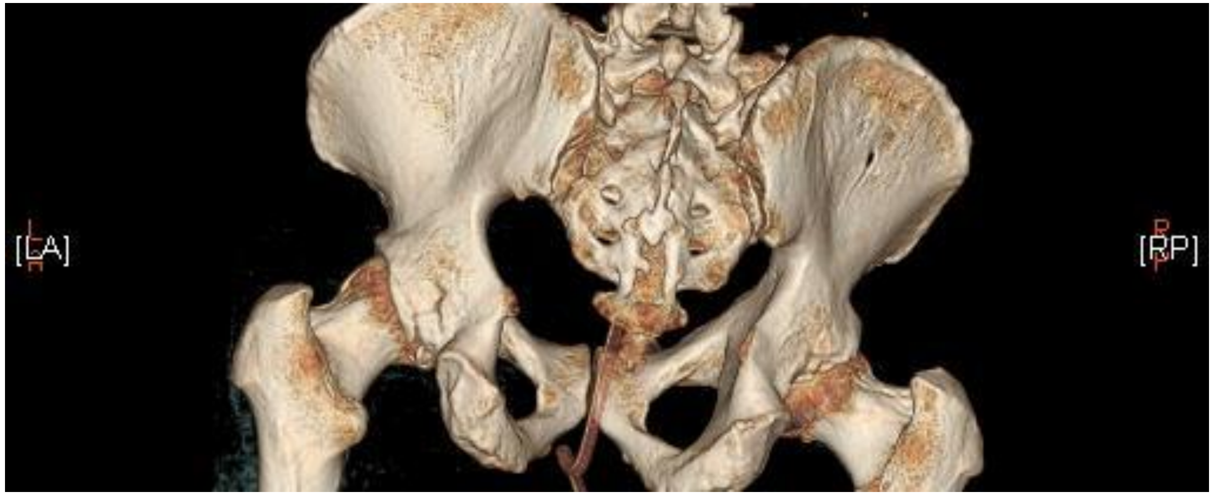

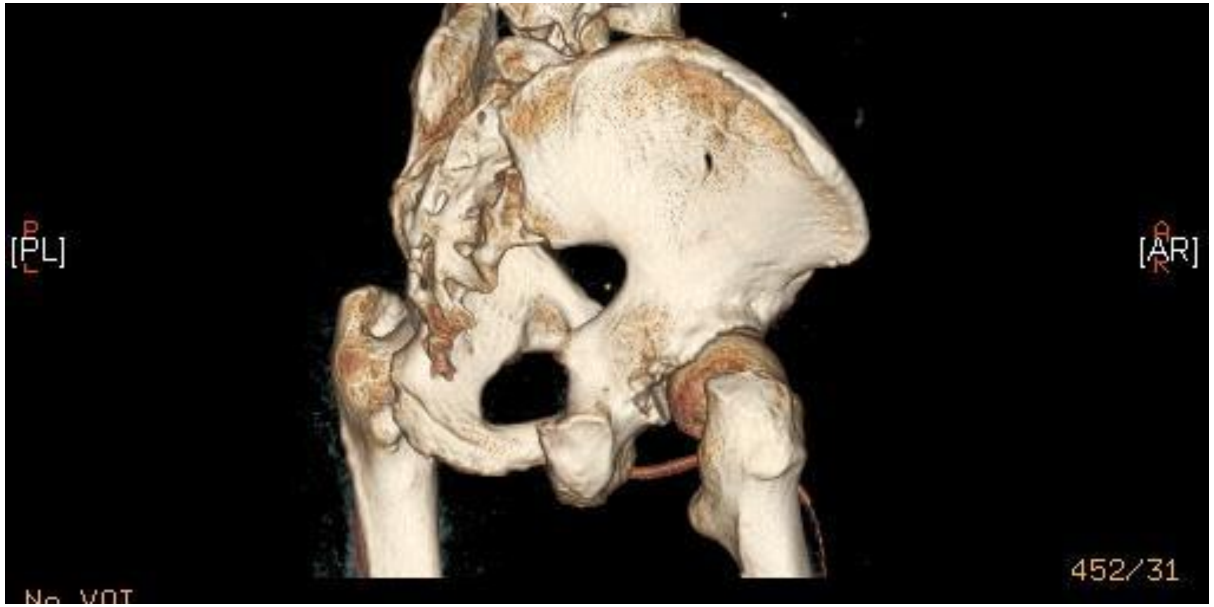

Intraoperative

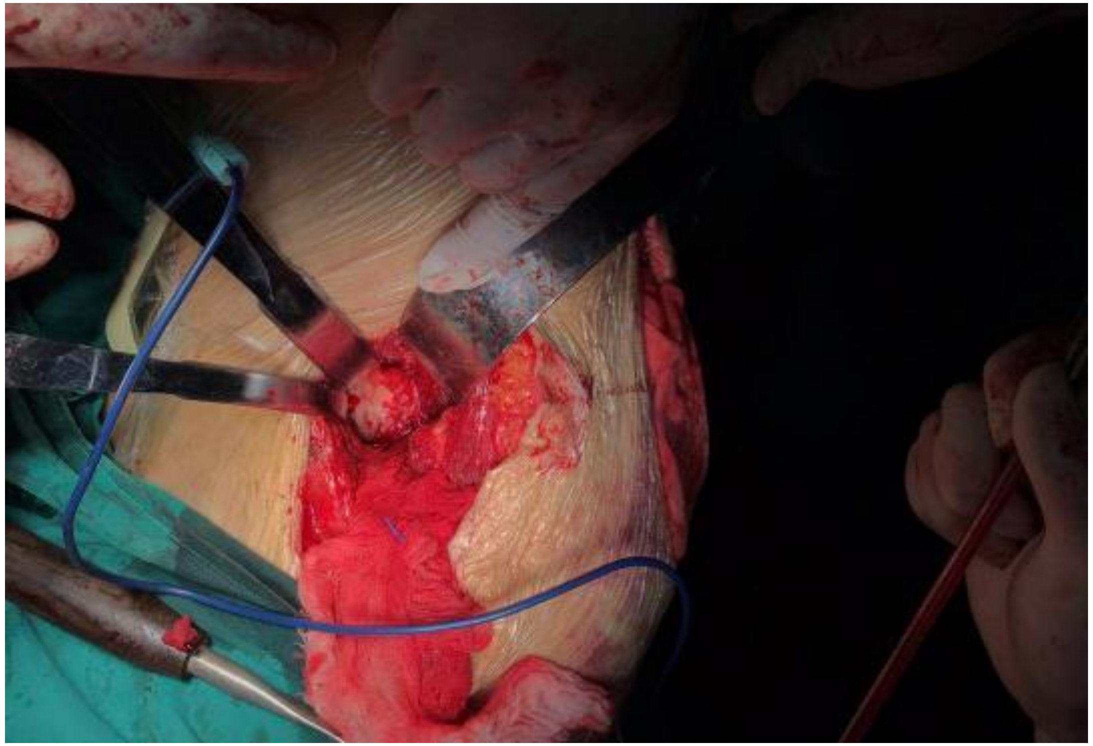

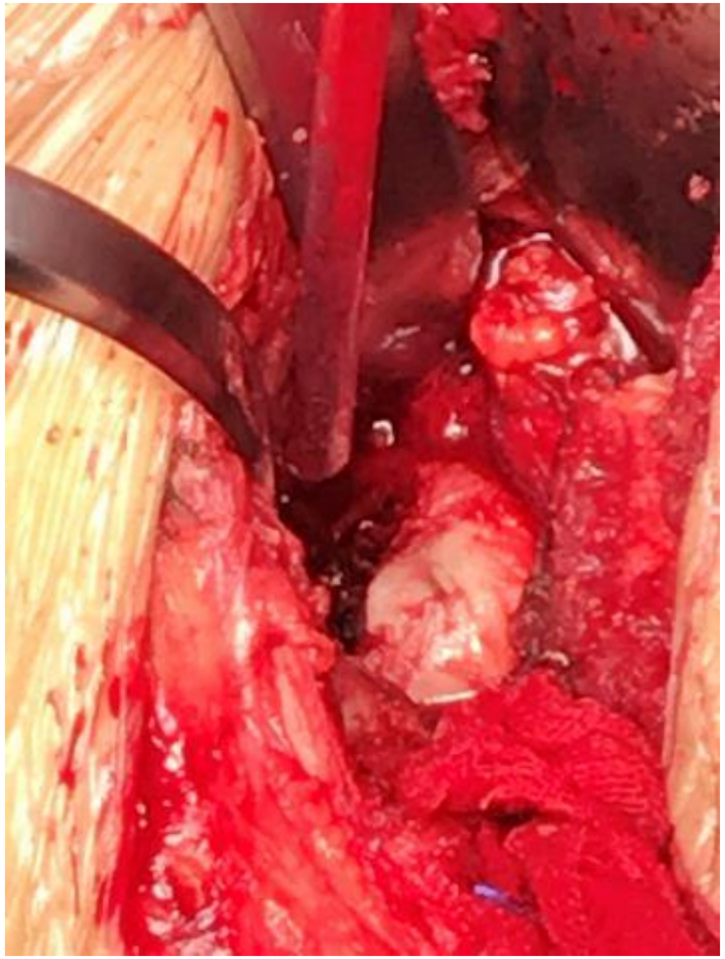

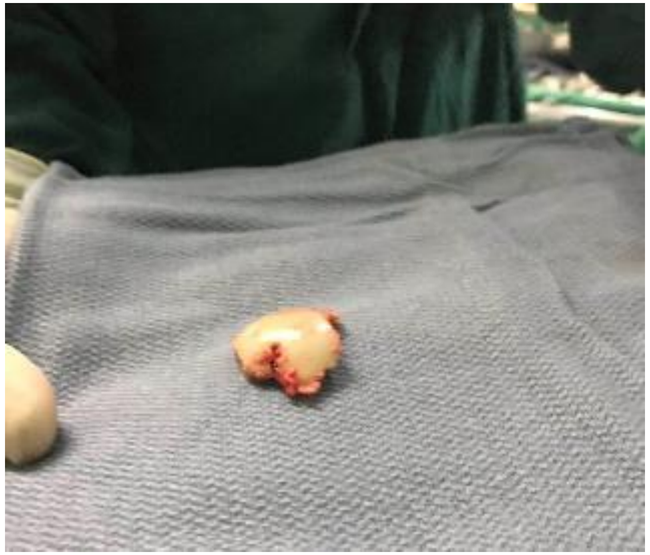

Postoperative

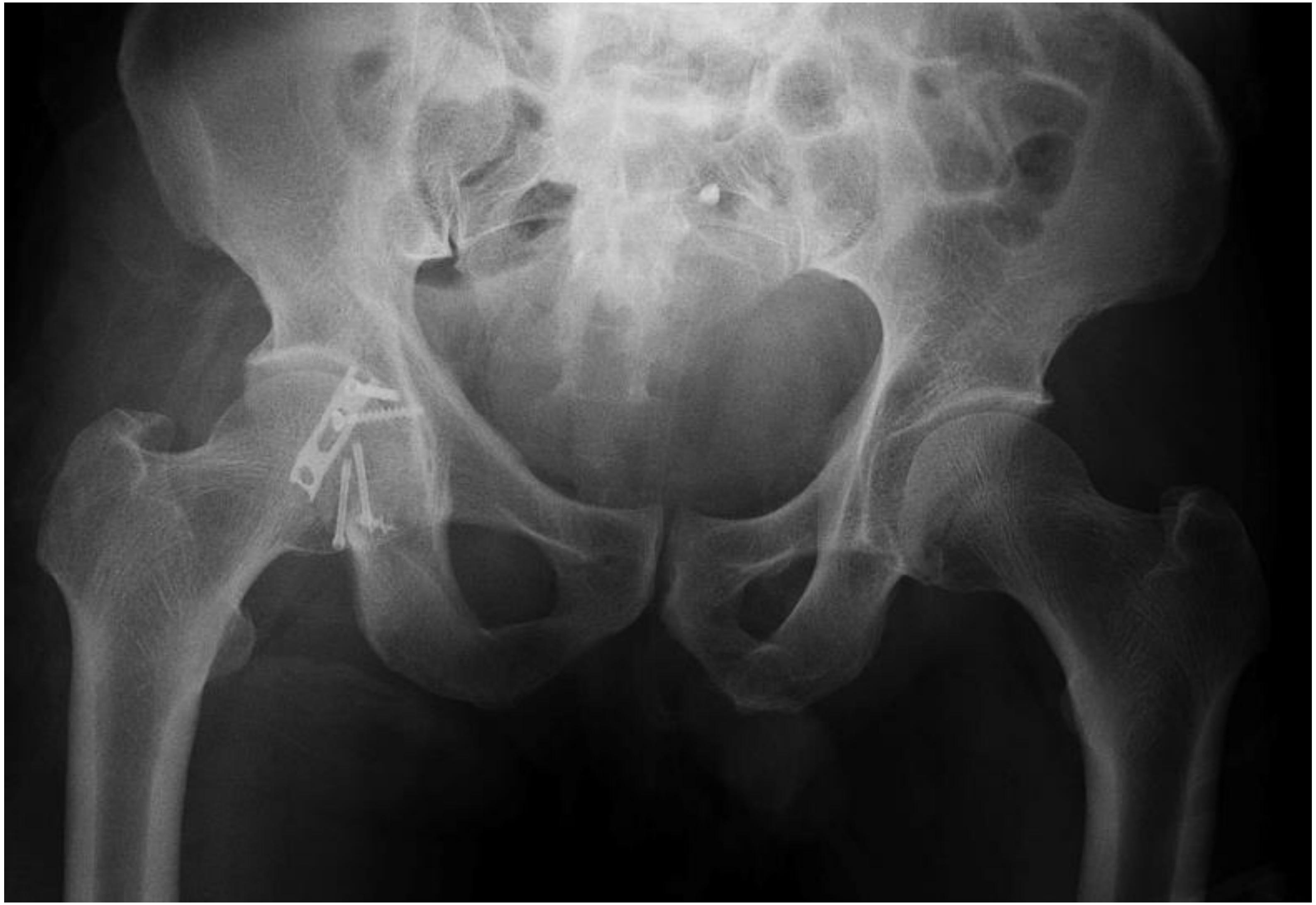

1Month, Postoperative

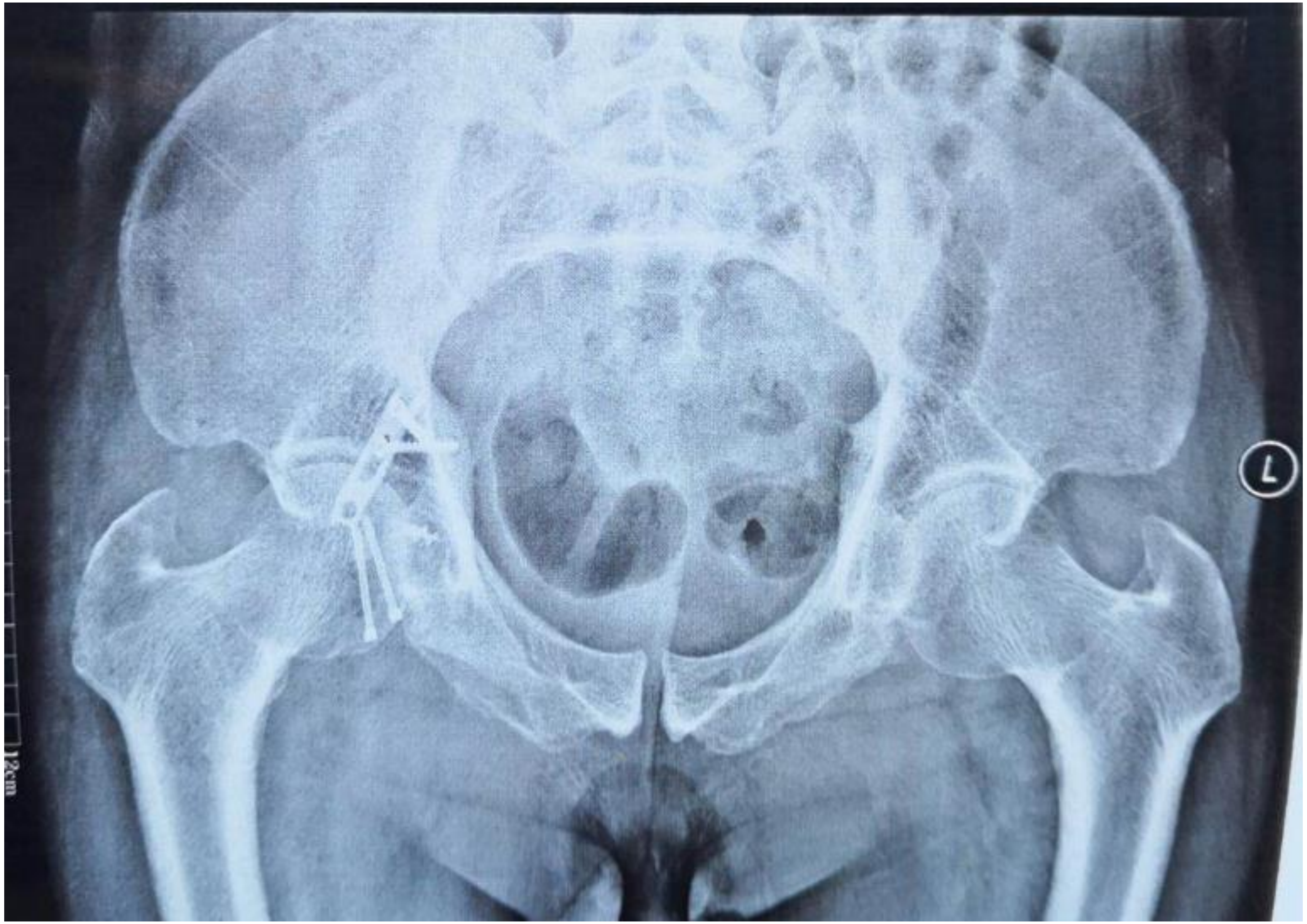

1 Year, Postoperative

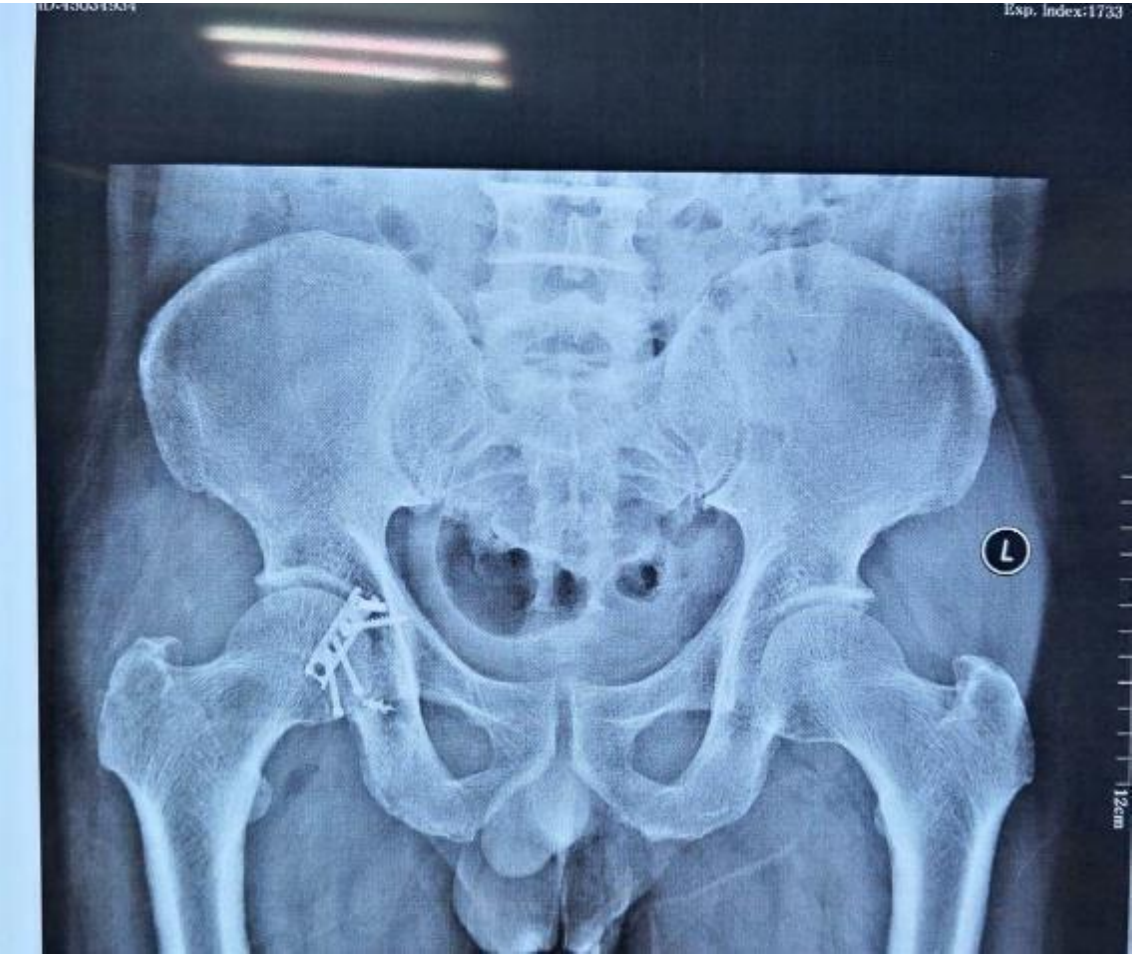

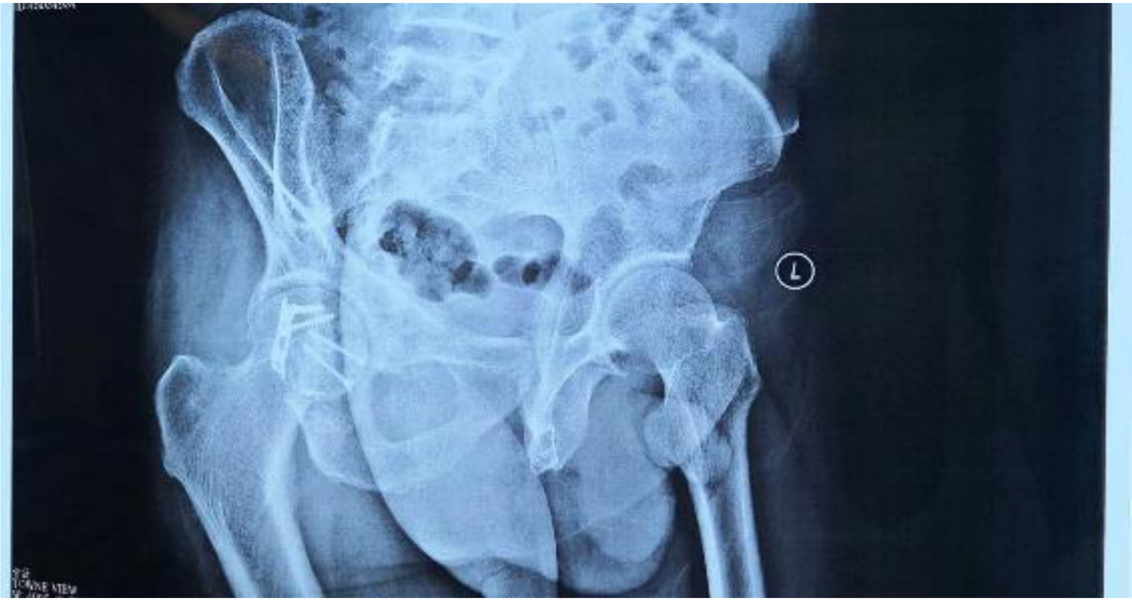

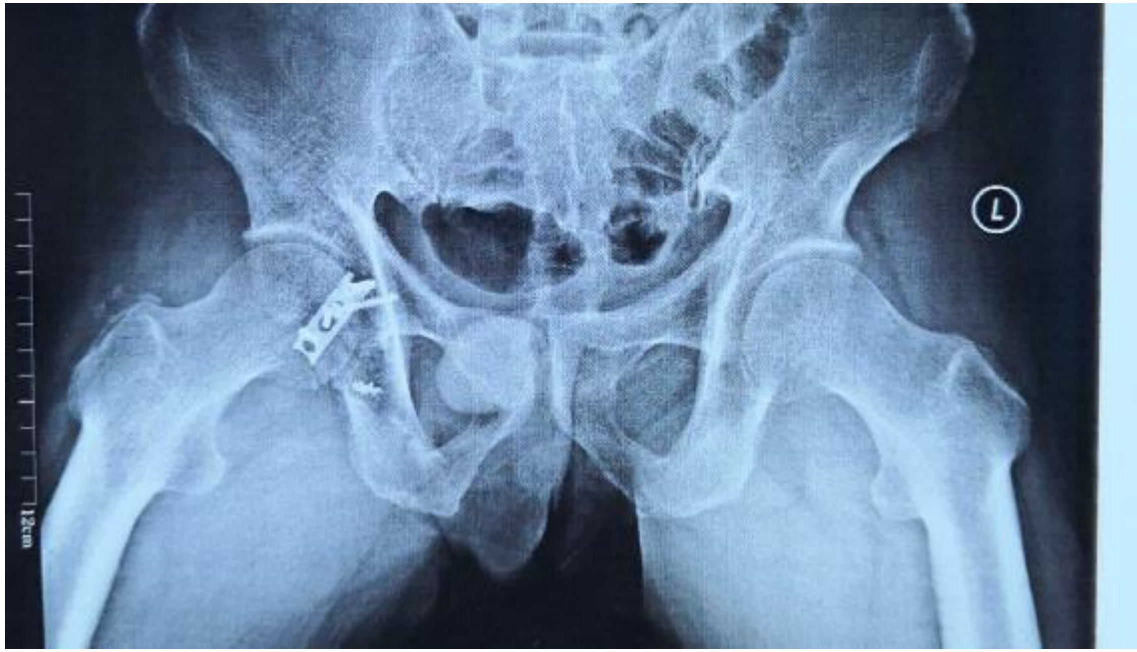

CASE 4

- -DAI, MALE, 52Y, Falling Injury, LEFT.

Preoperative

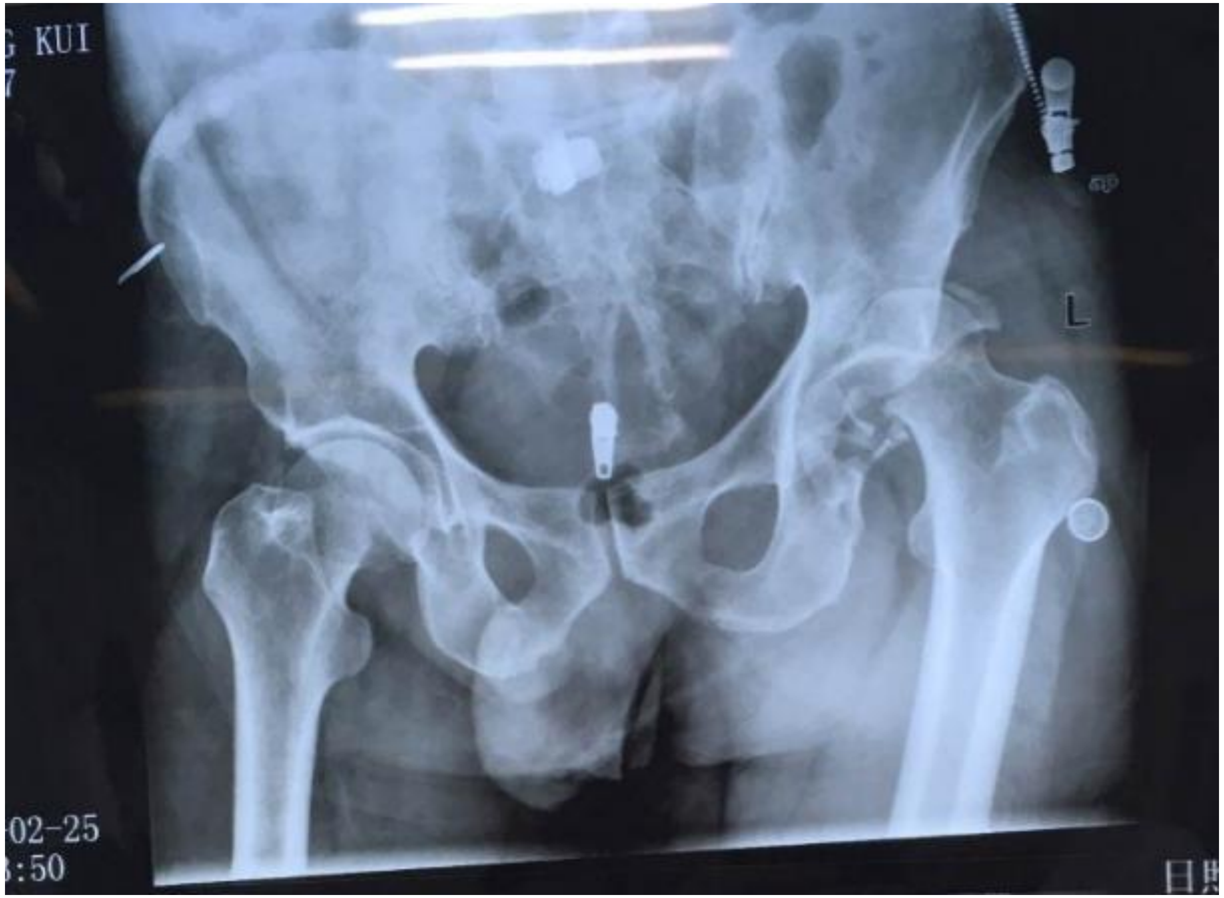

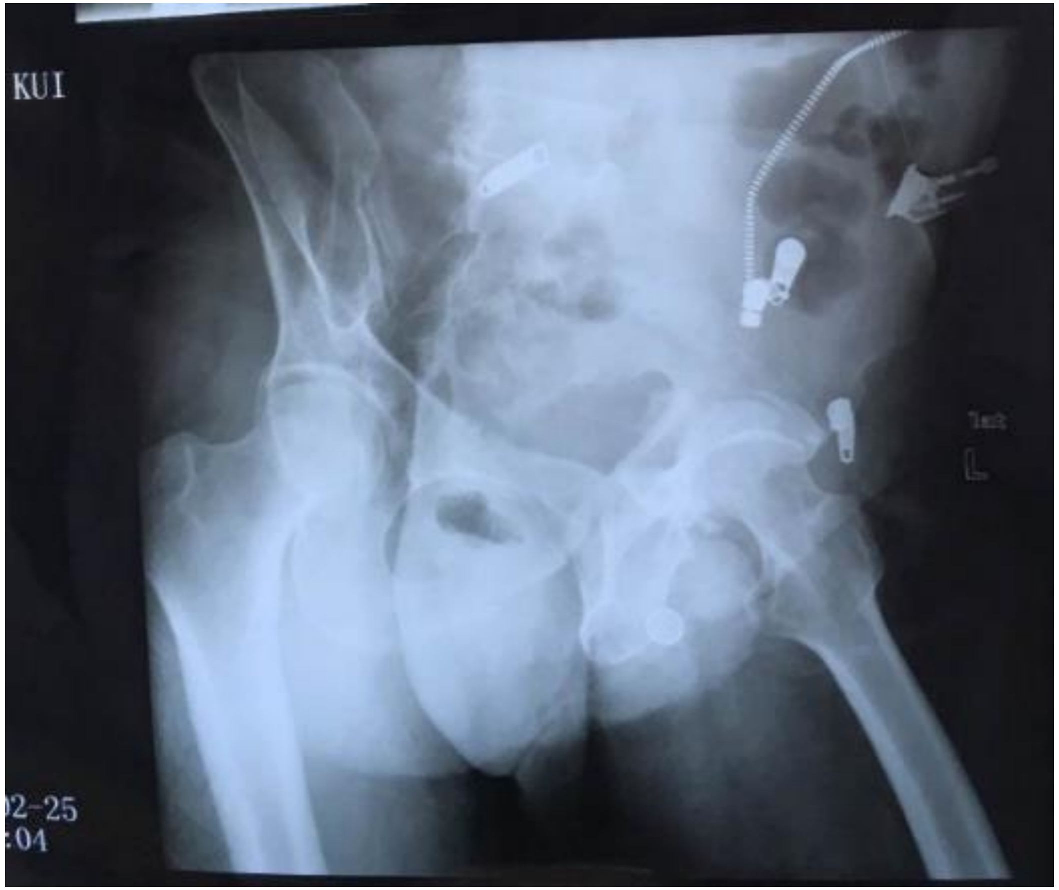

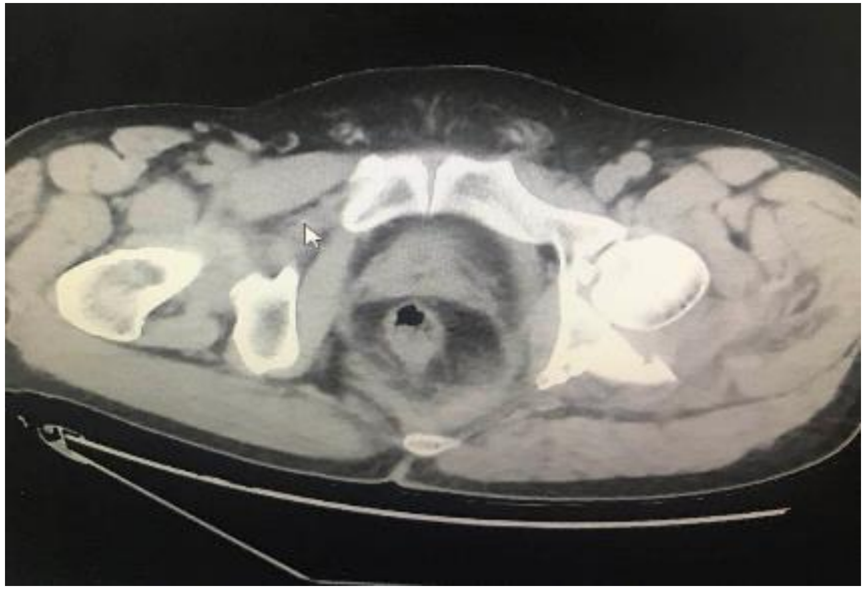

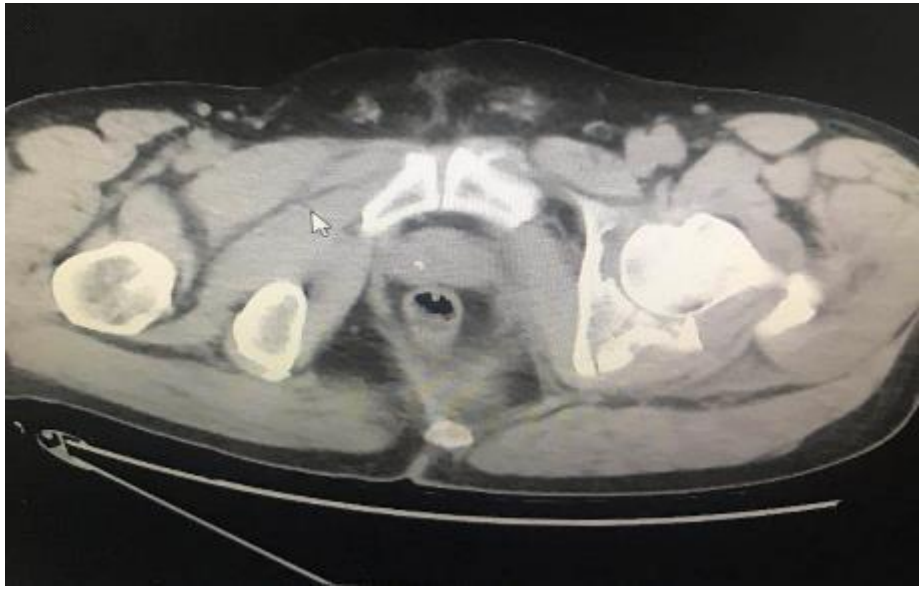

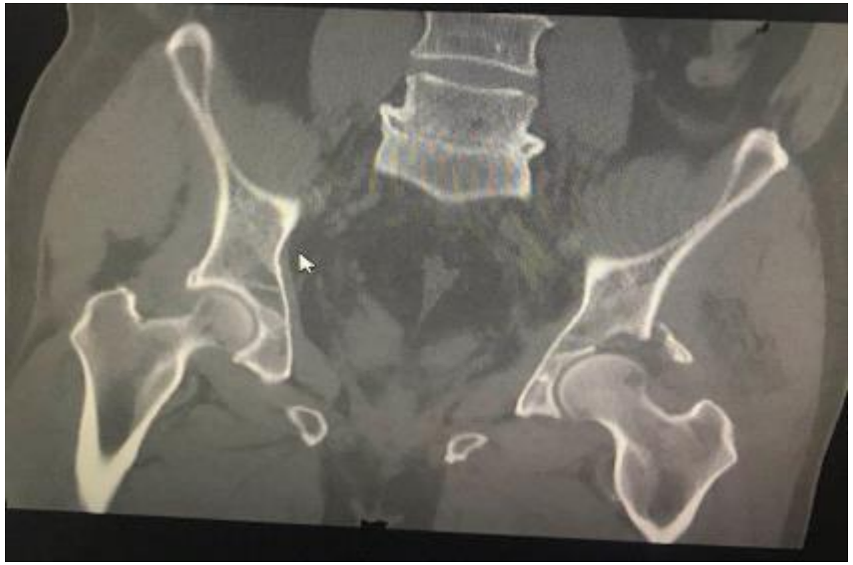

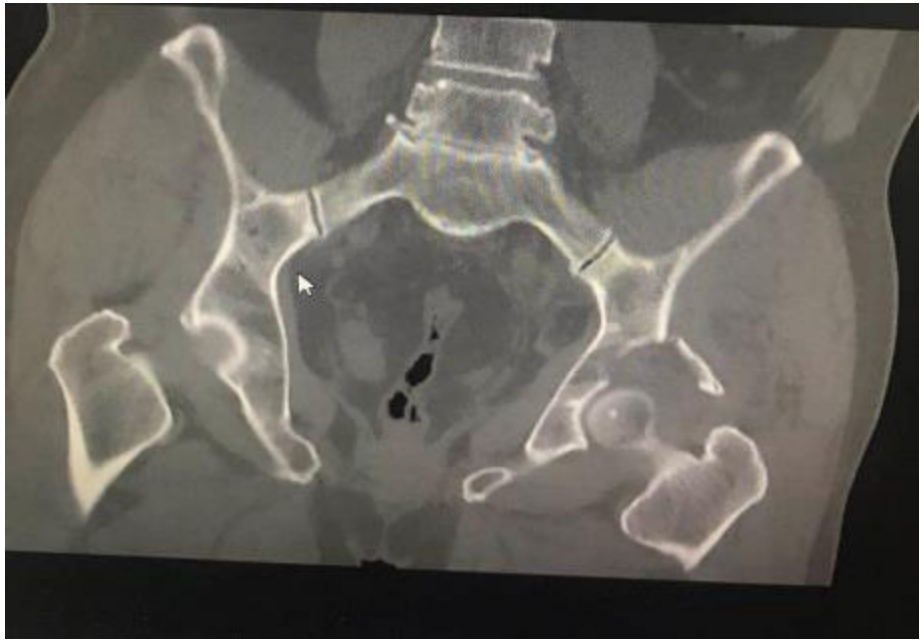

3D-CT, Preoperative

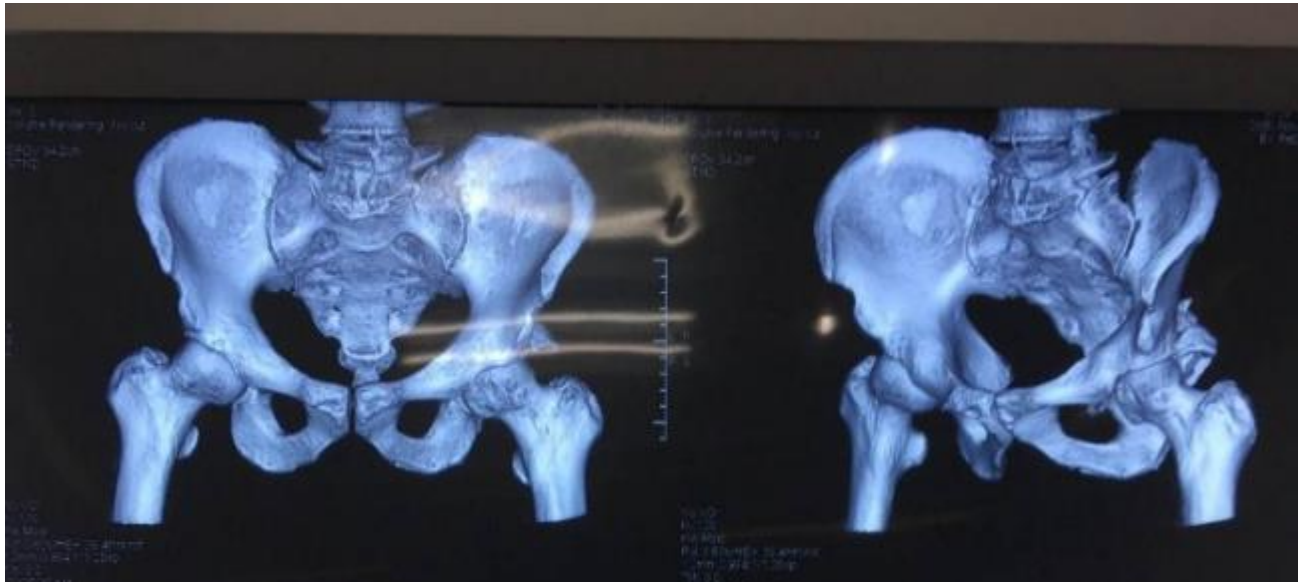

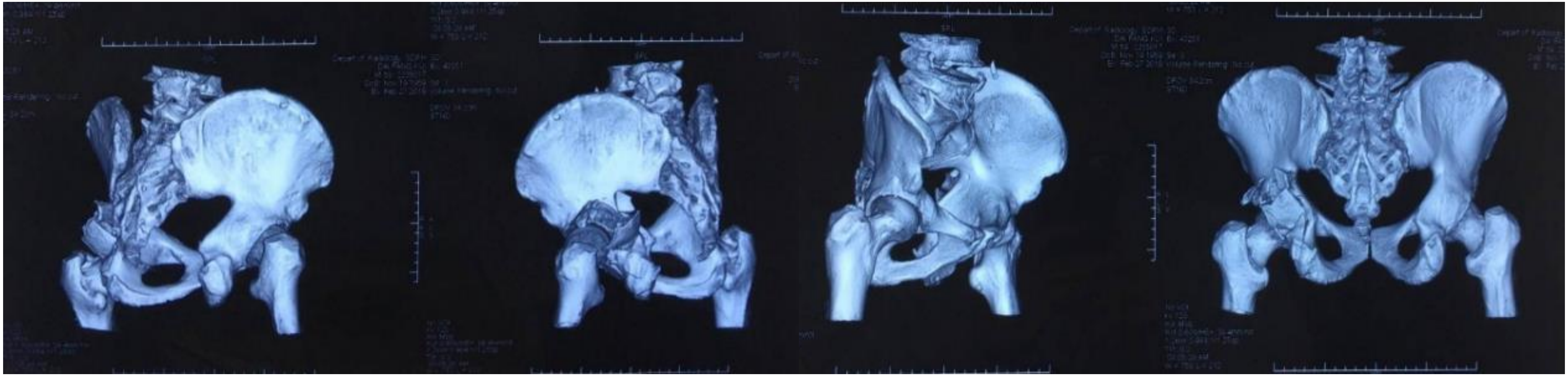

Intraoperative

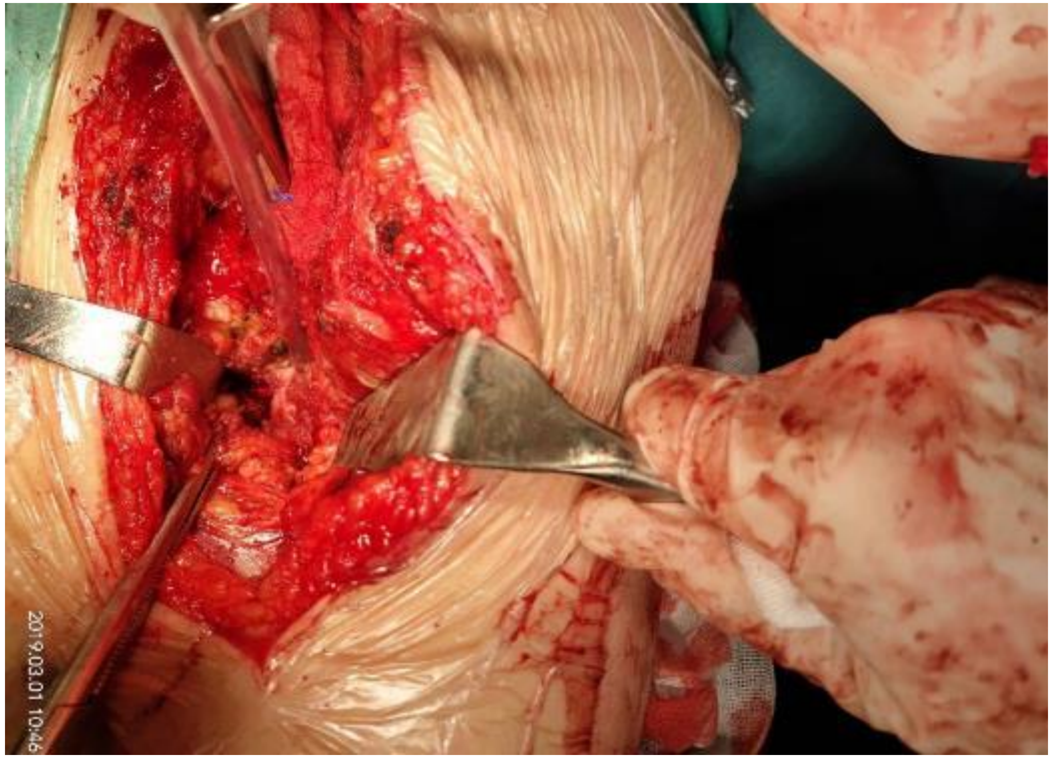

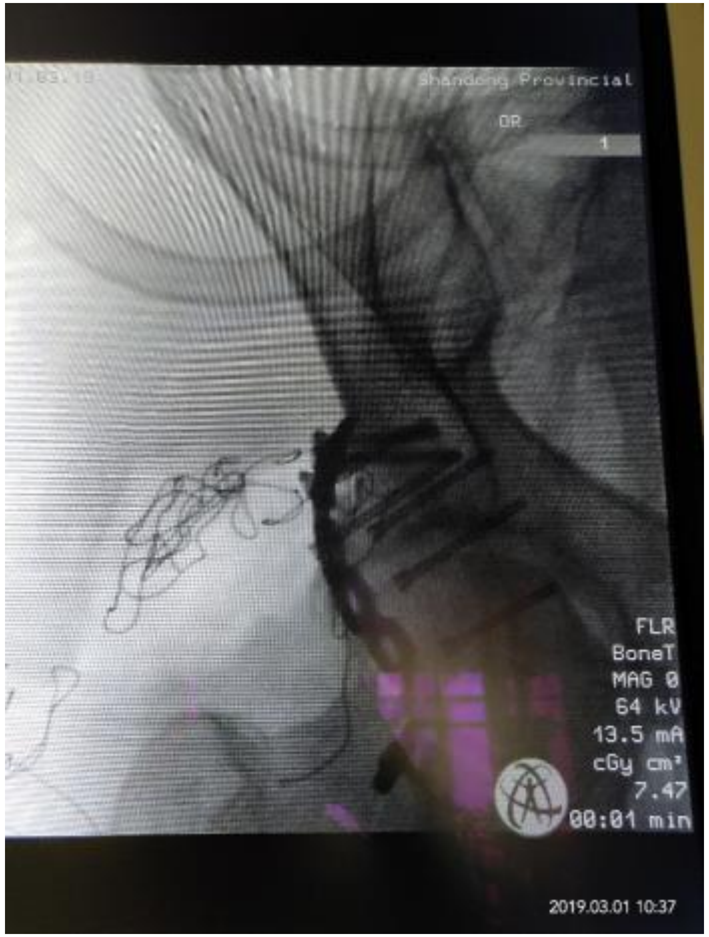

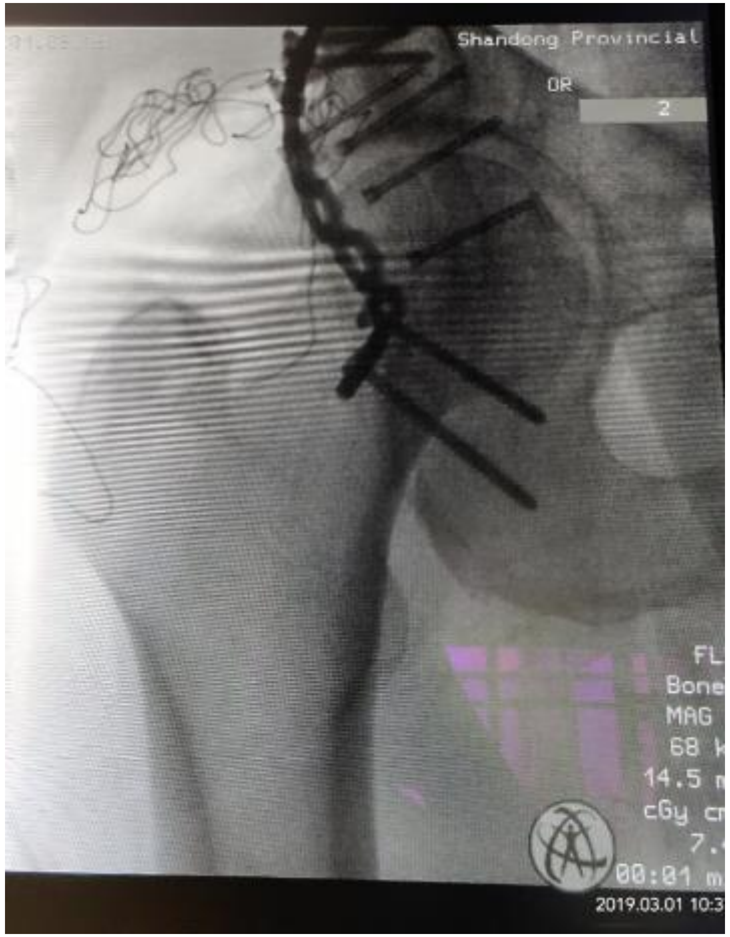

Postoperative

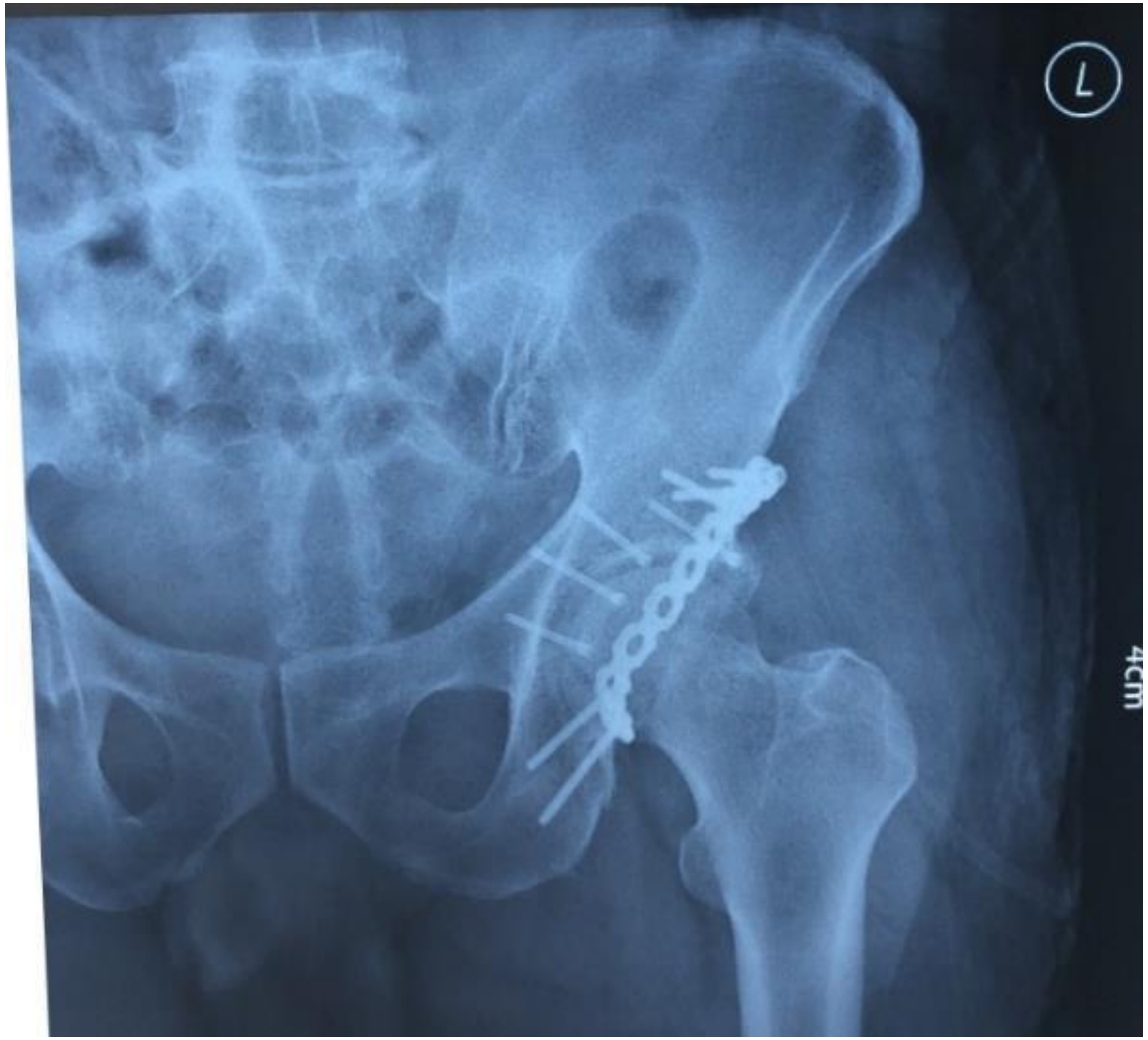

CASE 5

- -YU, MALE, 53Y, Falling Injury, LEFT.

Preoperative

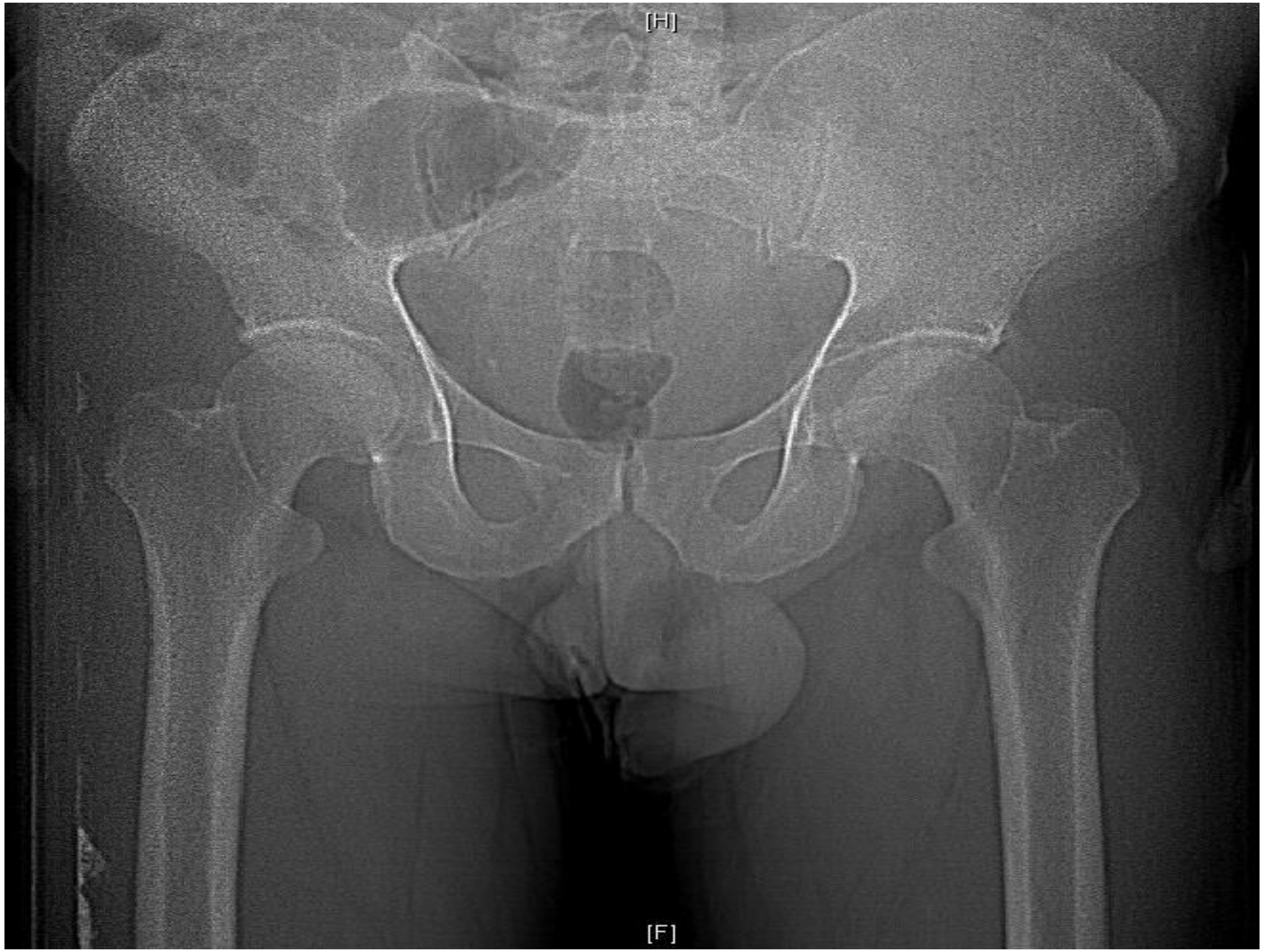

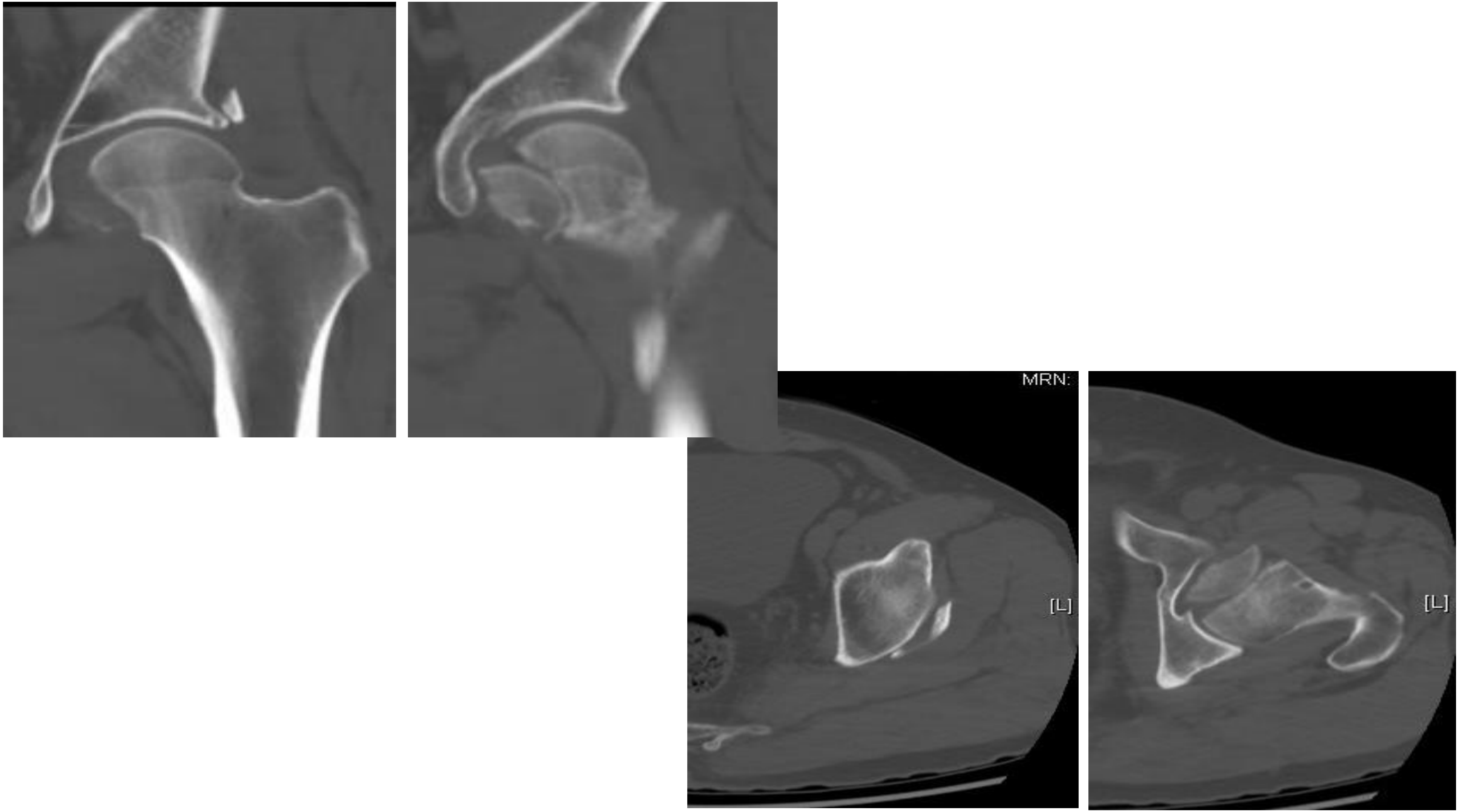
MRN:
[L]
[L]

Intraoperative

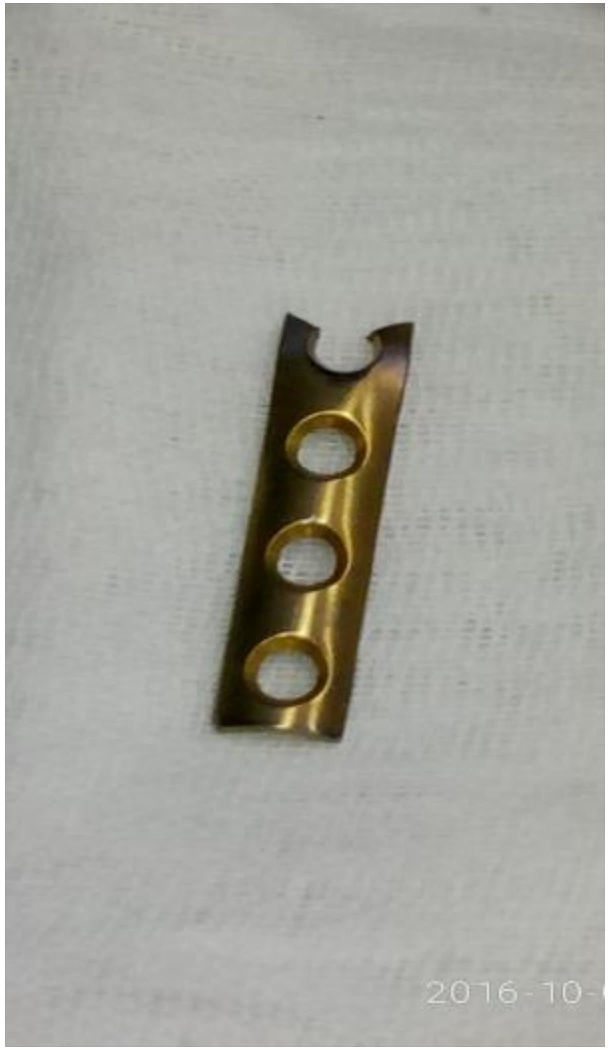

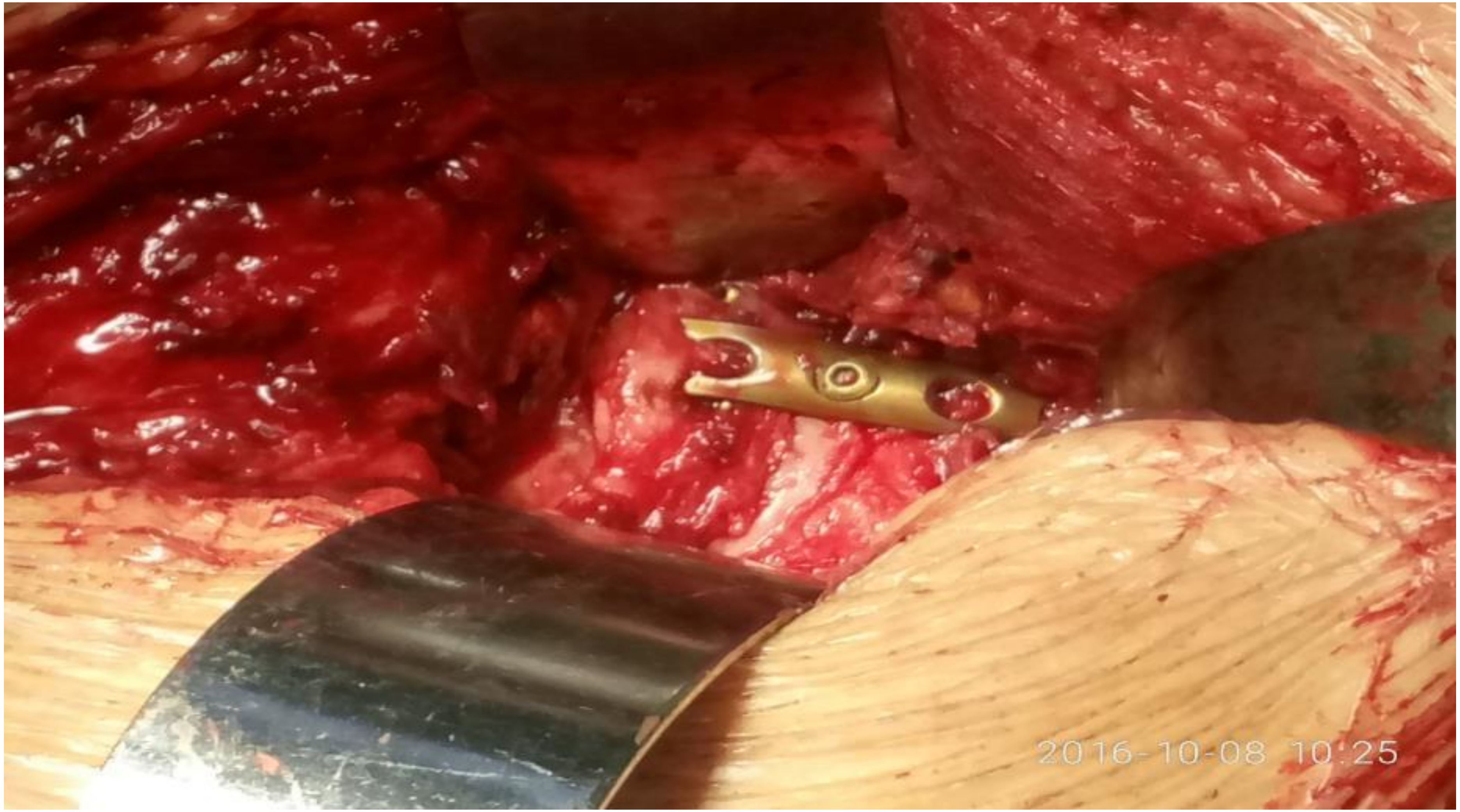

Postoperative

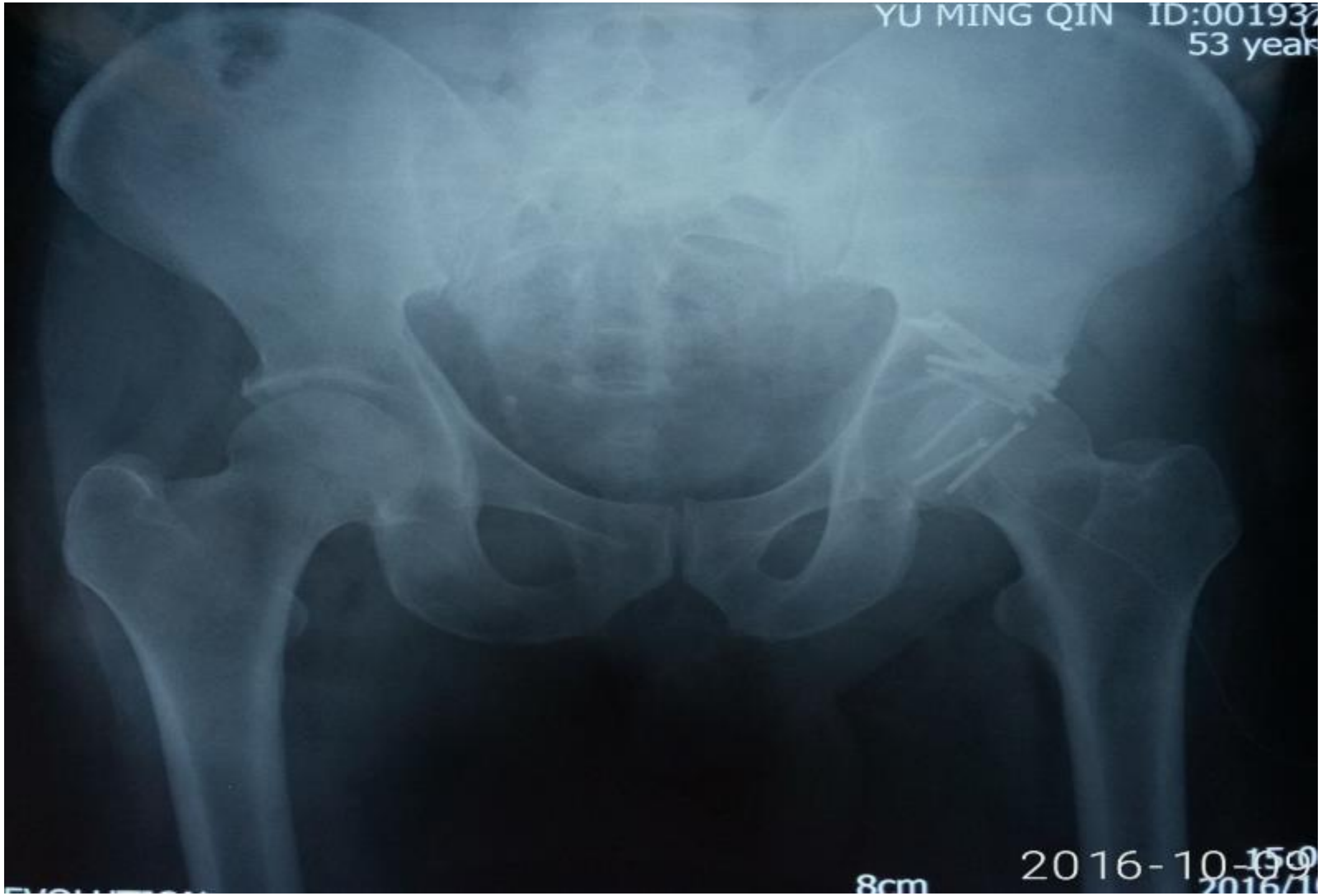

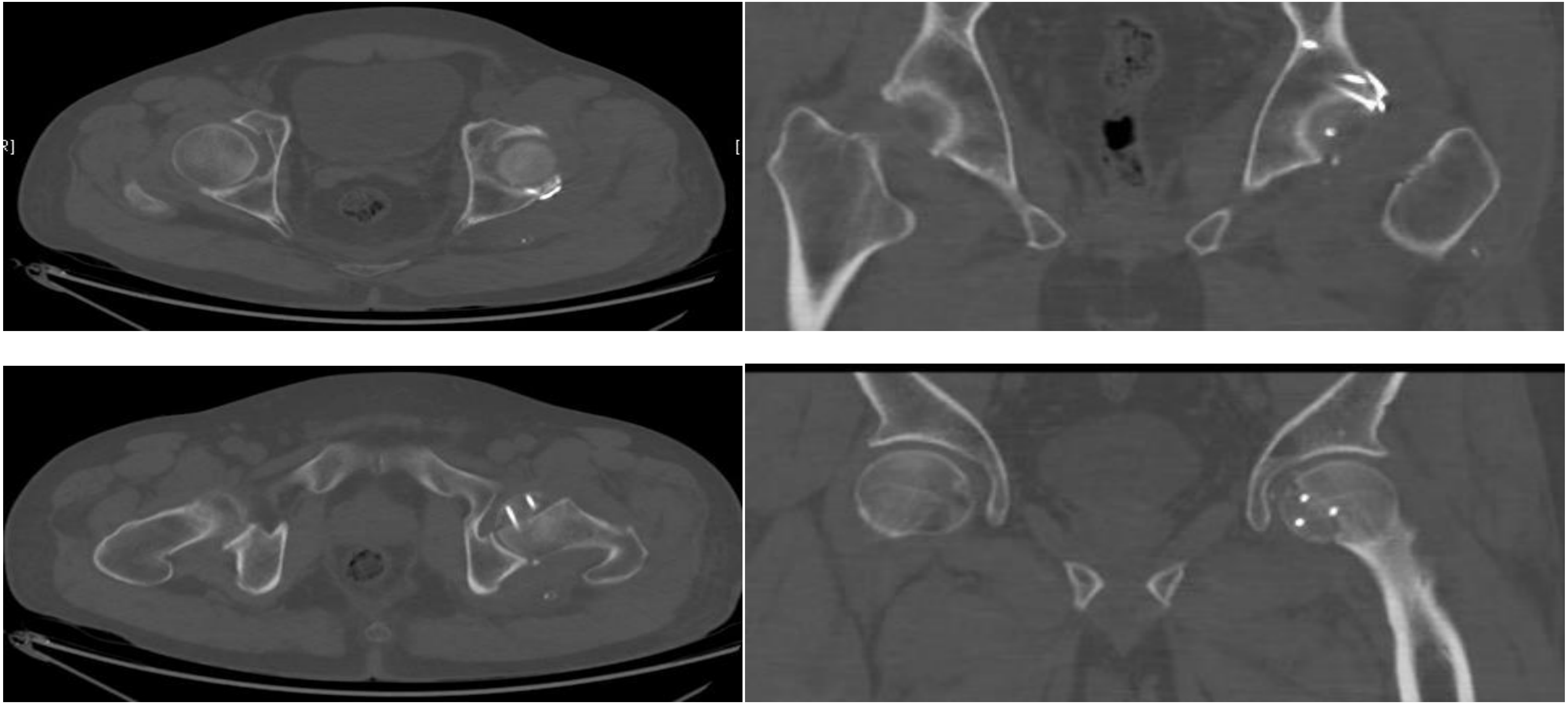

CASE 6

- -LIANG, MALE, 32Y, Traffic Accident Injury, RIGHT.

Preoperative

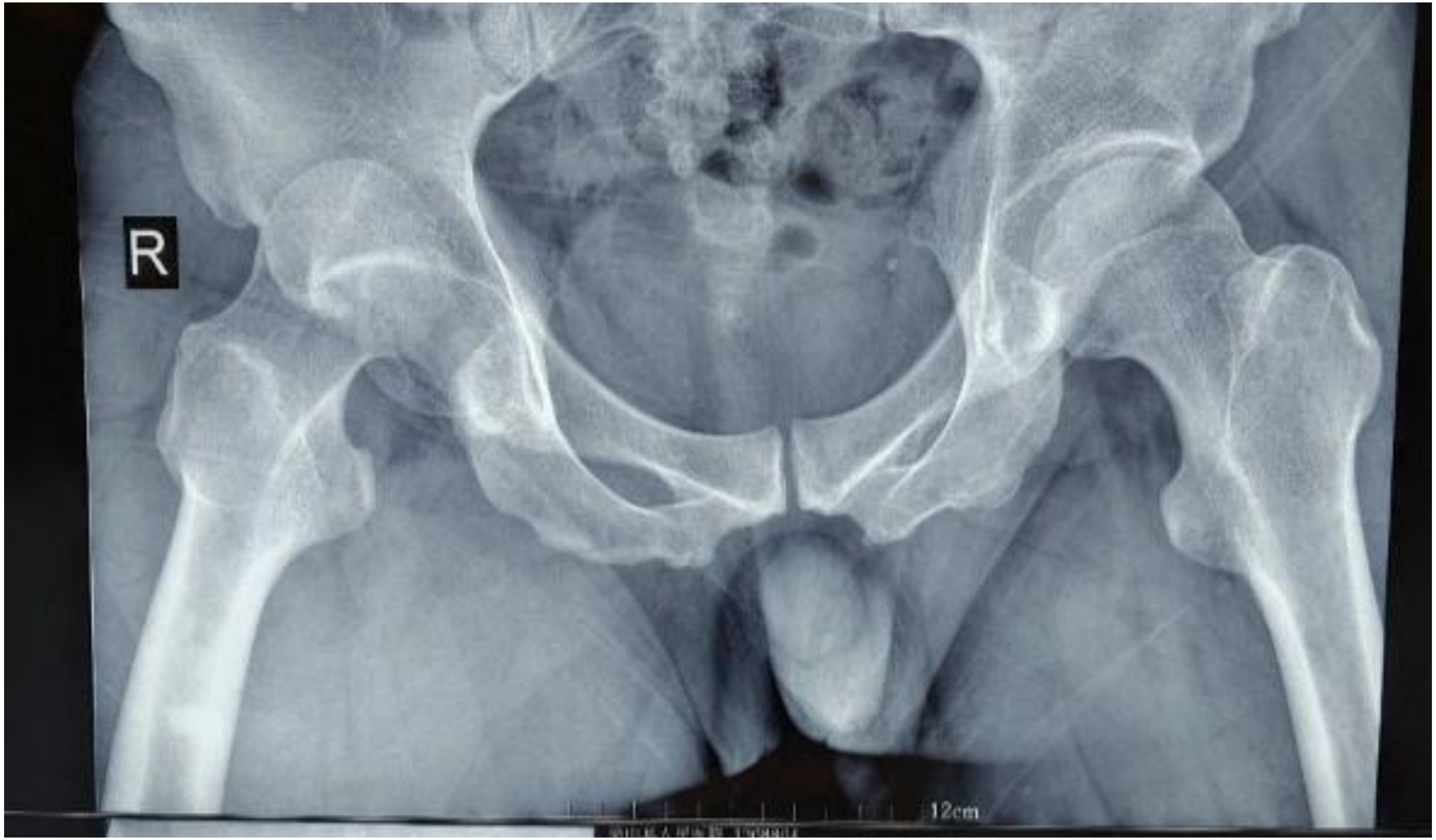

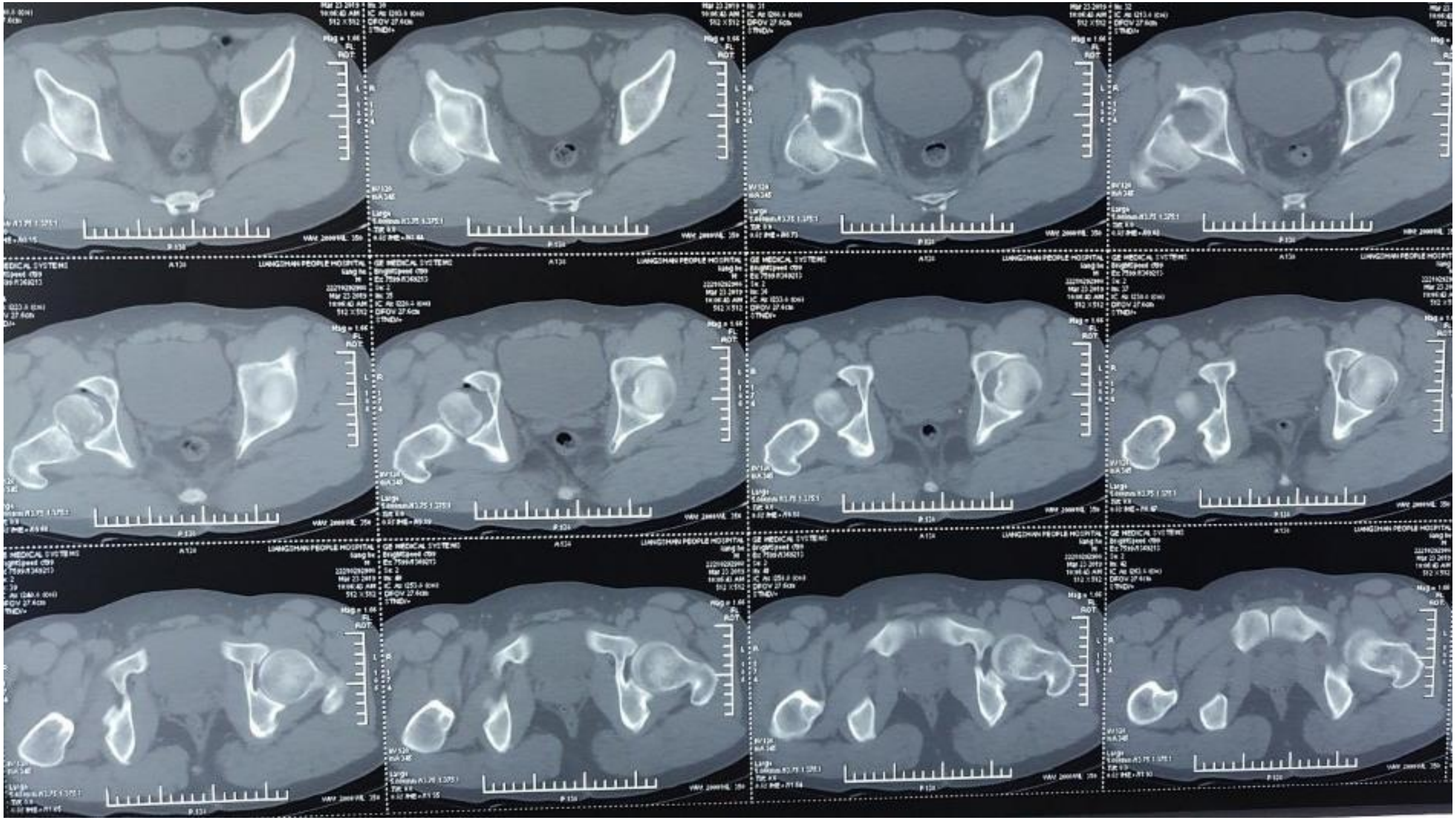

3D-CT, Preoperative

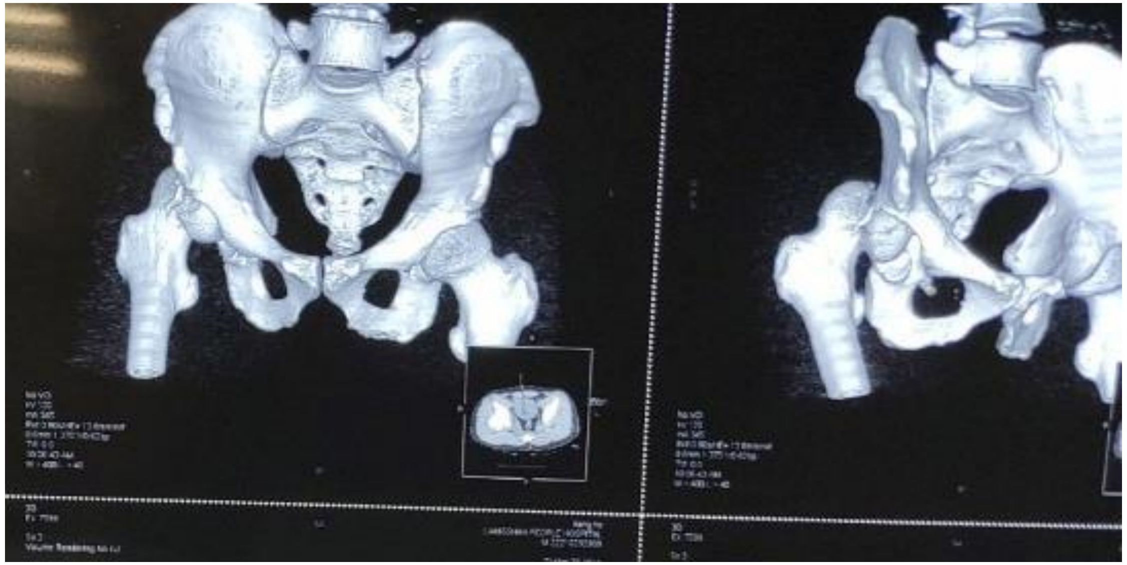

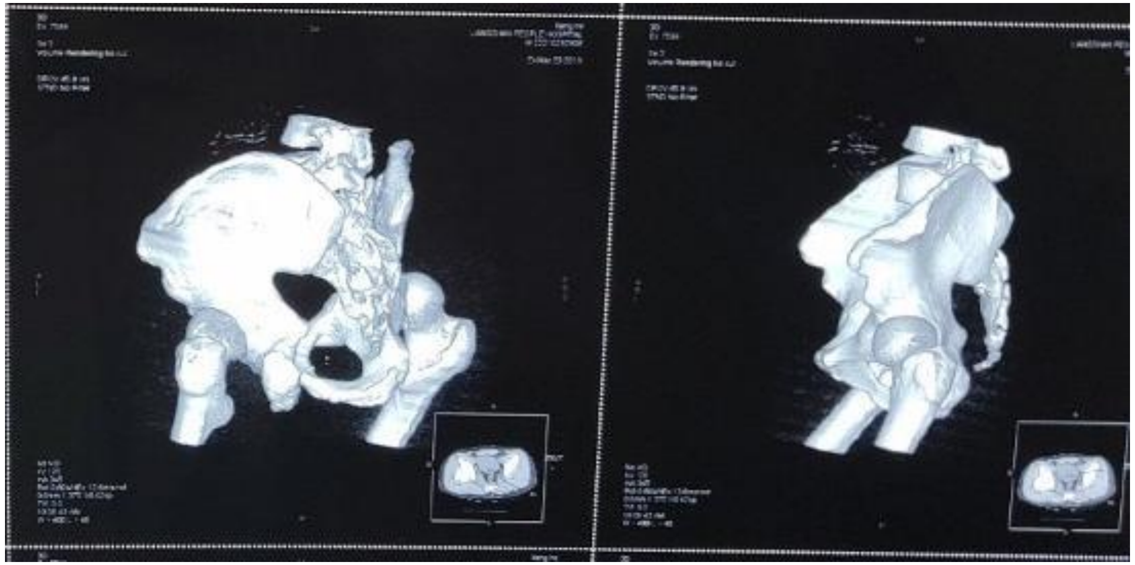

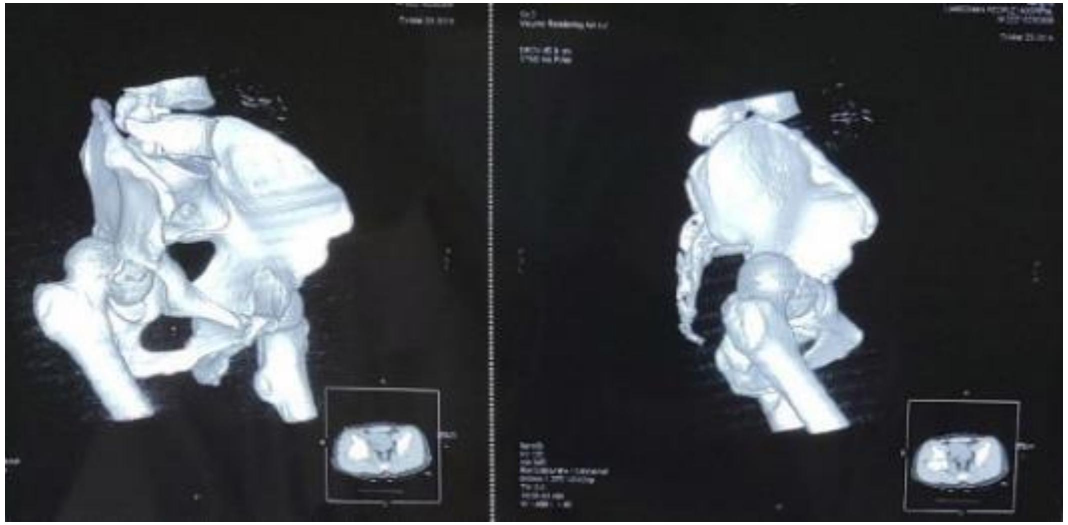

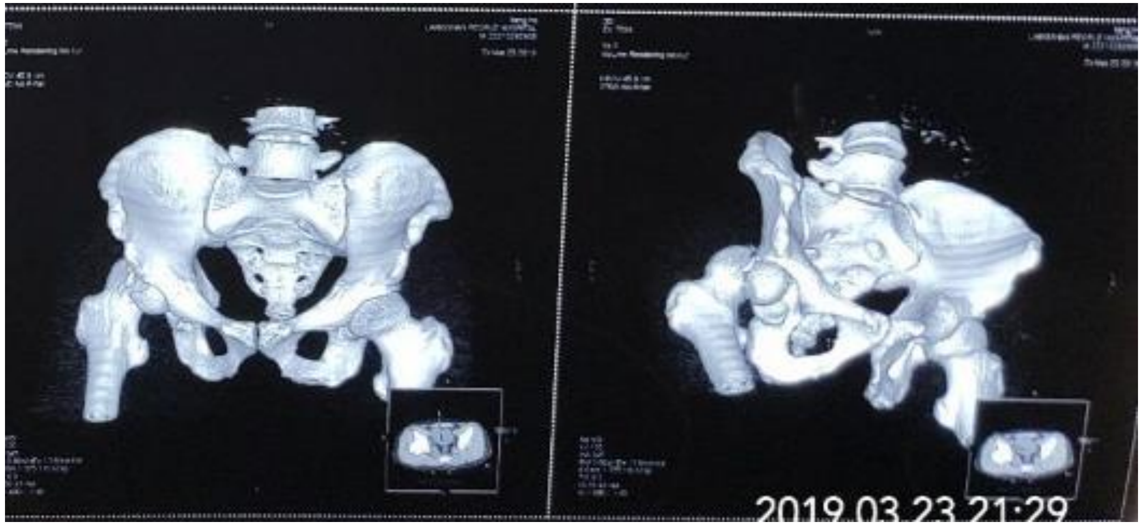

Postoperative

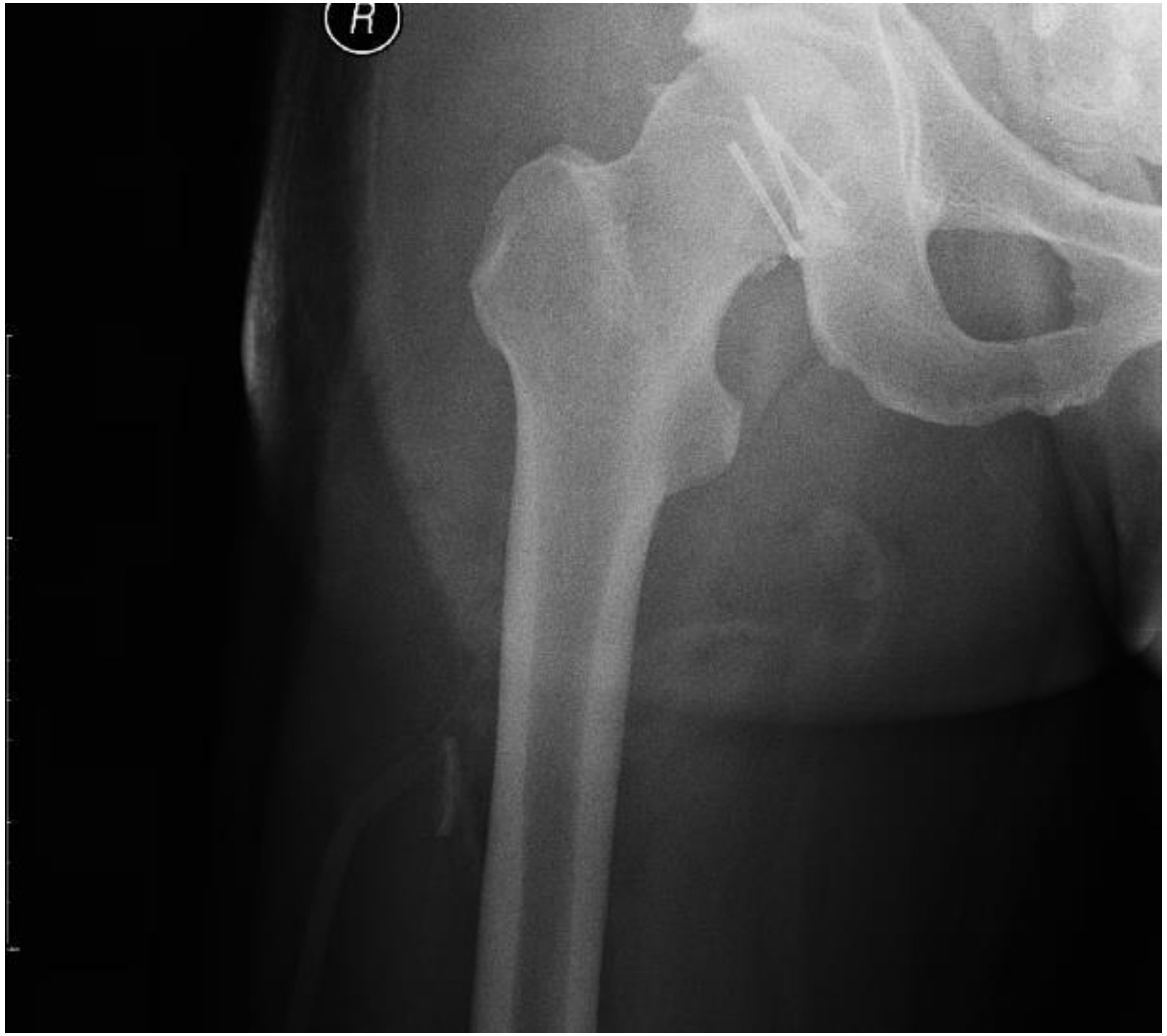

CASE 7

- -ZHANG, MALE, 30Y, Falling Injury, LEFT.

Preoperative

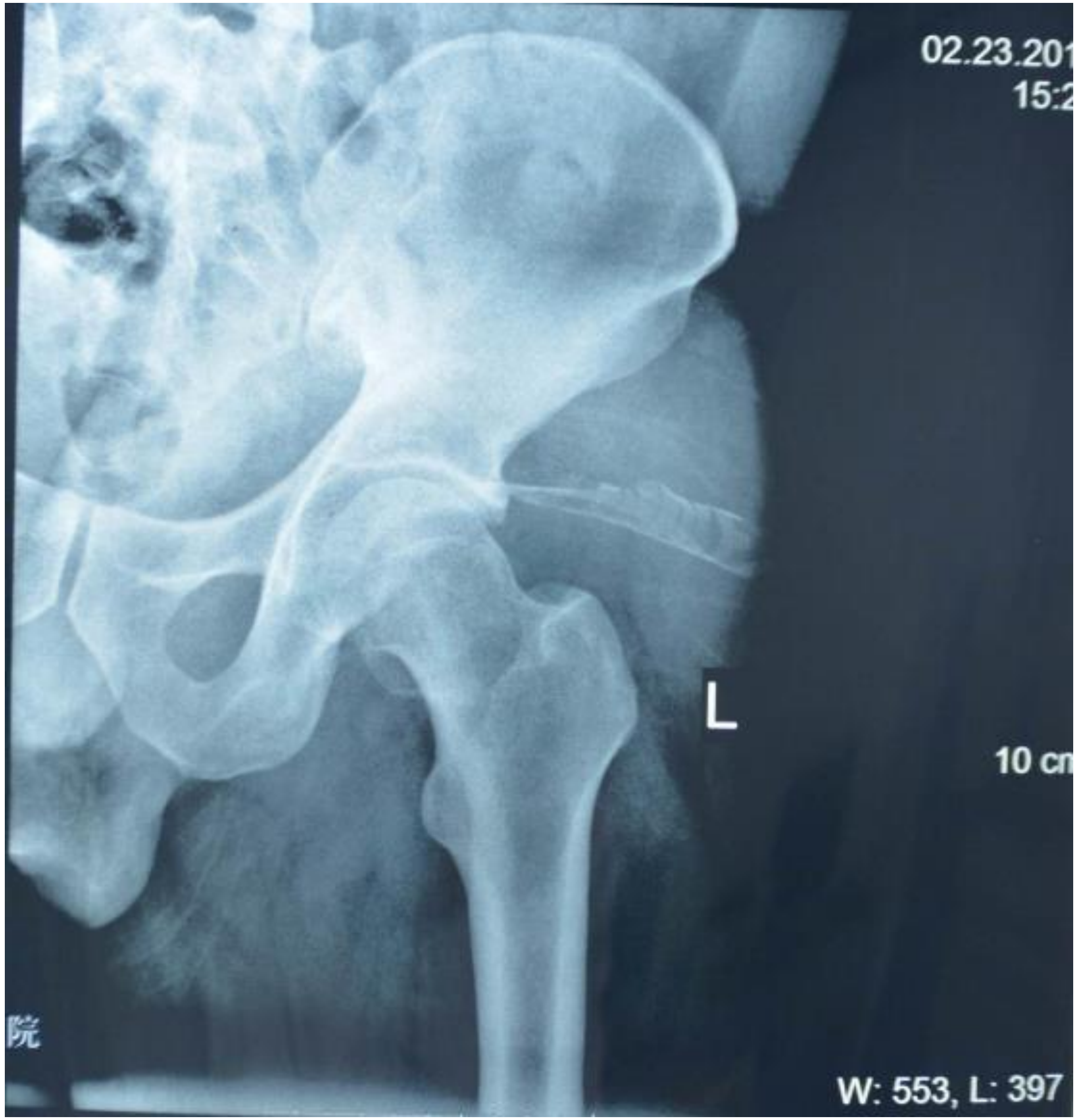

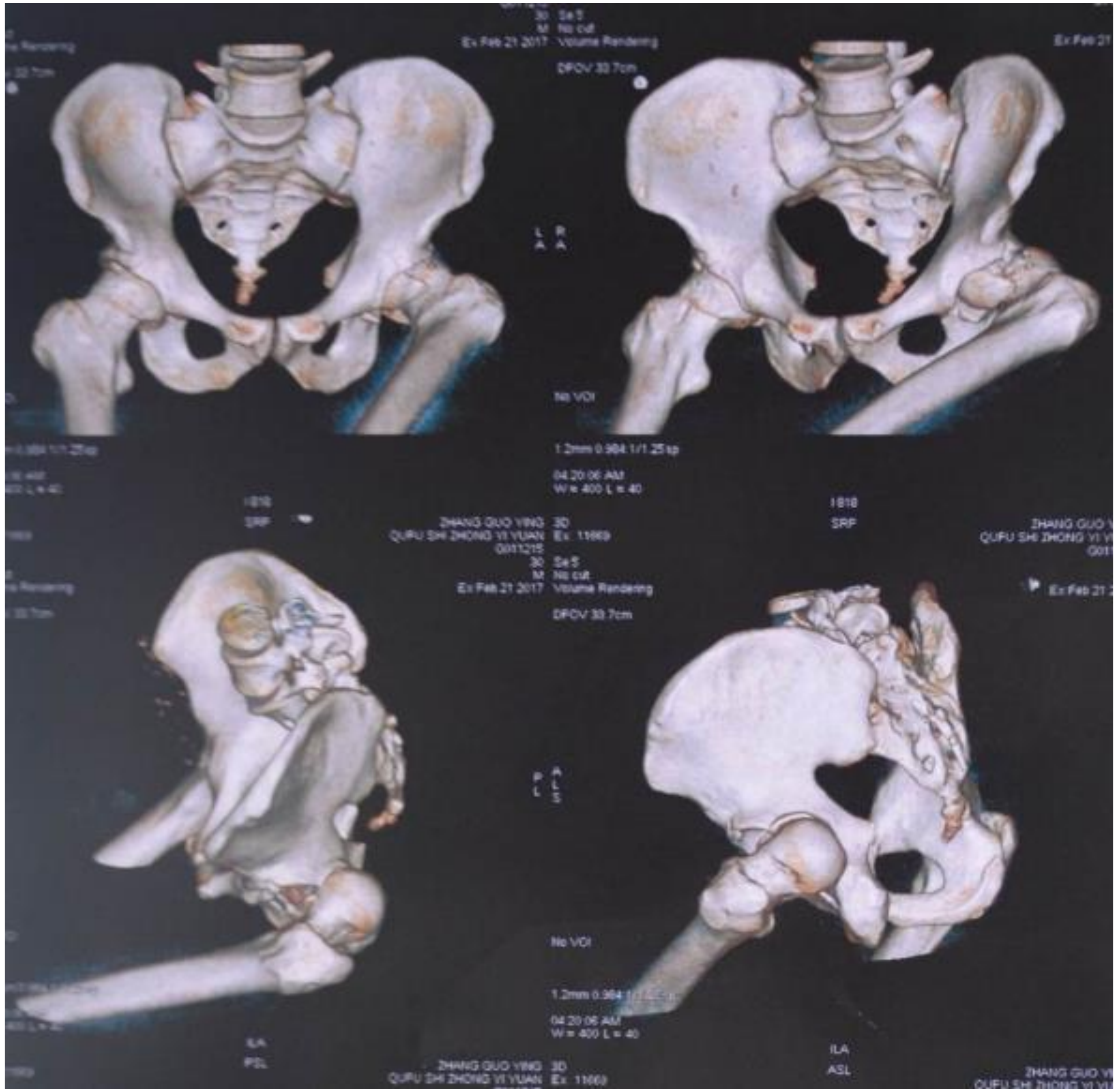

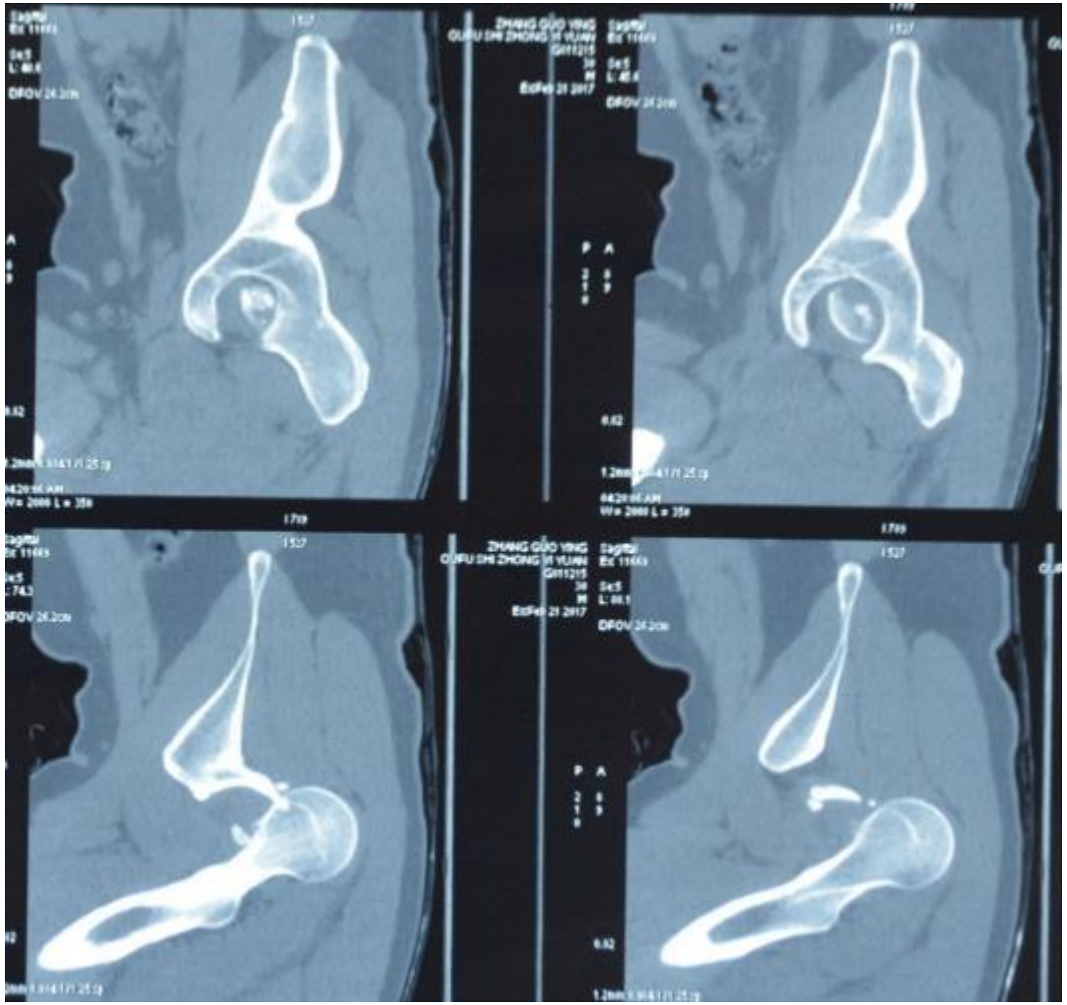

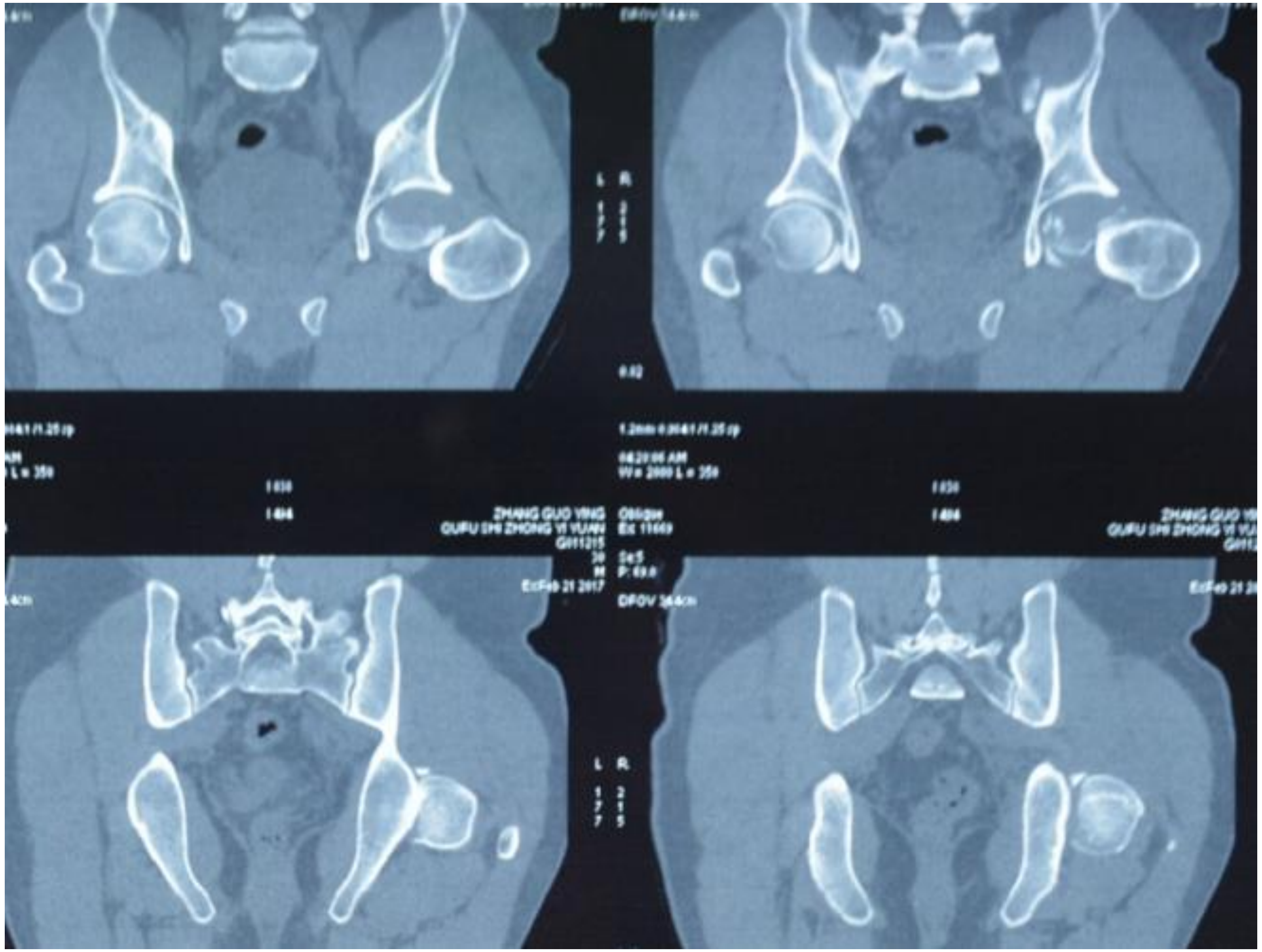

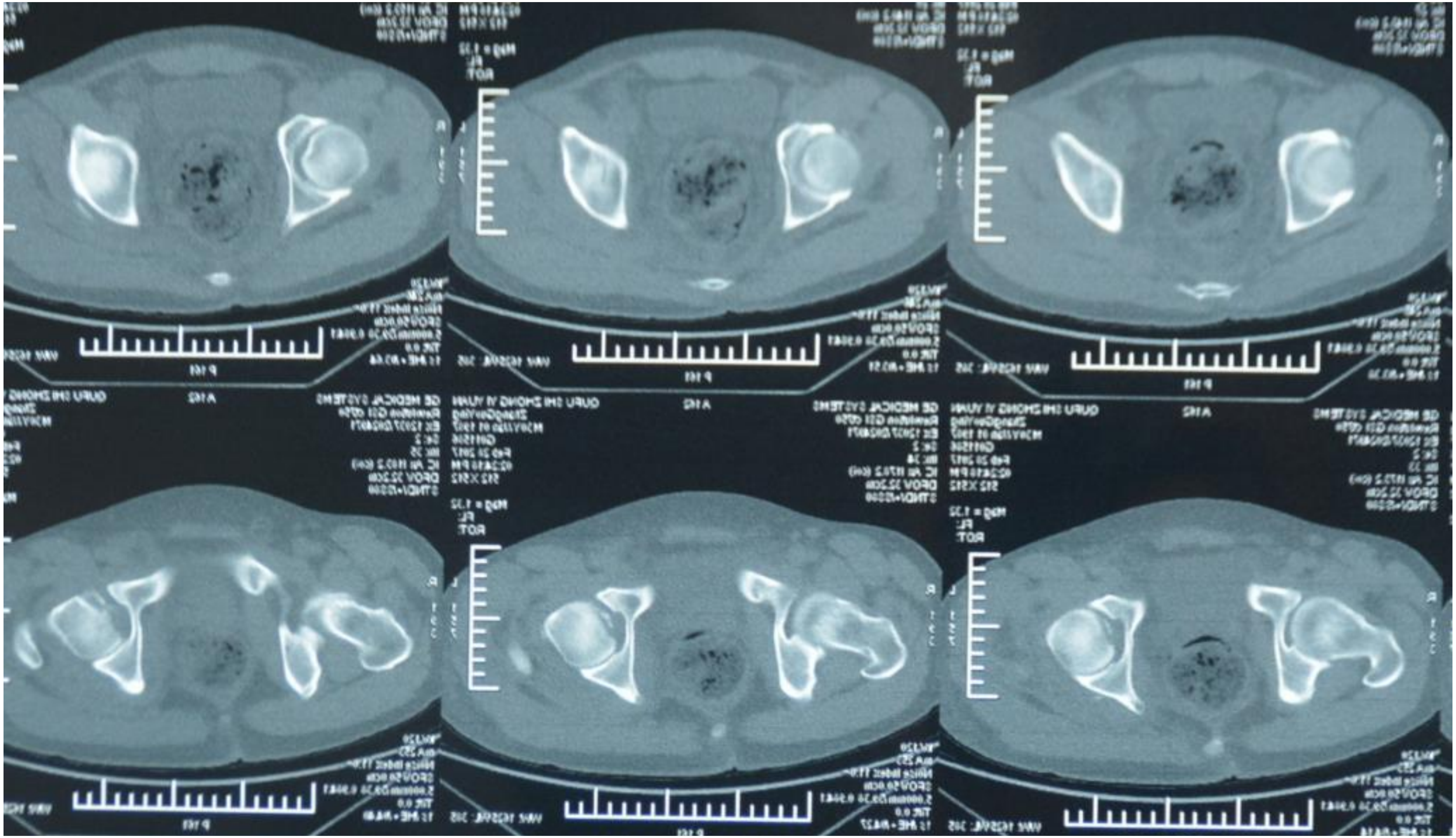

Intraoperative

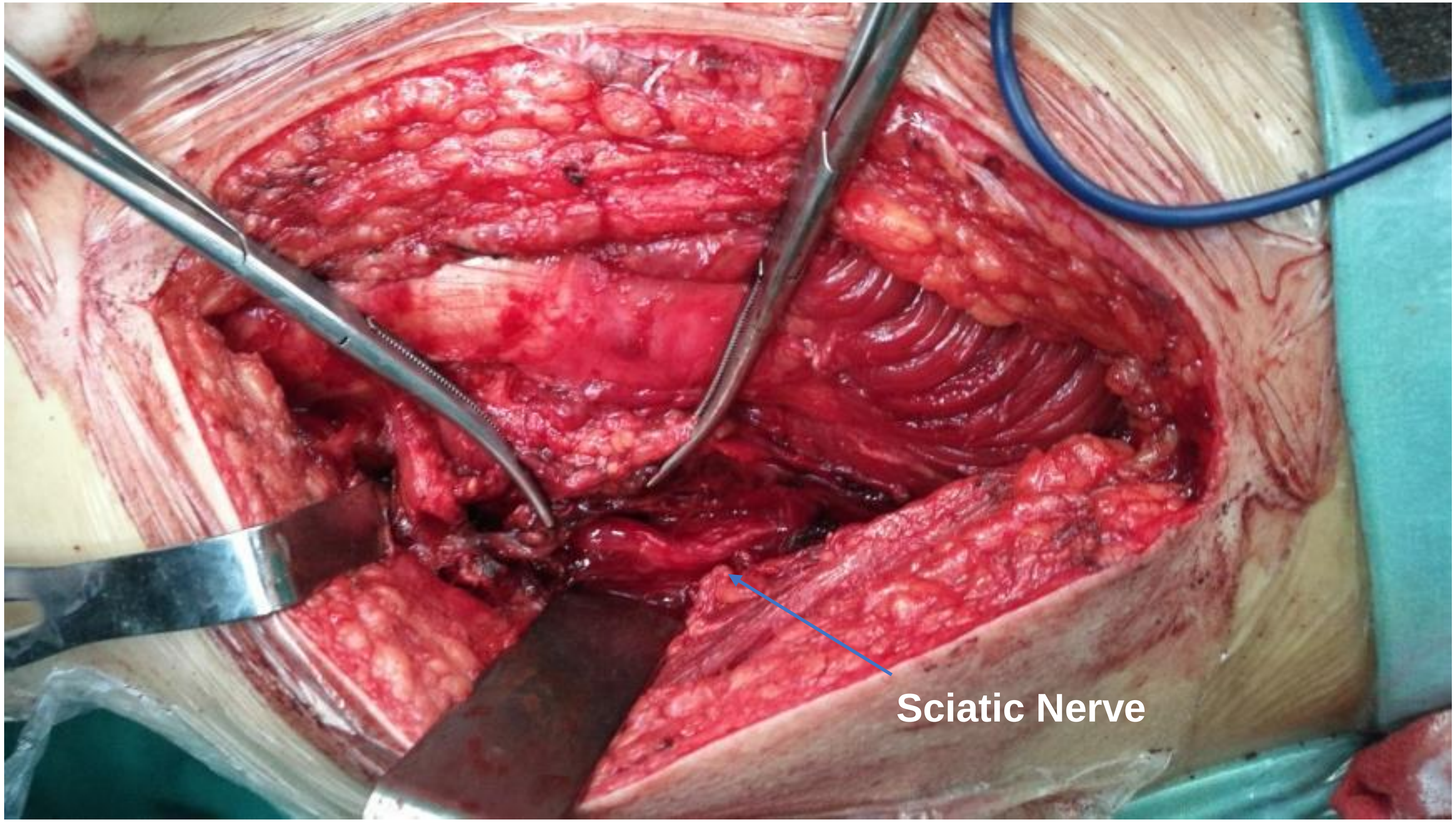

Postoperative

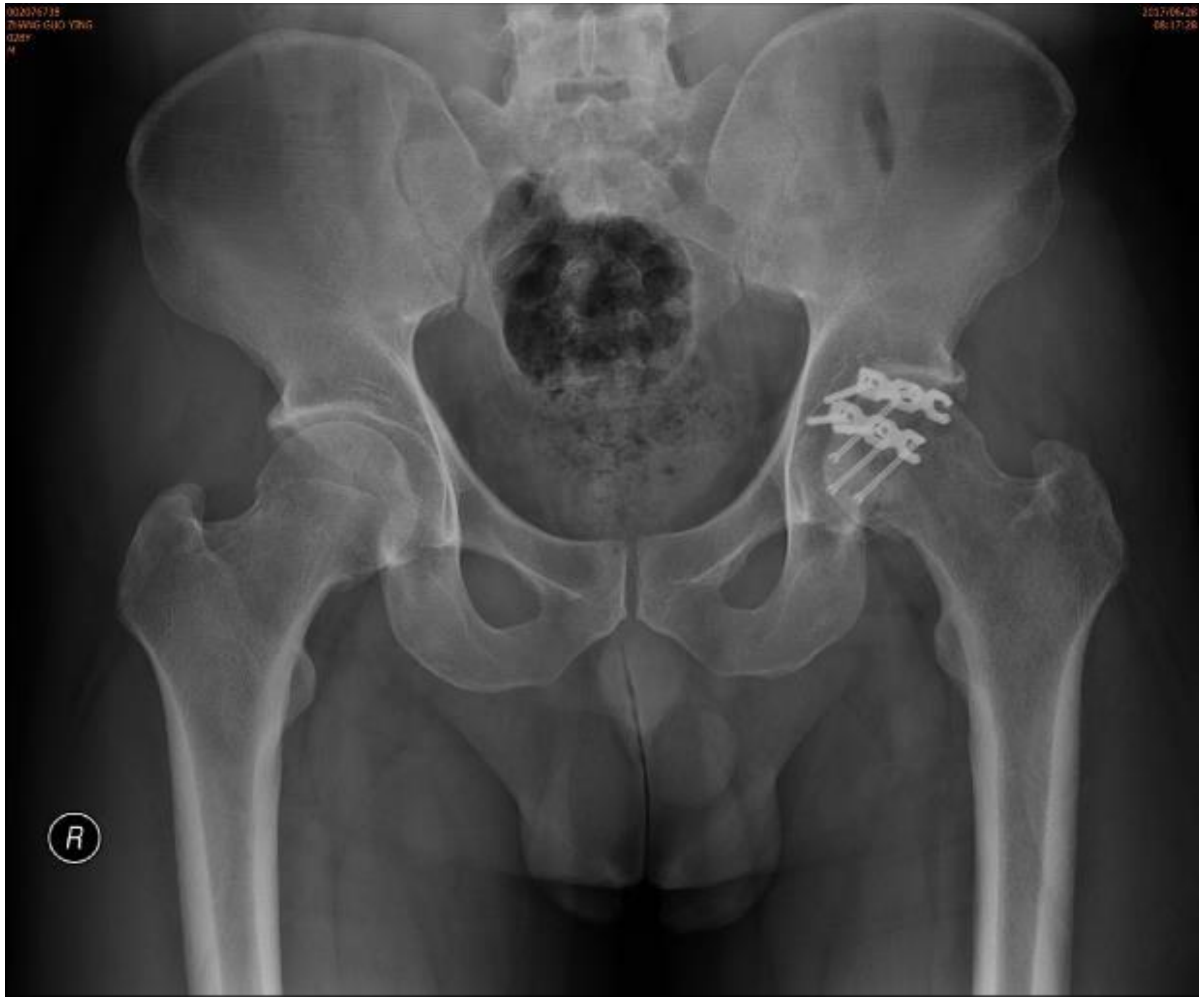

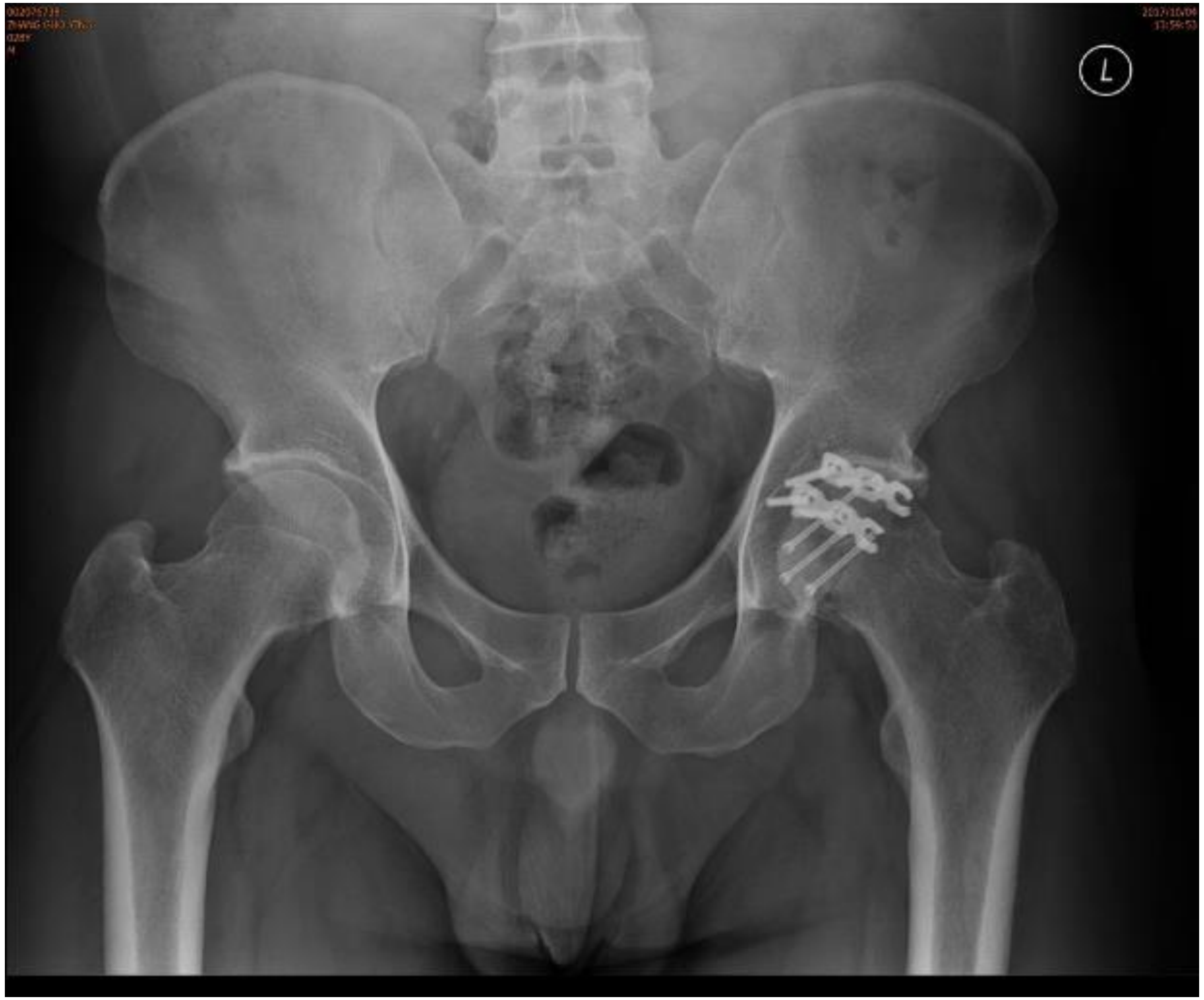

CASE 8

- -LI, MALE, 24Y, Falling Injury, RIGHT.

Preoperative

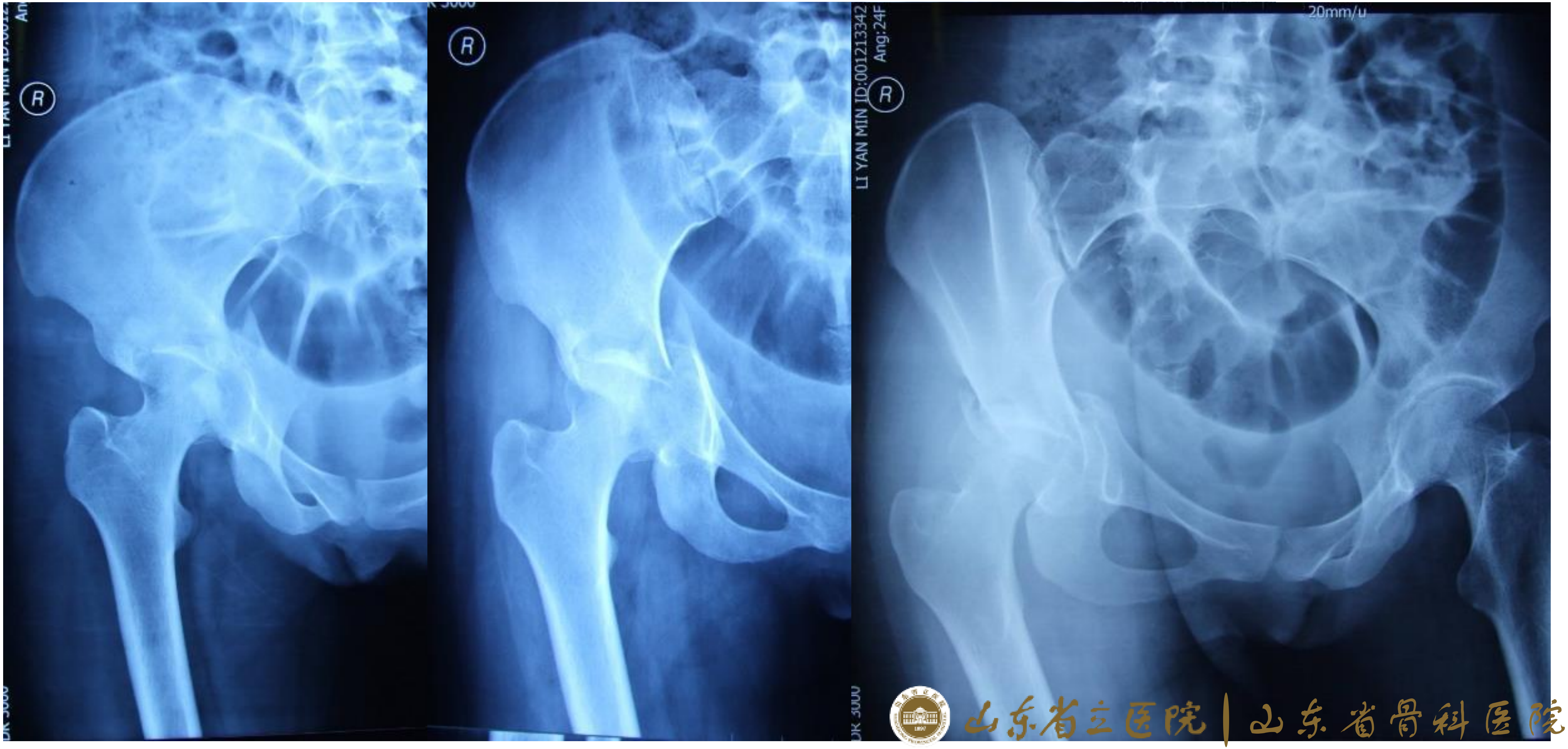

3D-CT, Preoperative

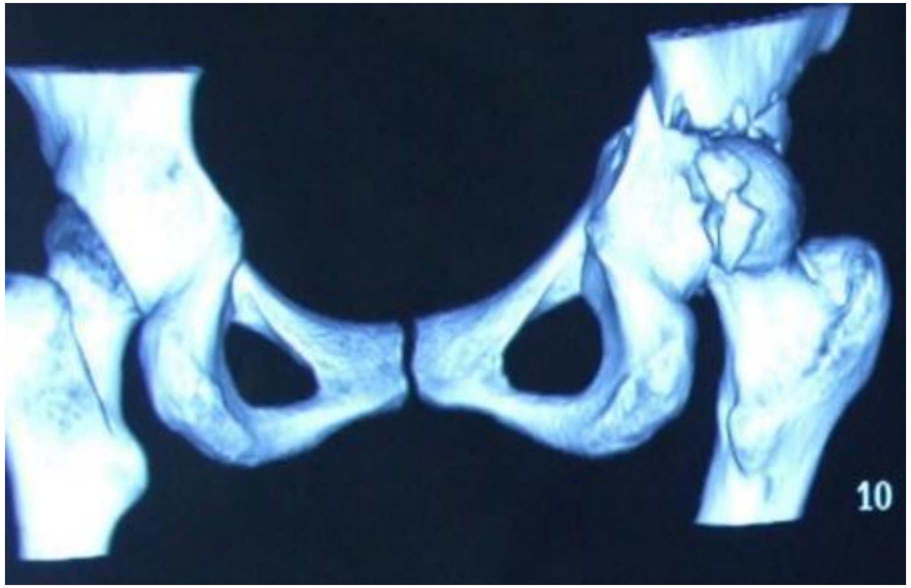

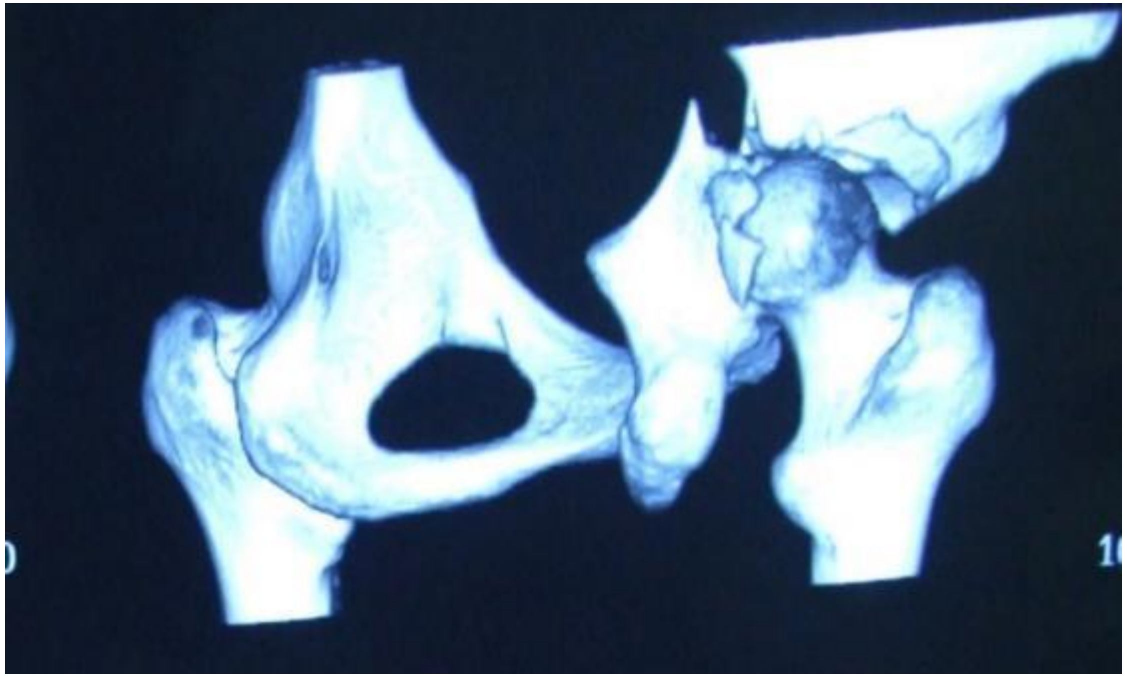

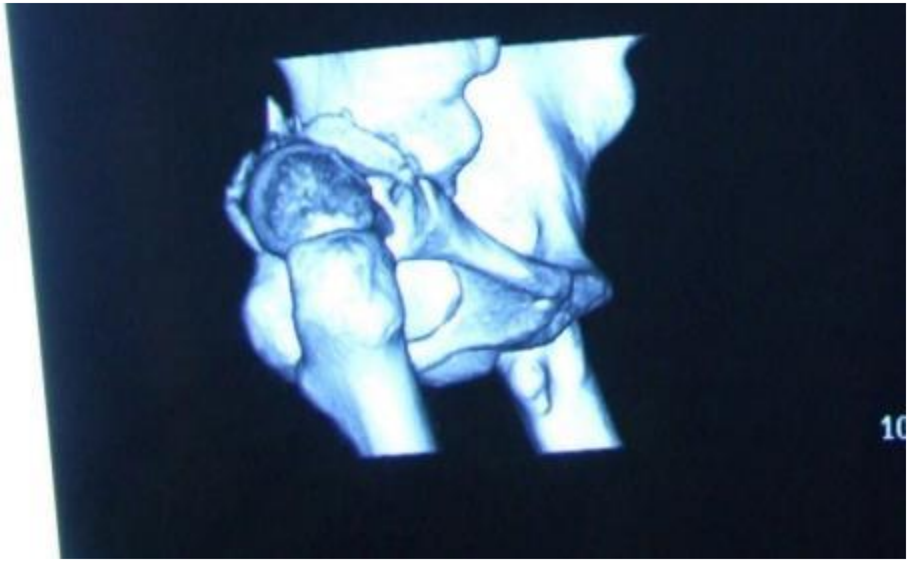

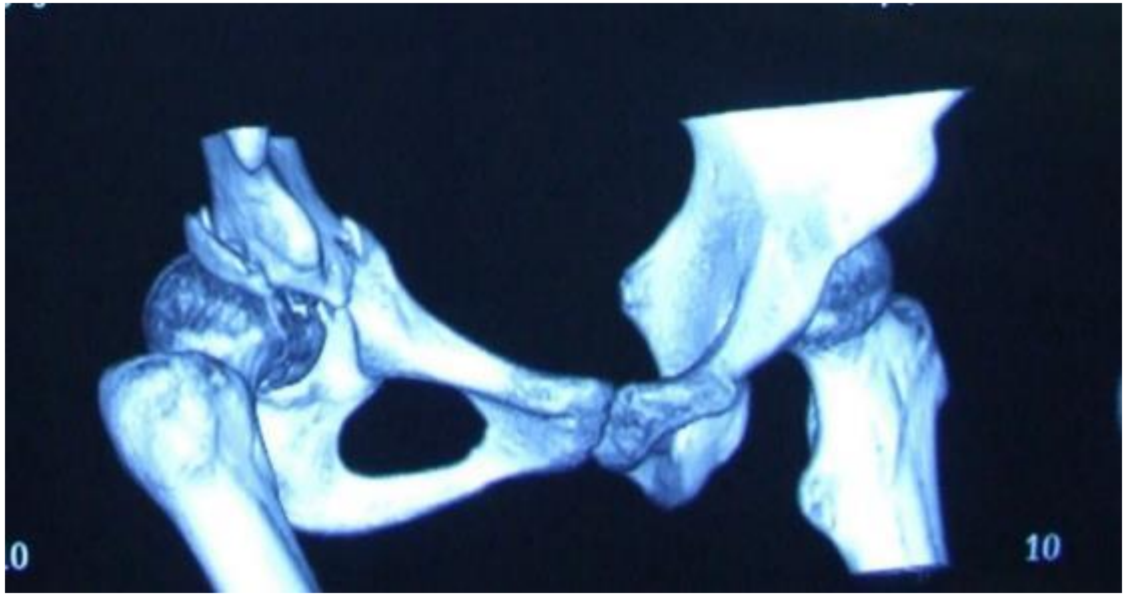

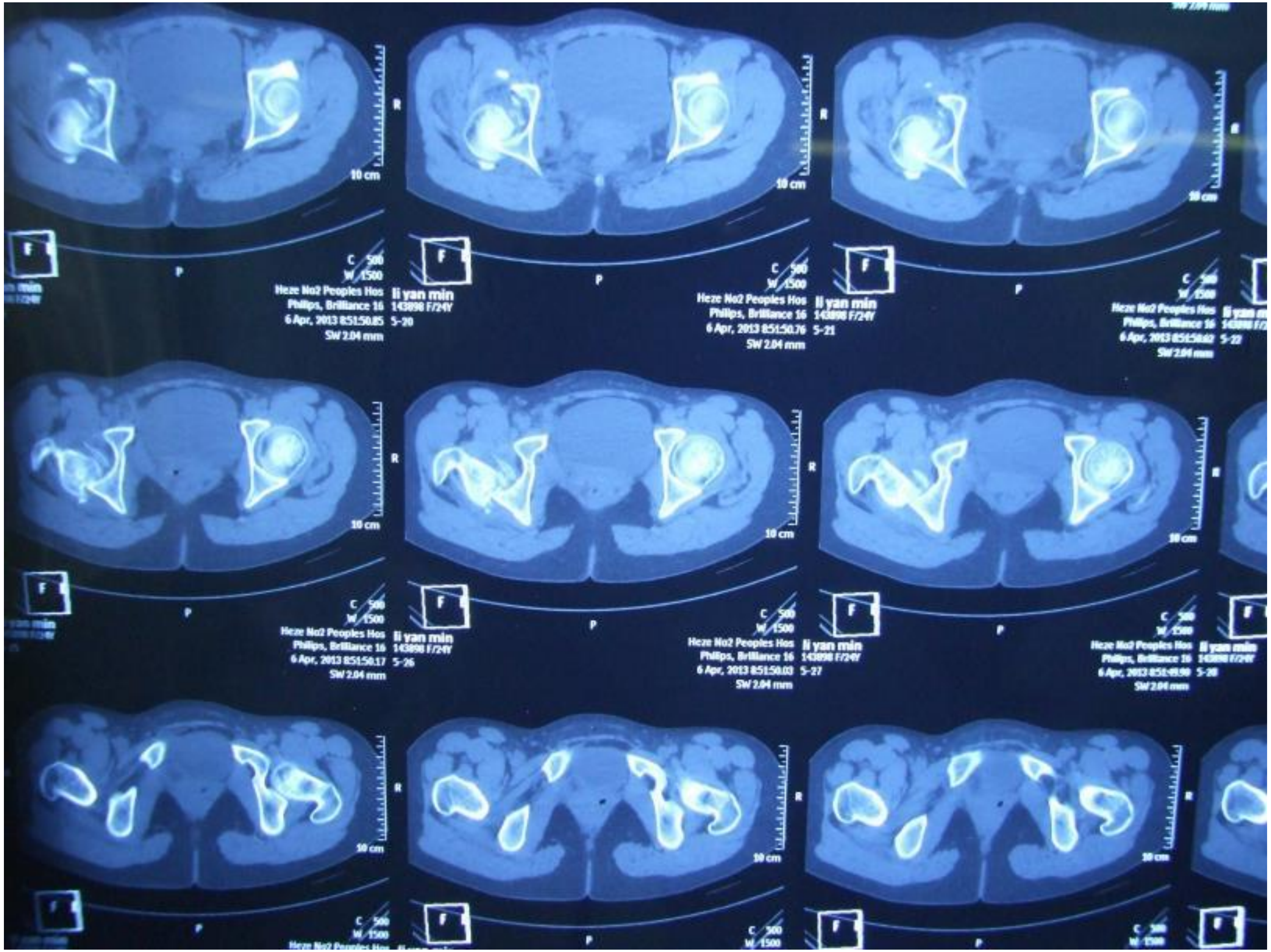
10 cm
R
P
C 500
W 1500
Heze No2 Peoples Hos
li yan min
Philips, Brilliance 16
SW 2.04 mm

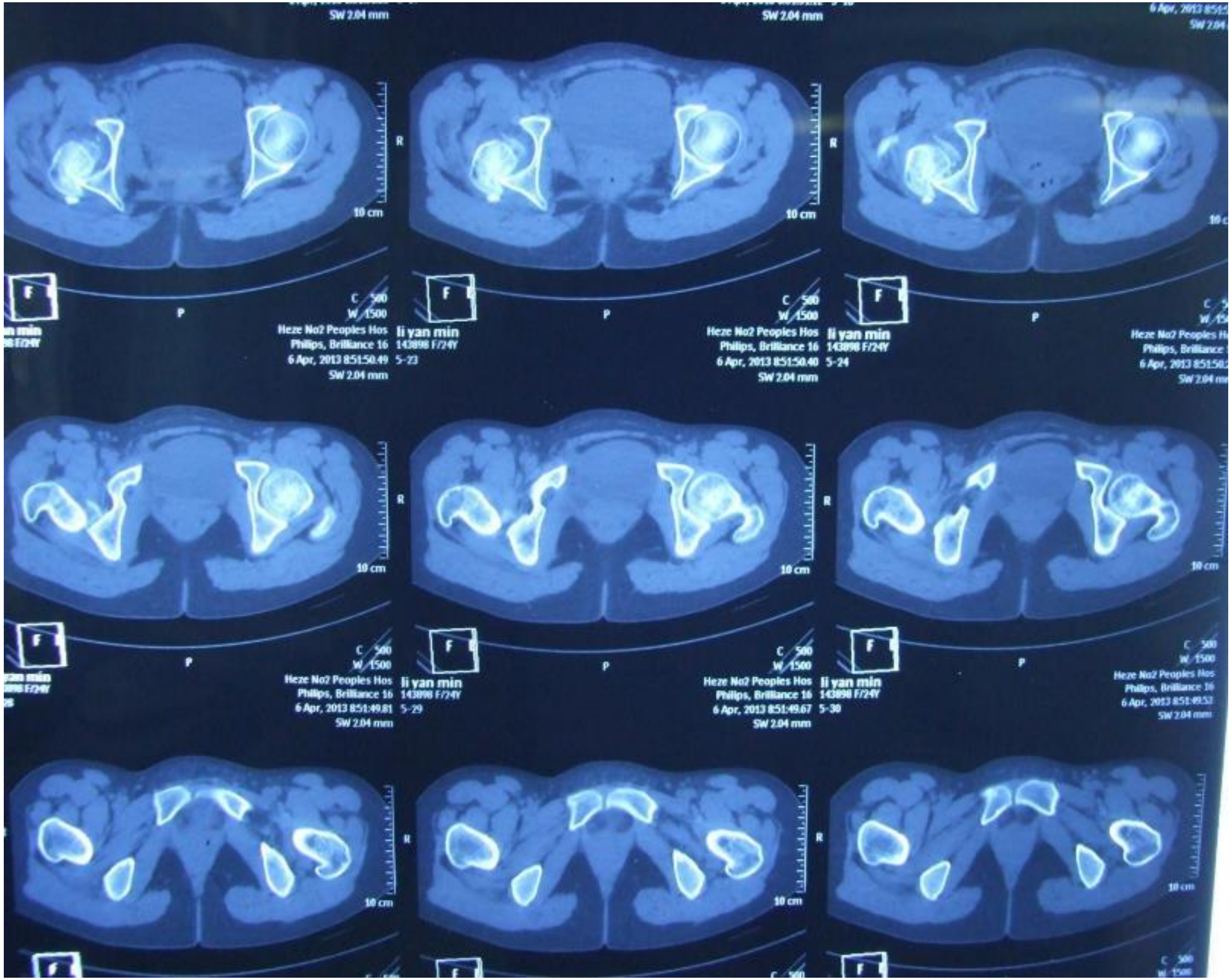

SW 2.04 mm
SW 2.04 mm
R
10 cm
P
C 500
W 1500
Heze No2 Peoples Hos
Philips, Brilliance 16
6 Apr, 2013 8:51:50.49
SW 2.04 mm
li yan min
143898 F/24Y
5-23
R
10 cm
P
C 500
W 1500
Heze No2 Peoples Hos
Philips, Brilliance 16
6 Apr, 2013 8:51:50.40
SW 2.04 mm
li yan min
143898 F/24Y
5-24
R
P
R
10 cm
P
C 500
W 1500
Heze No2 Peoples Hos
Philips, Brilliance 16
6 Apr, 2013 8:51:49.81
SW 2.04 mm
li yan min
143898 F/24Y
5-29
R
10 cm
P
C 500
W 1500
Heze No2 Peoples Hos
Philips, Brilliance 16
6 Apr, 2013 8:51:49.67
SW 2.04 mm
li yan min
143898 F/24Y
5-30
R
10 cm
P
C 500
W 1500
Heze No2 Peoples Hos
Philips, Brilliance 16
6 Apr, 2013 8:51:49.53
SW 2.04 mm
R
10 cm
R
10 cm
10 cm
C 500
W 1500

Intraoperative

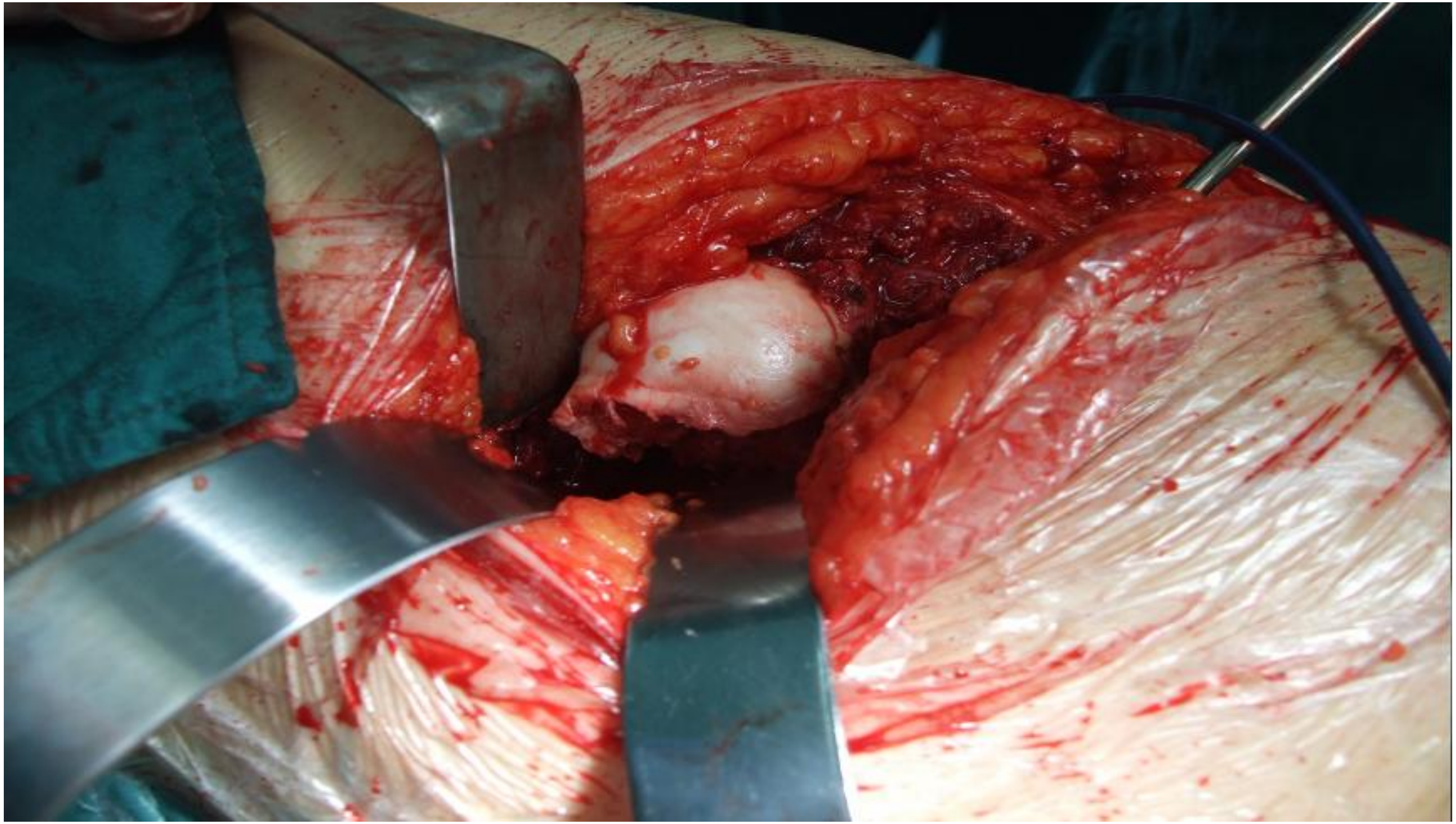

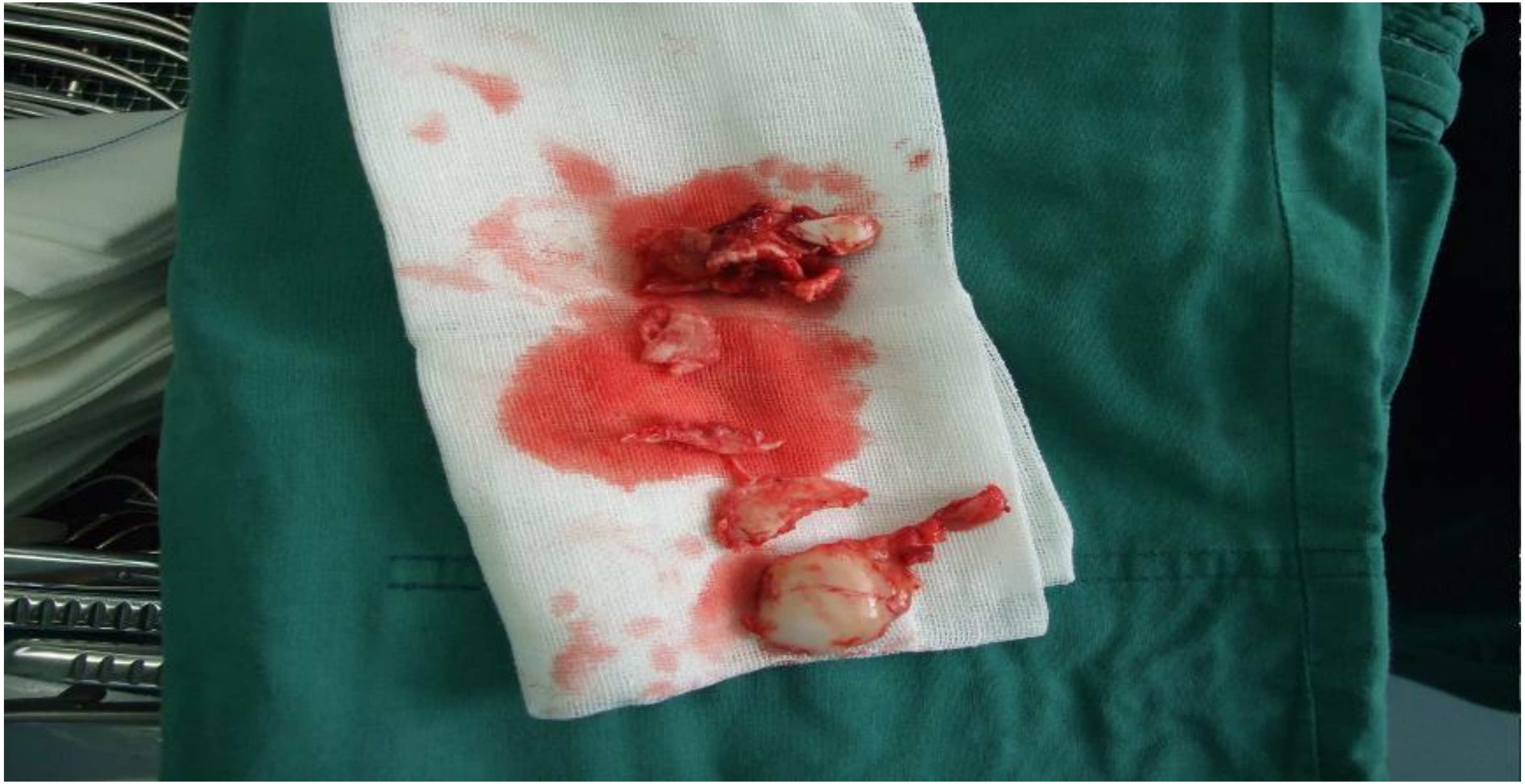

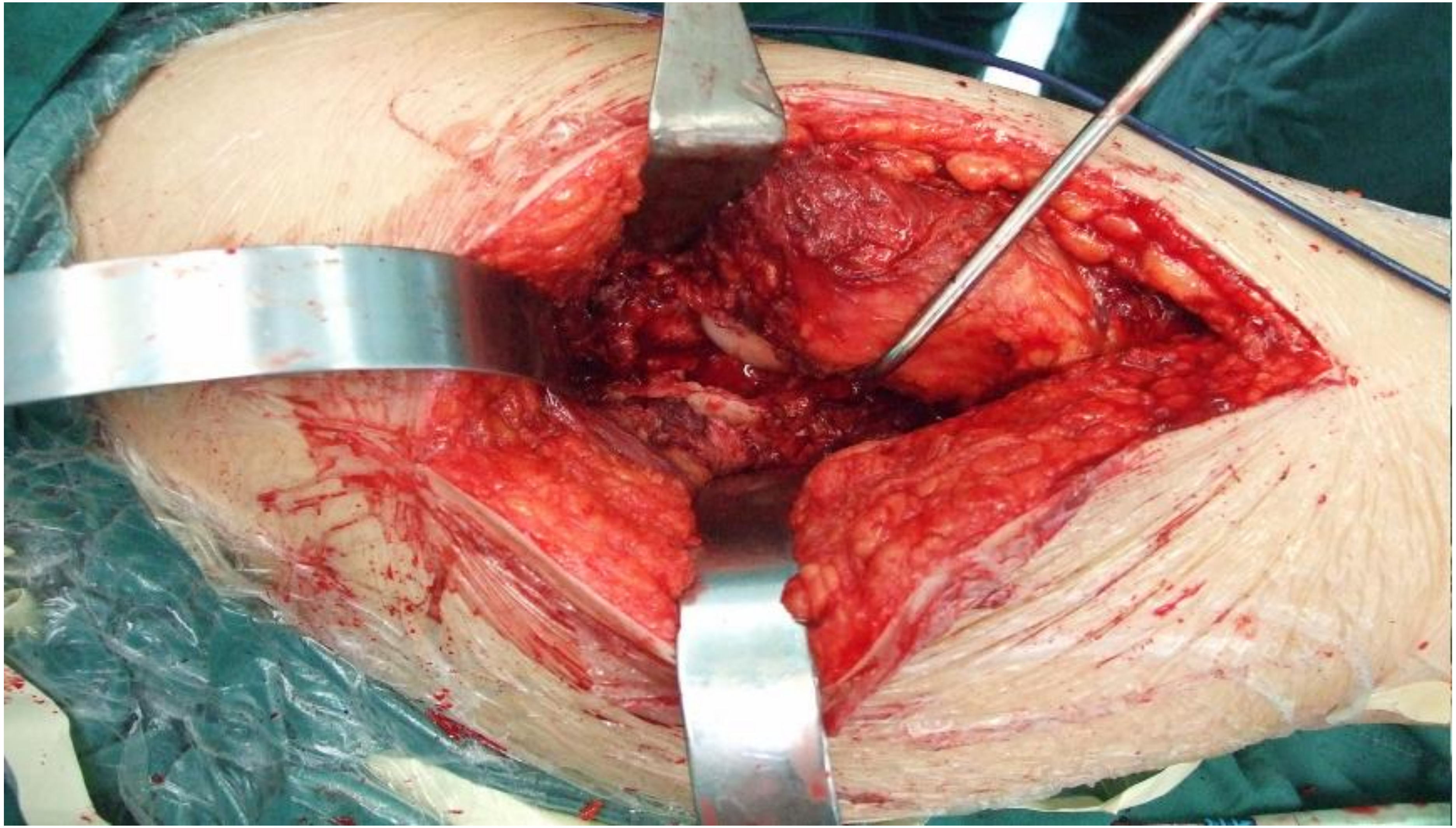

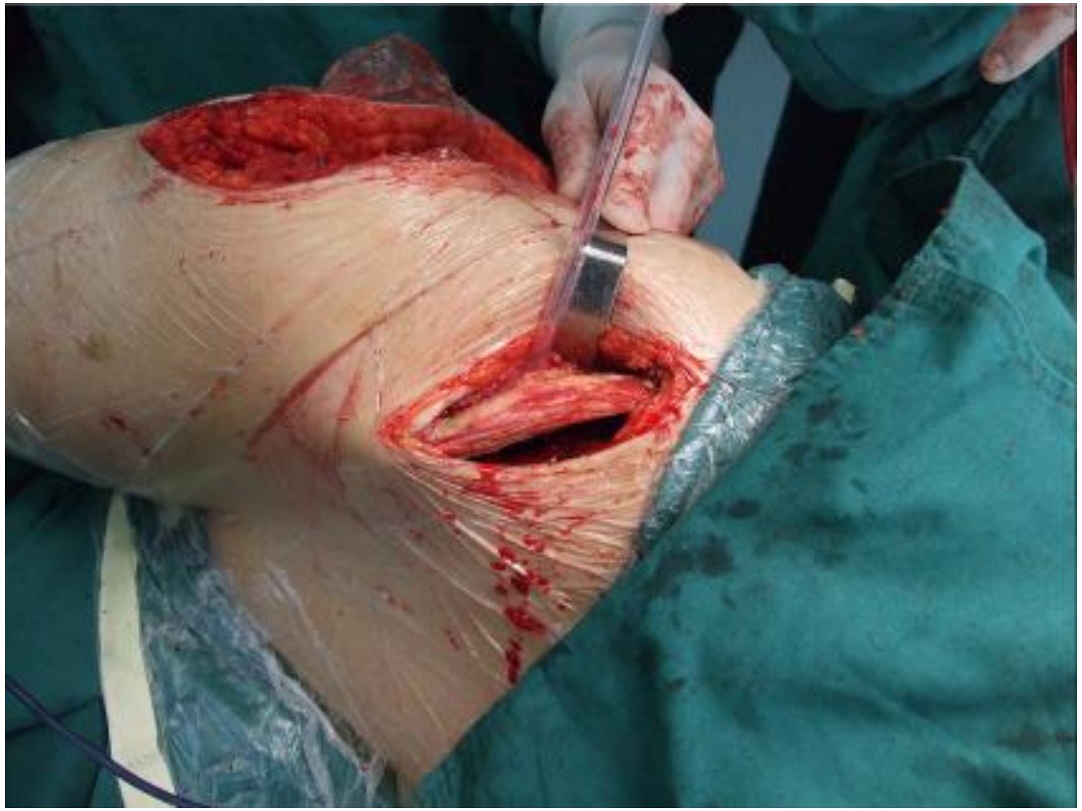
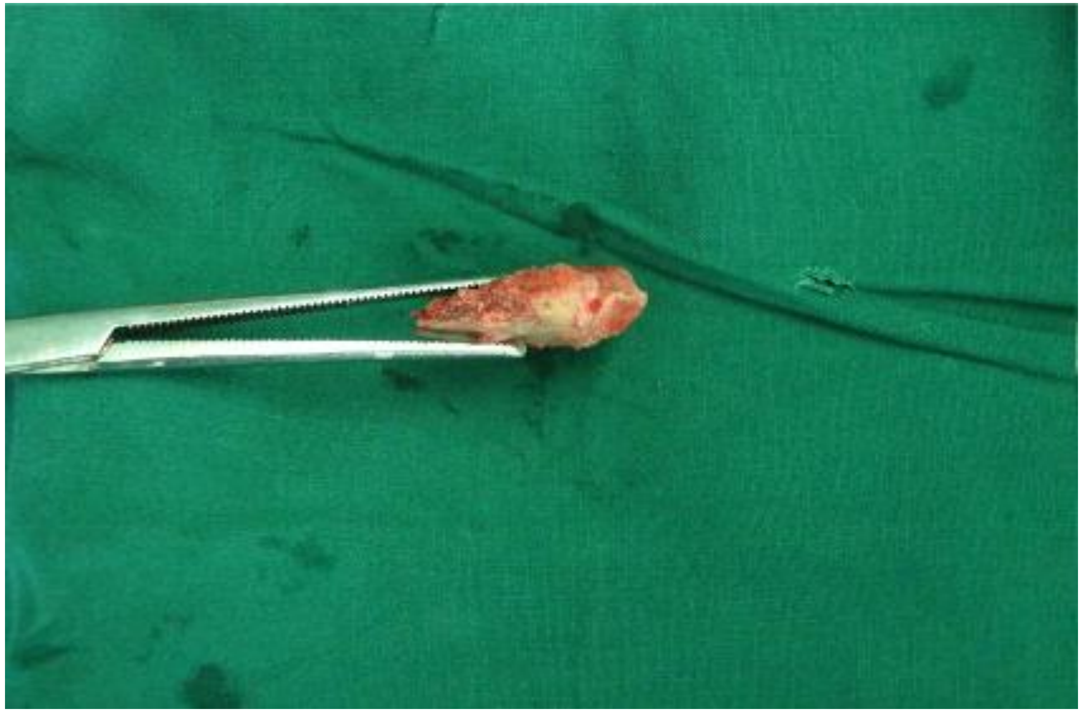
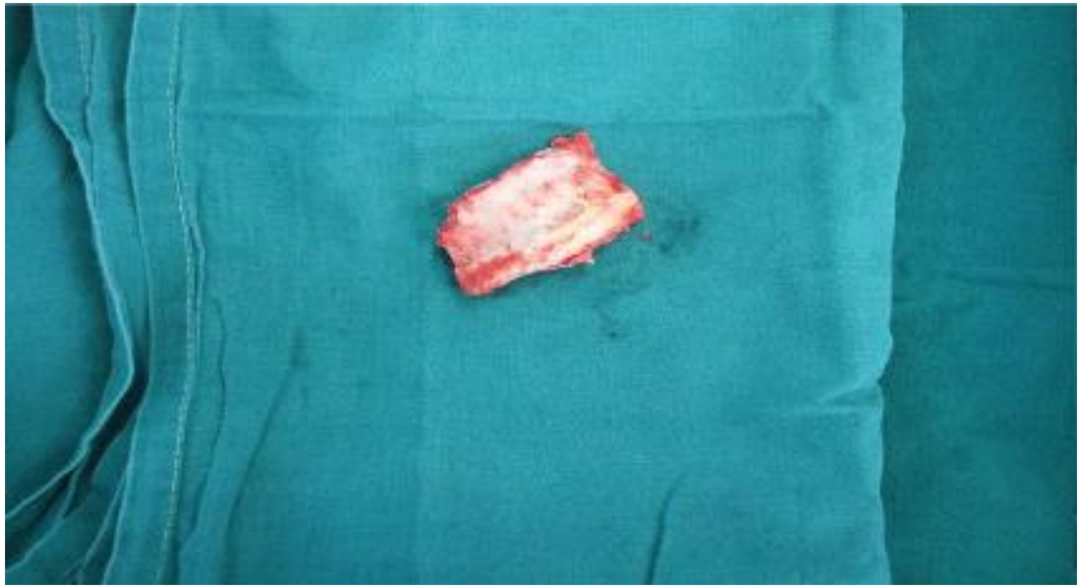

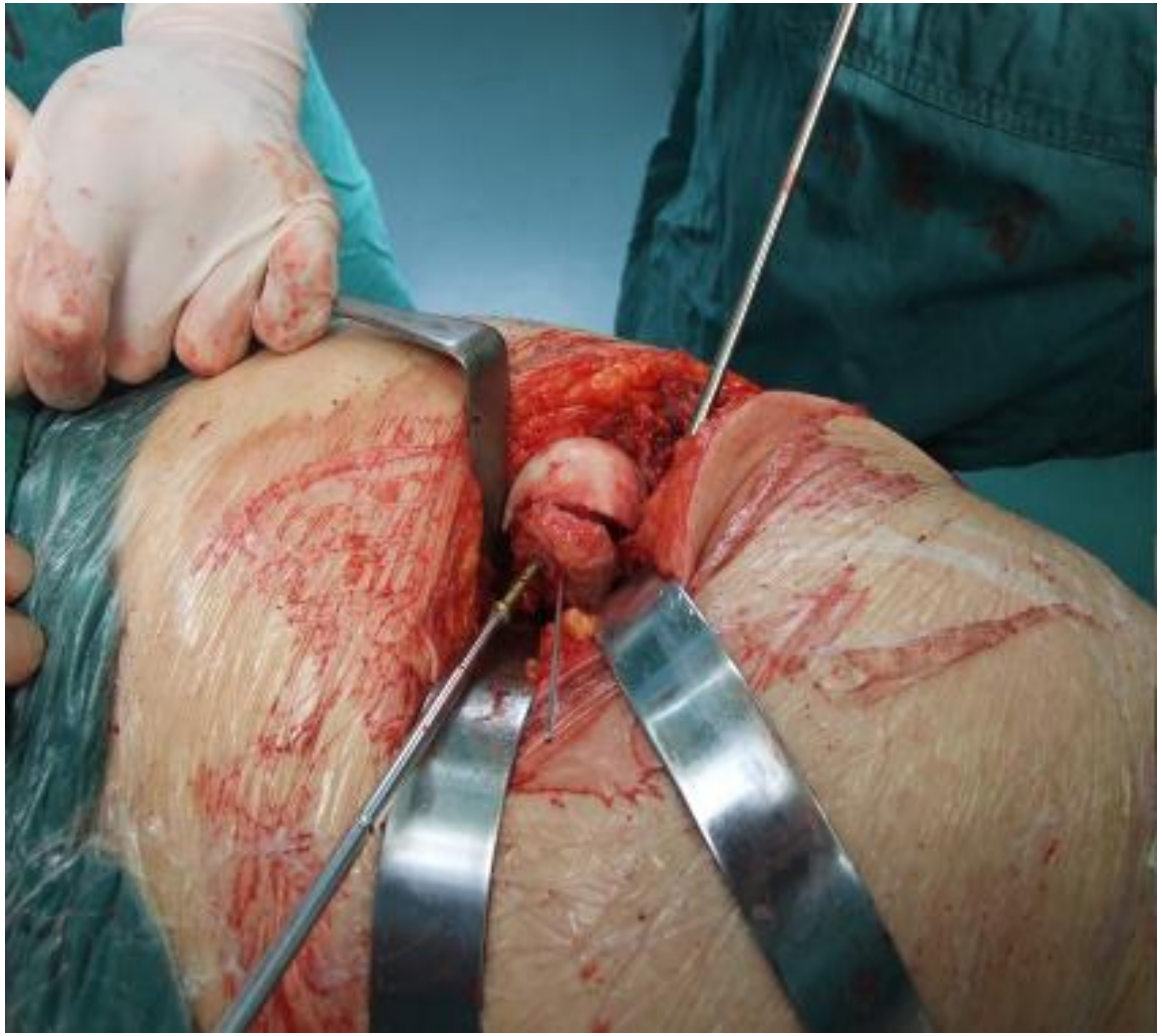
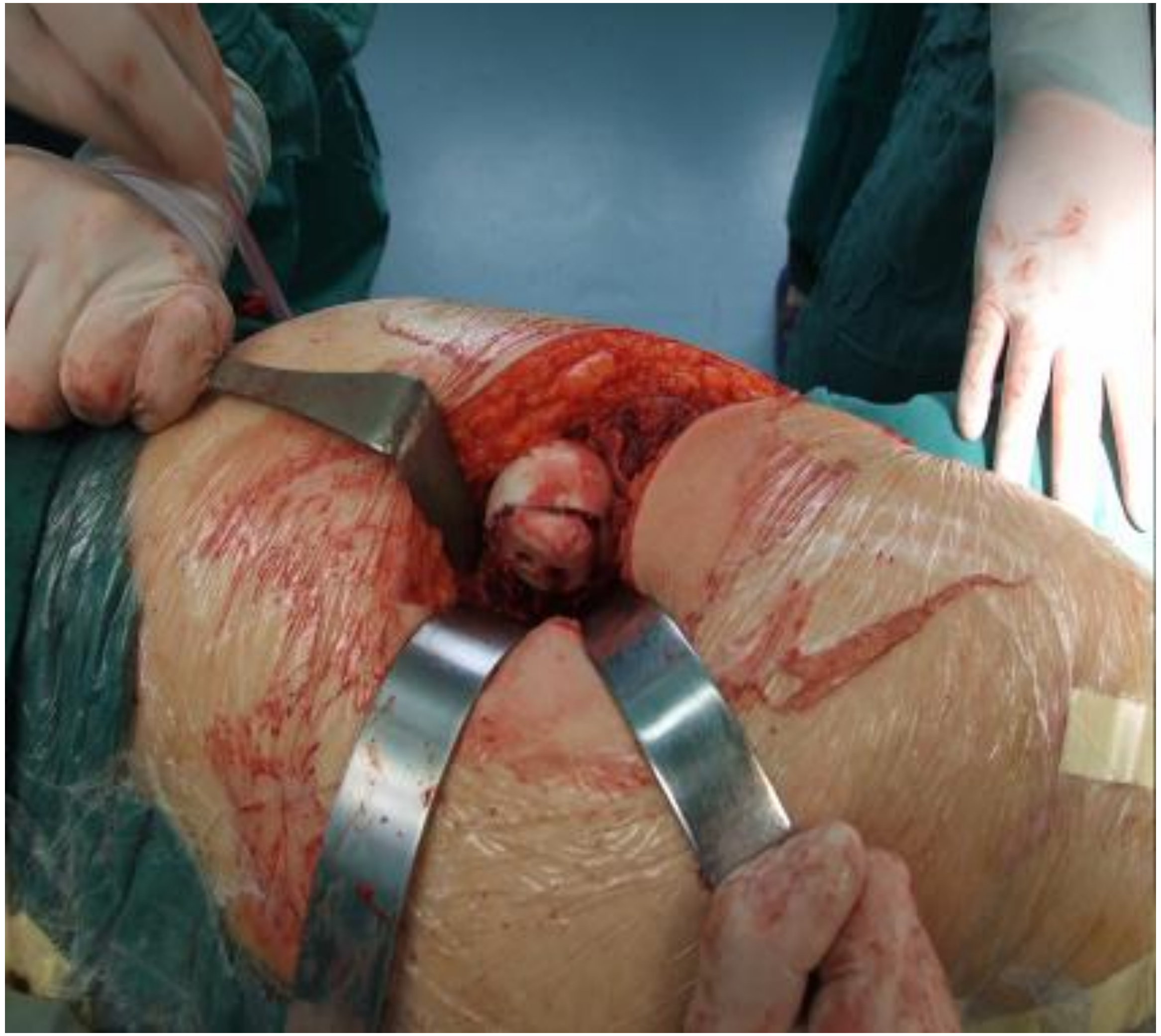

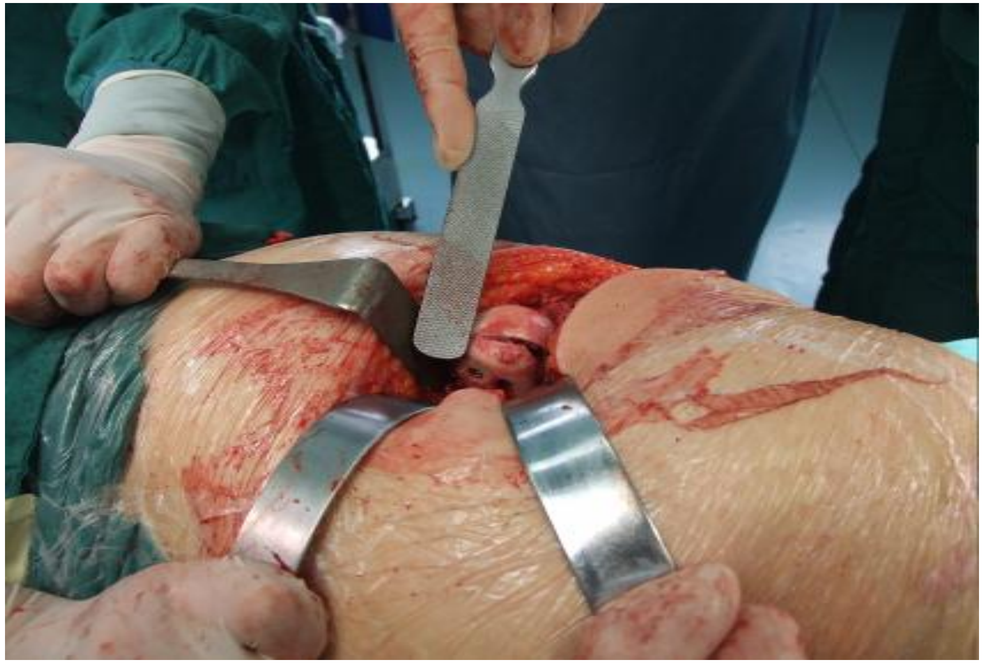
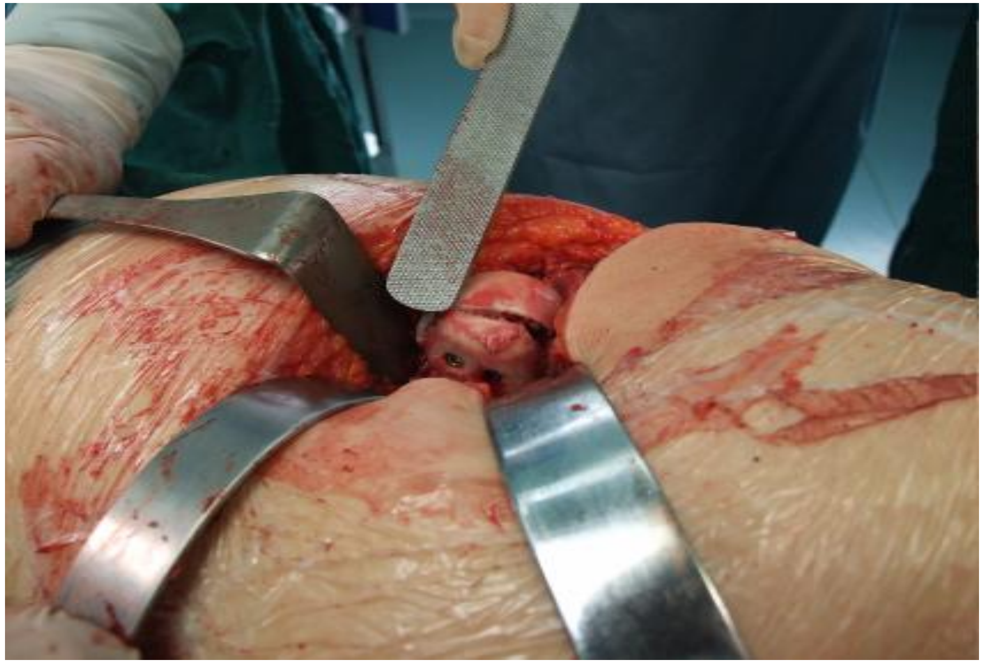
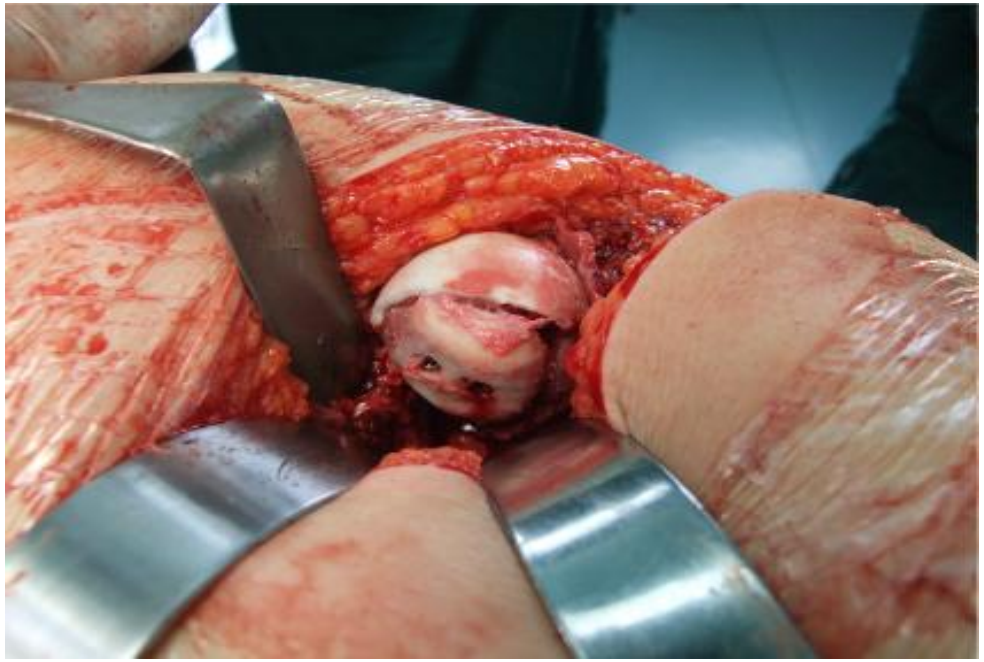
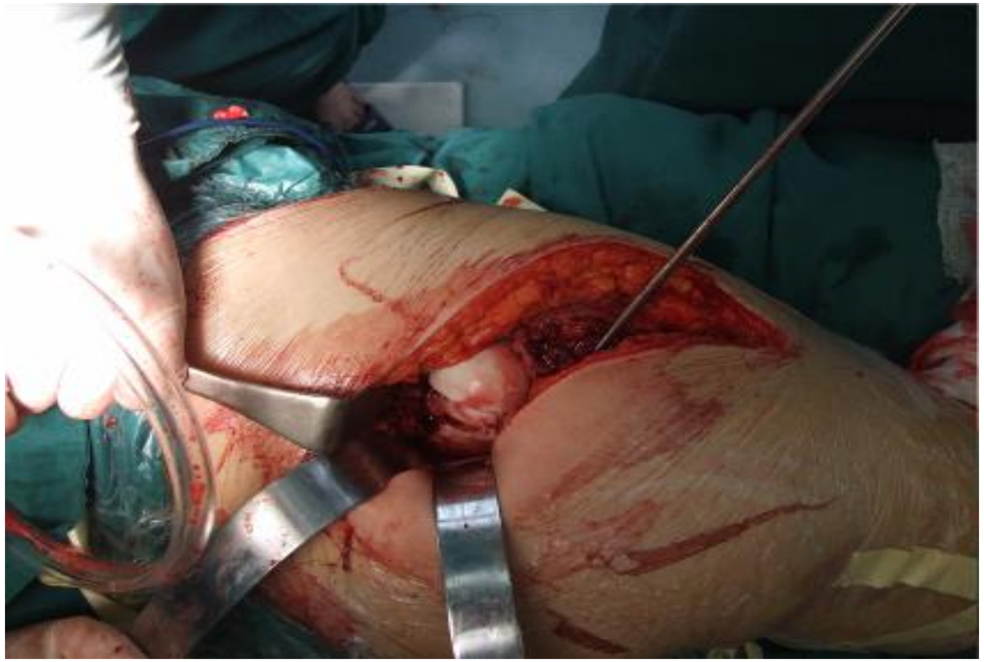

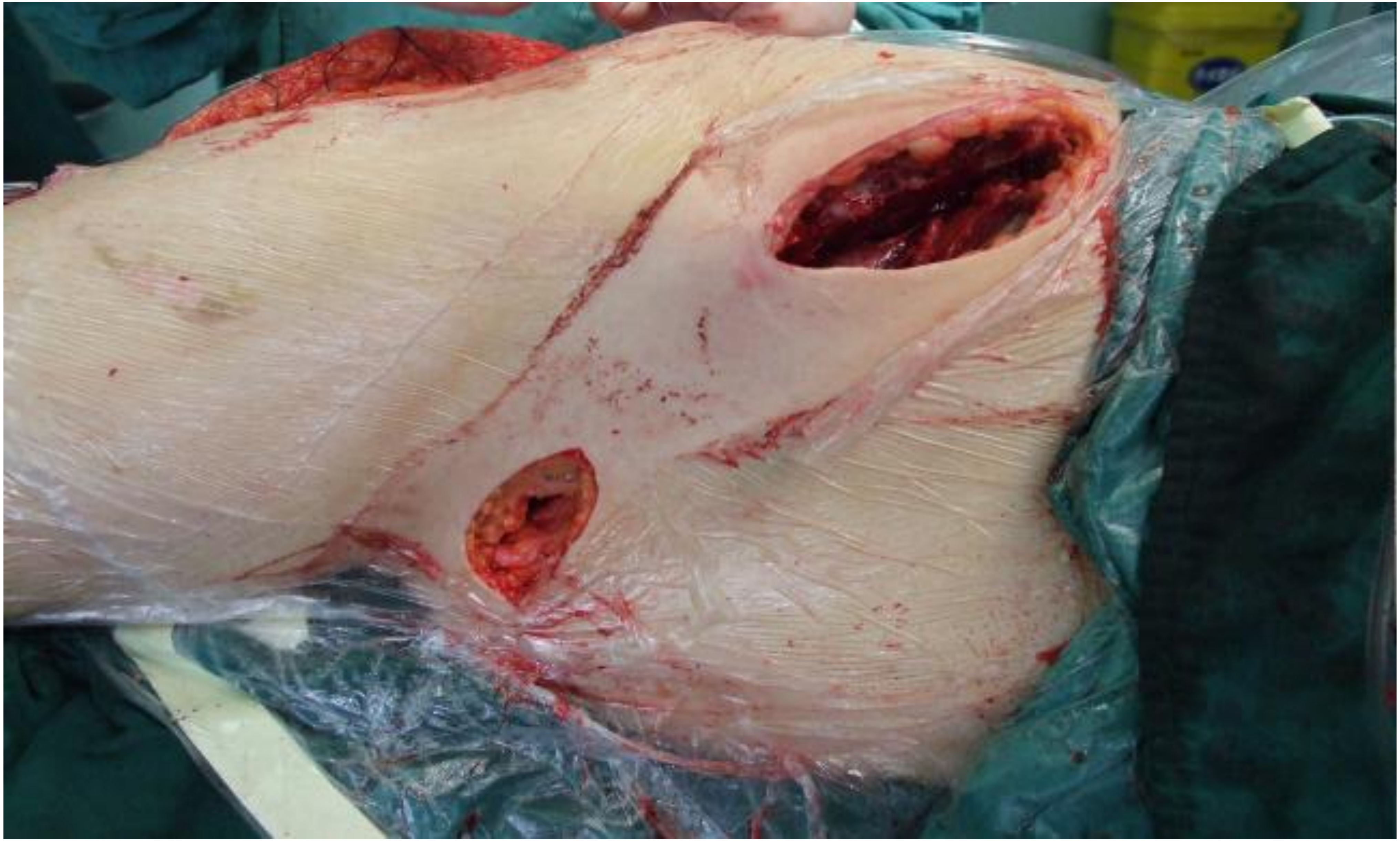

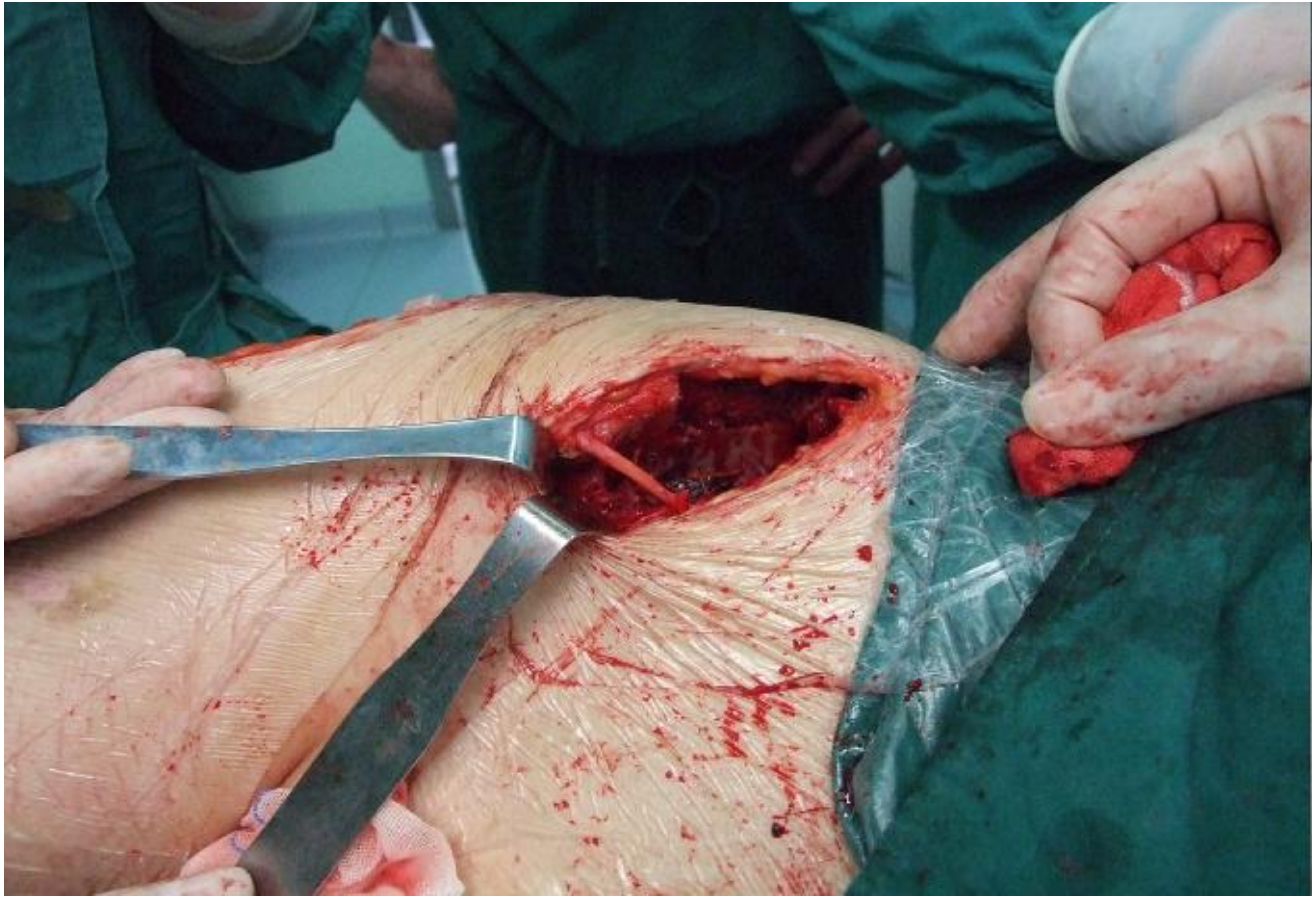

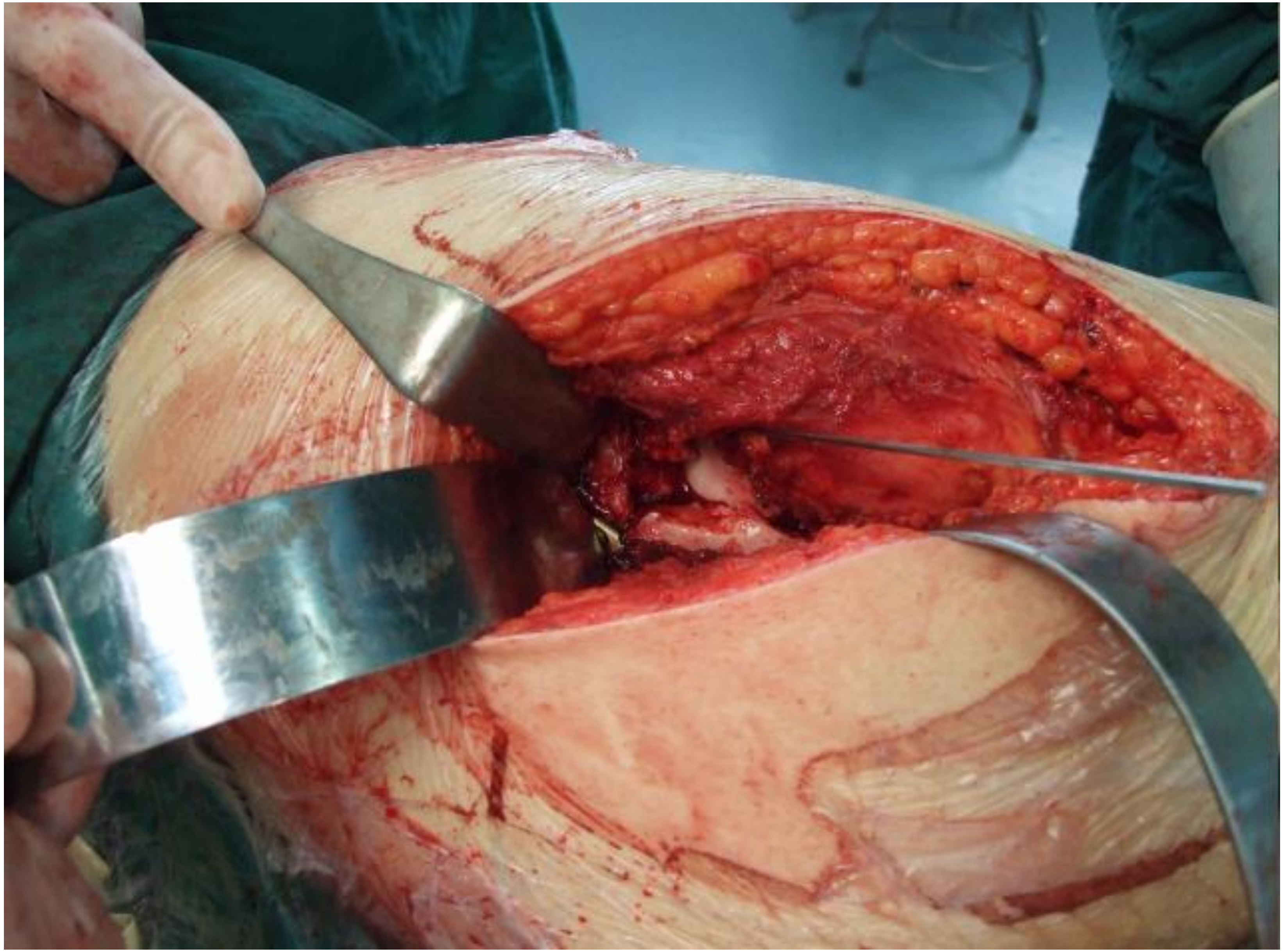

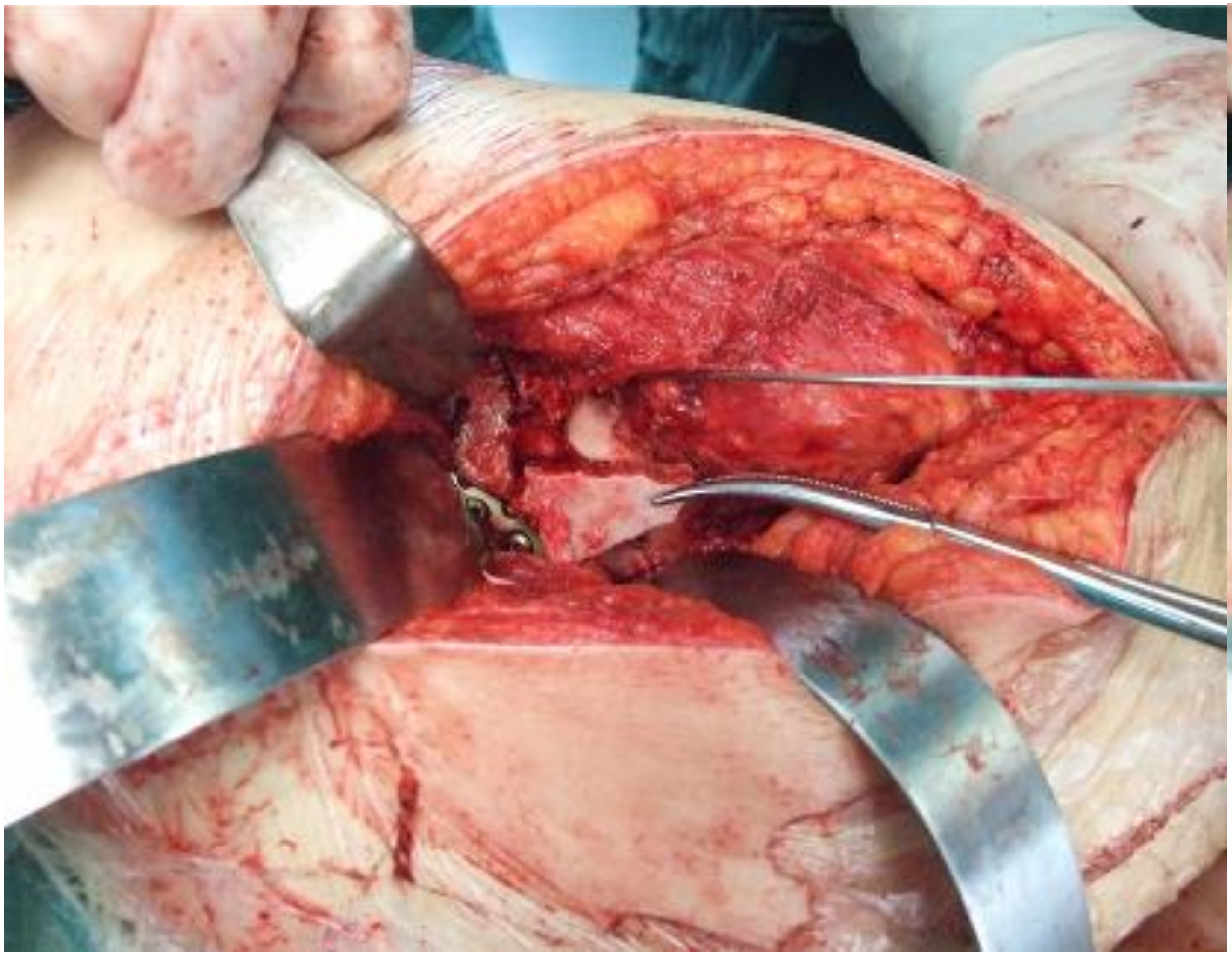
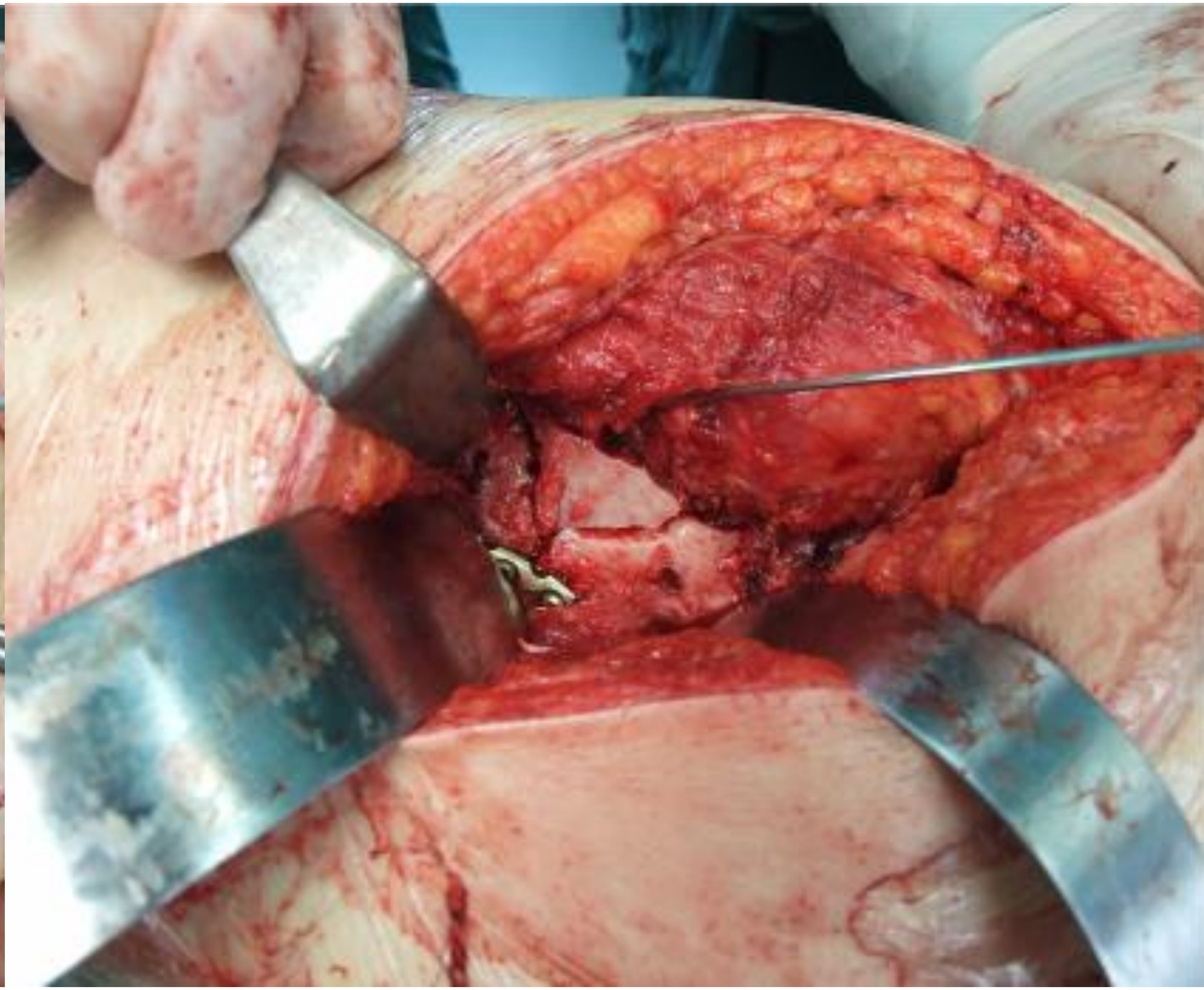

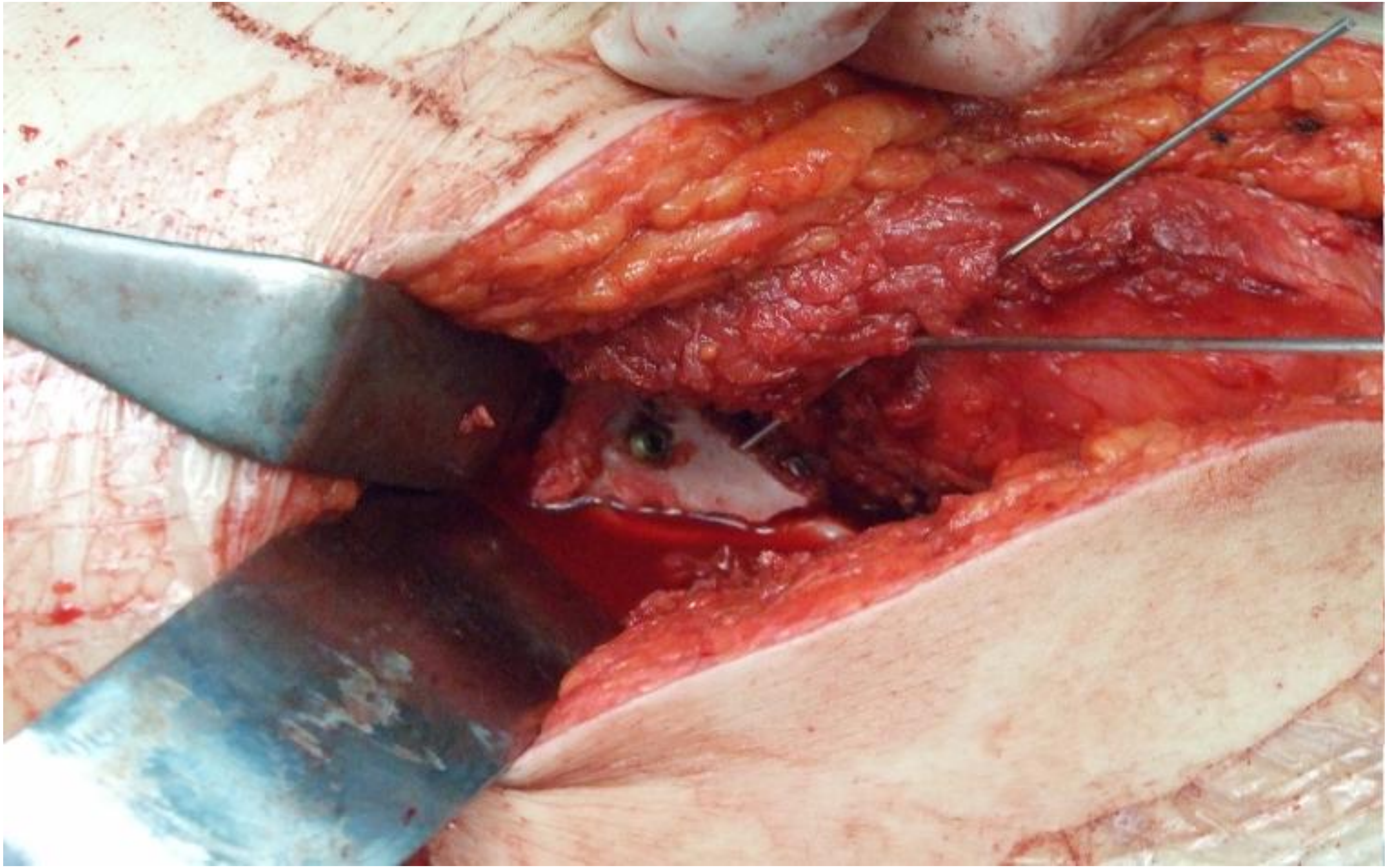

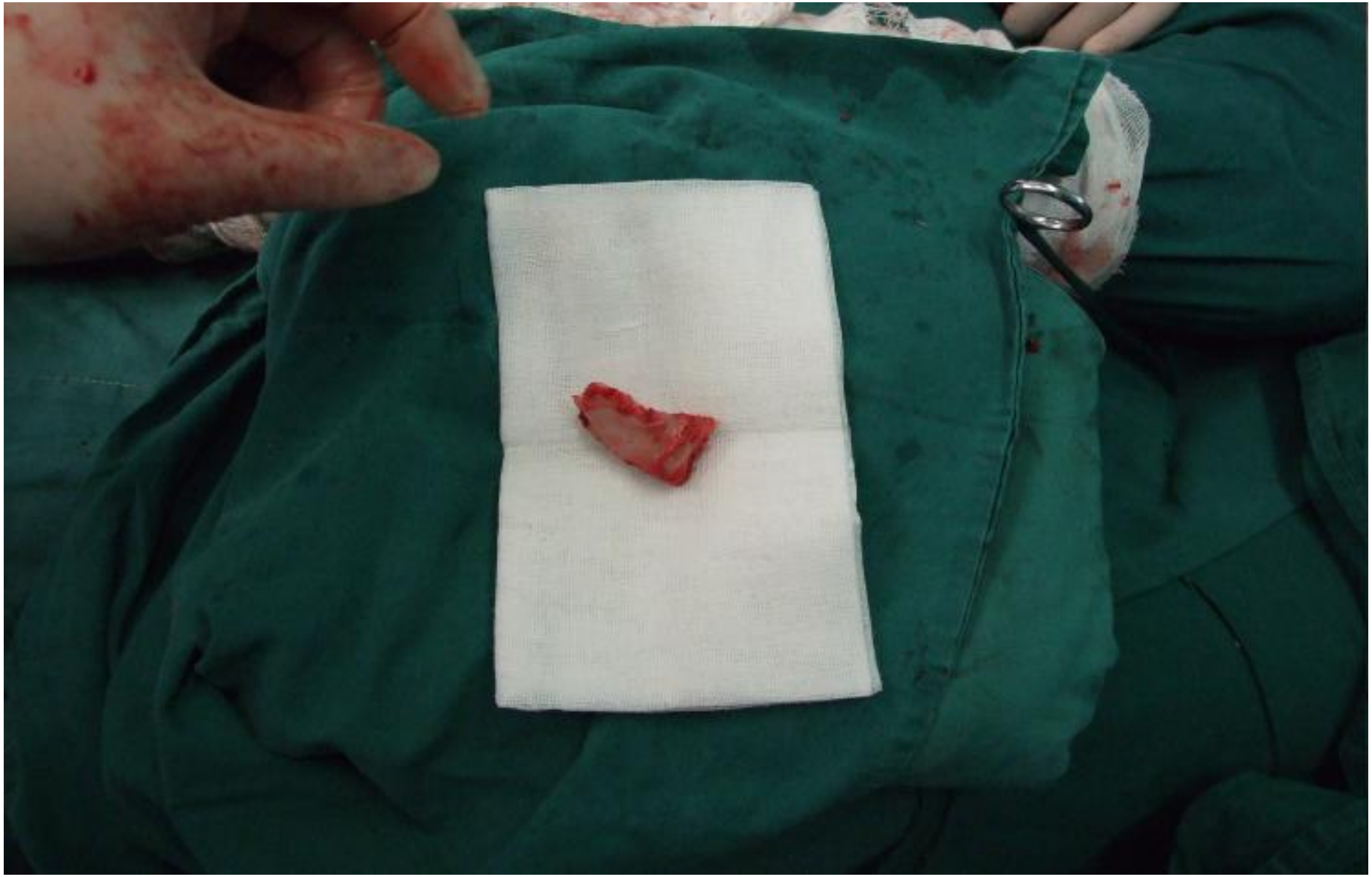

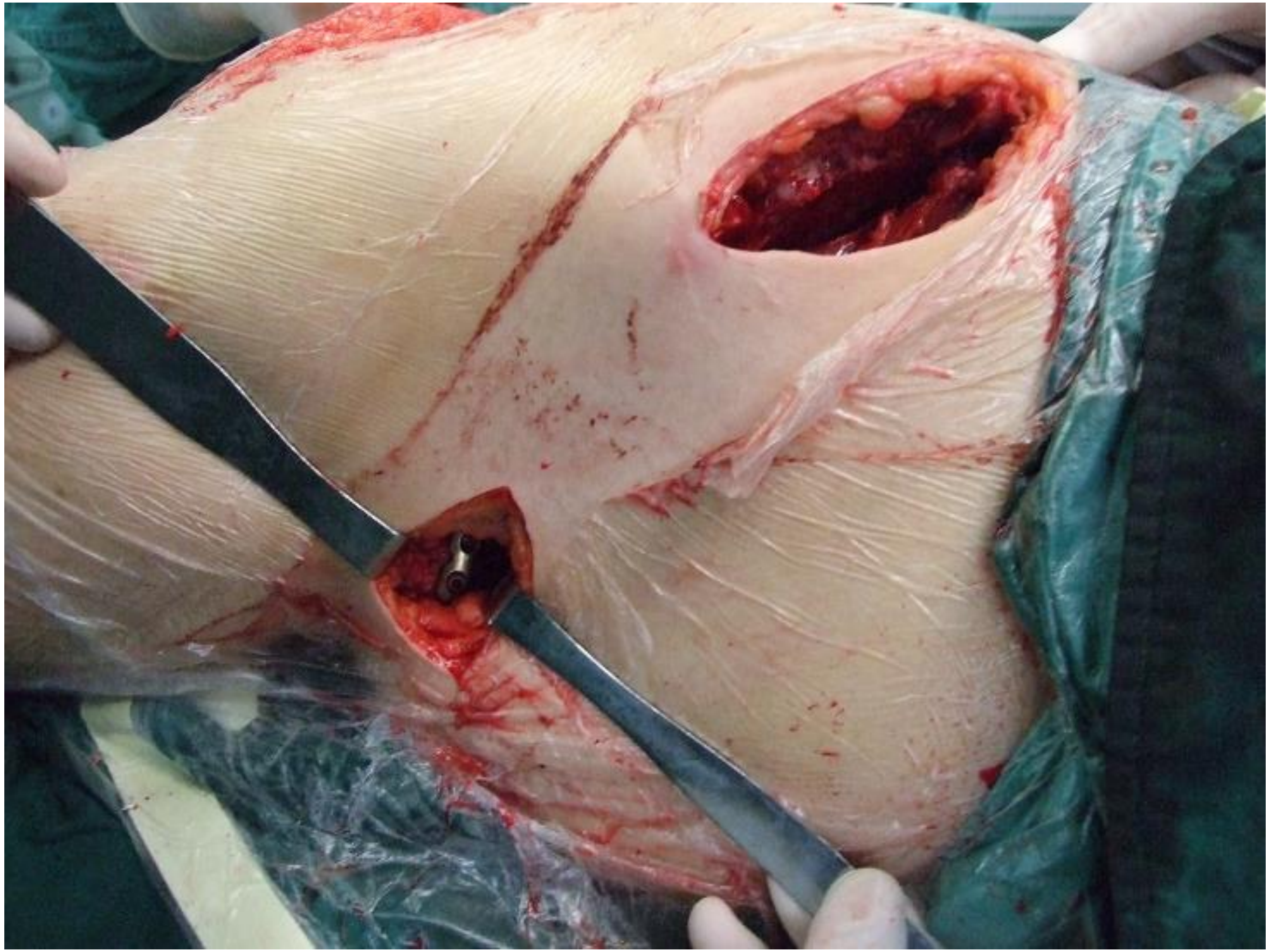

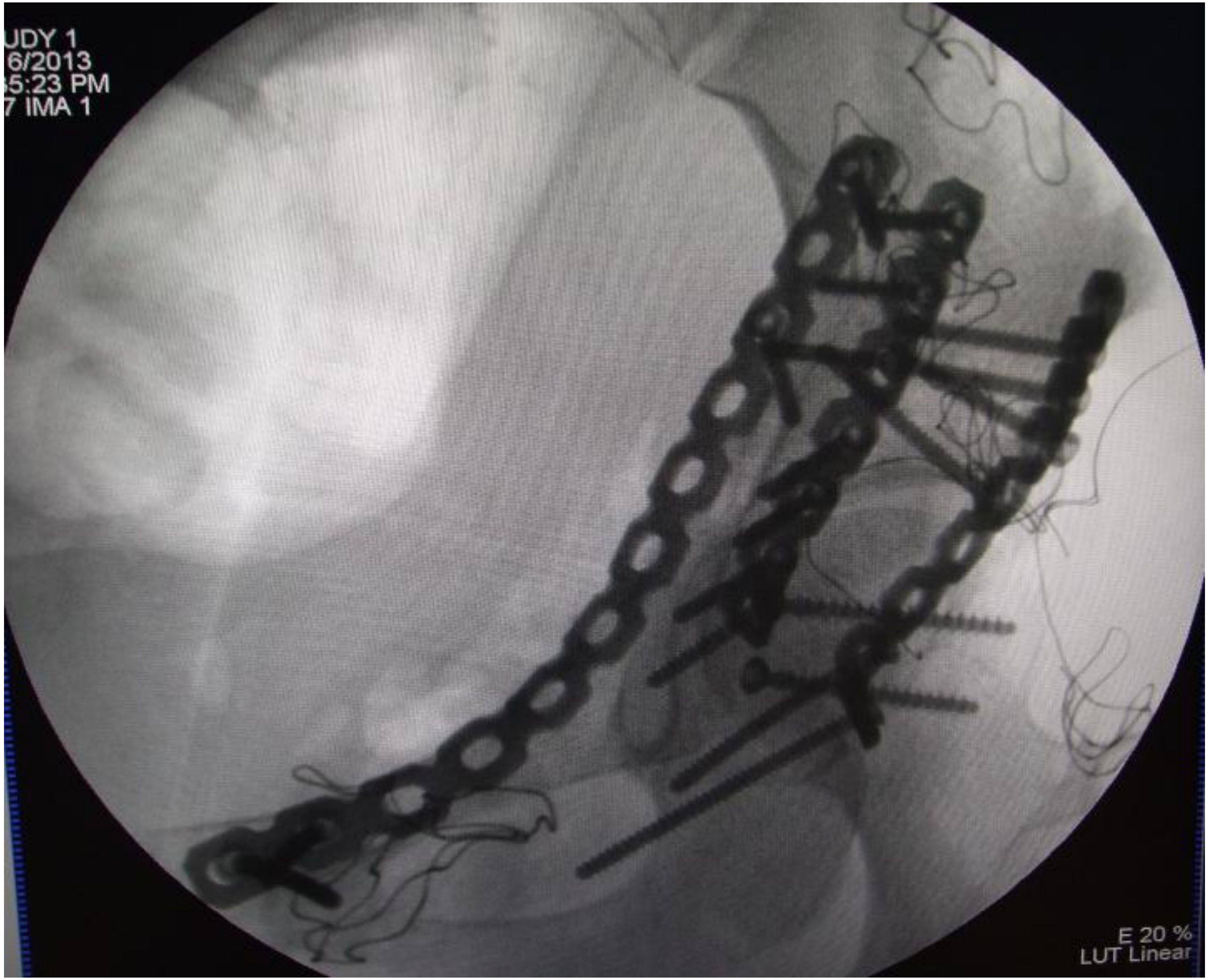
UDY 1
6/2013
35:23 PM
7 IMA 1
E 20 %
LUT Linear

Postoperative

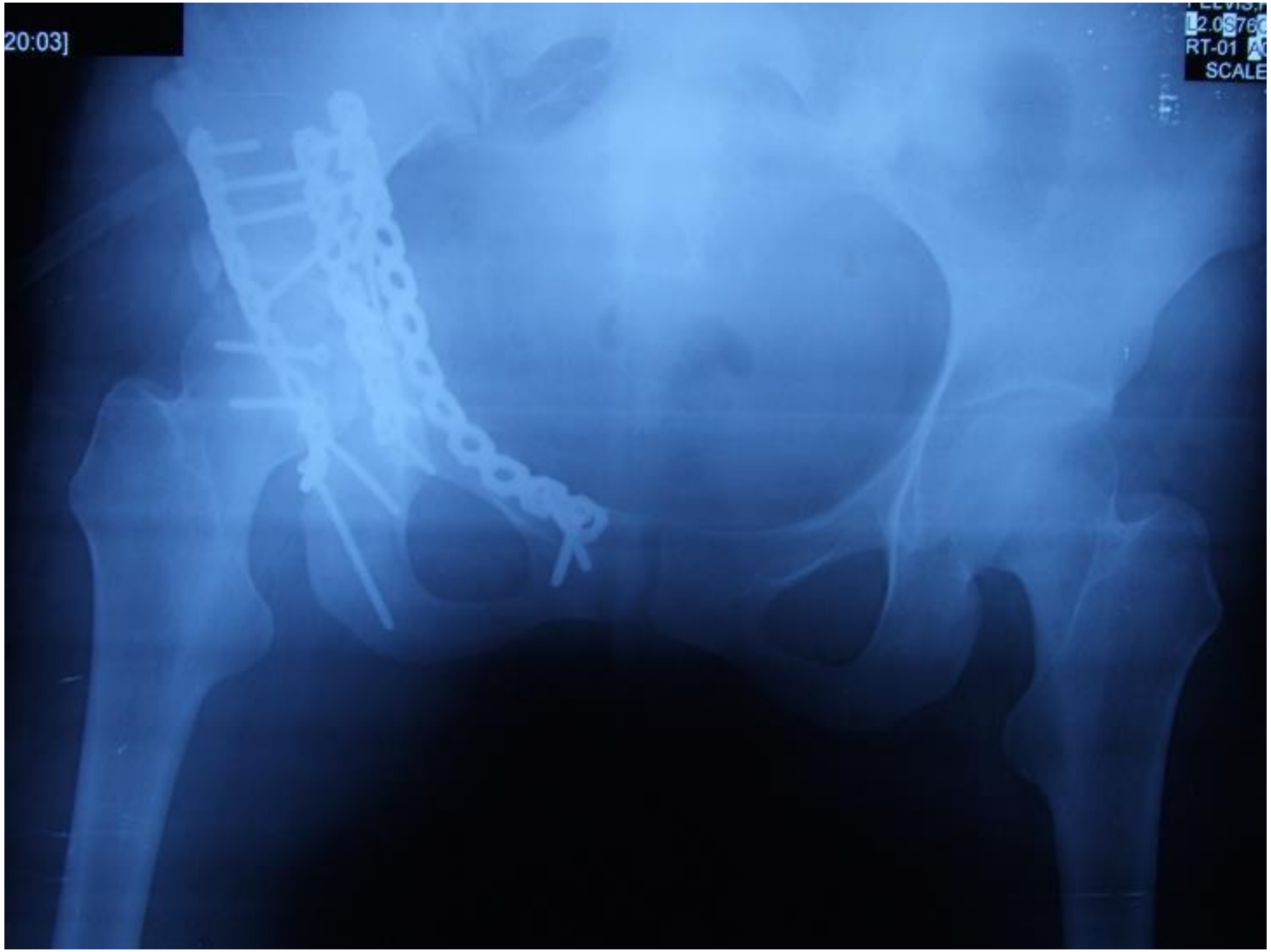

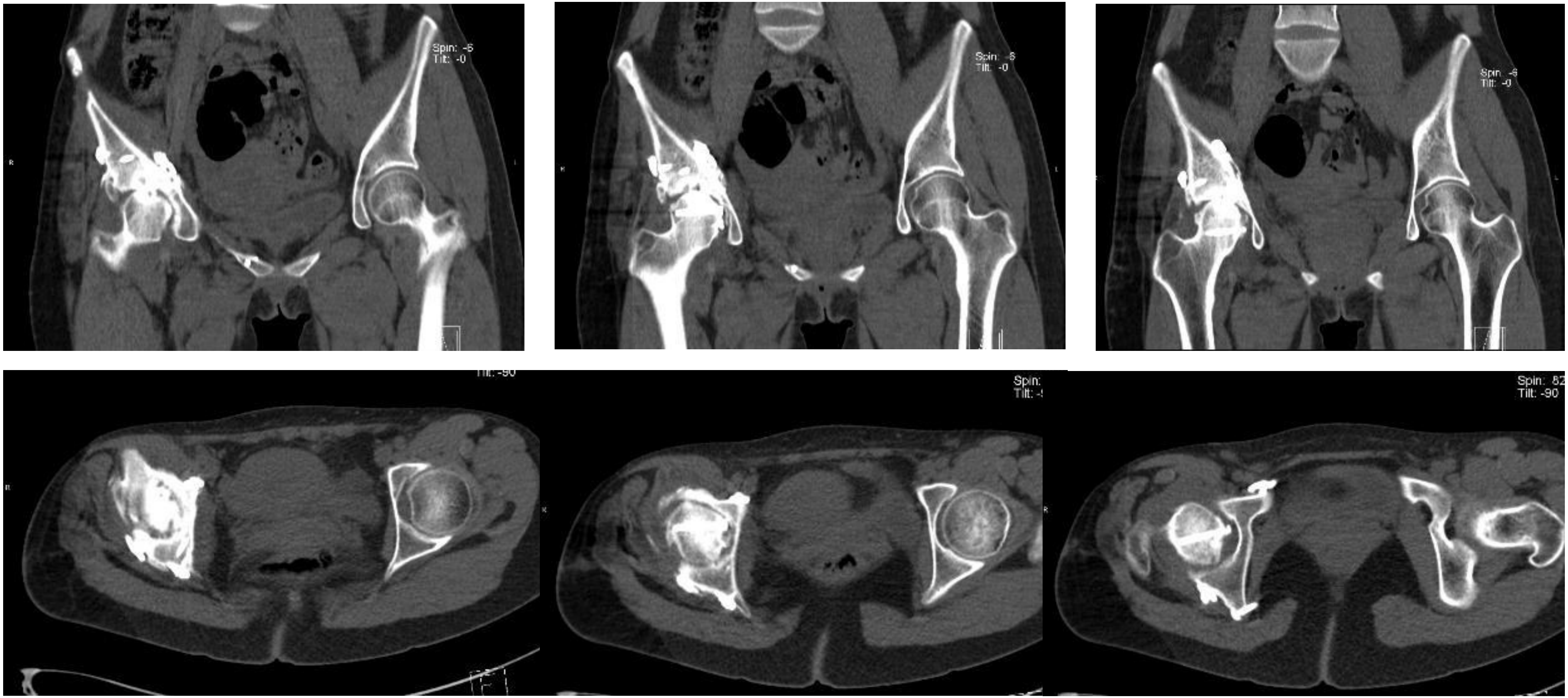
Spin: -6
Tilt: -0
Spin: -6
Tilt: -0
Spin: -6
Tilt: -0
Tilt: -90
Spin:
Spin: 82
Tilt: -90

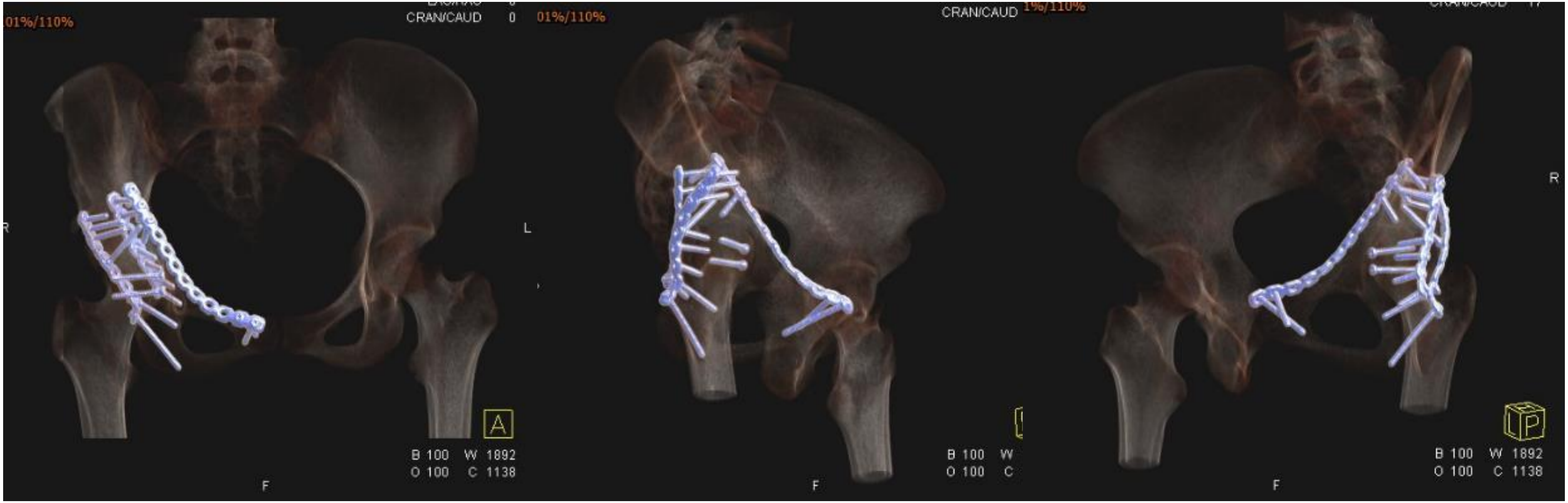
01%/110%
CRAN/CAUD 0
01%/110%
R
L
A
B 100 W 1892
O 100 C 1138
F
CRAN/CAUD
B 100 W
O 100 C
F
R
B 100 W 1892
O 100 C 1138
F

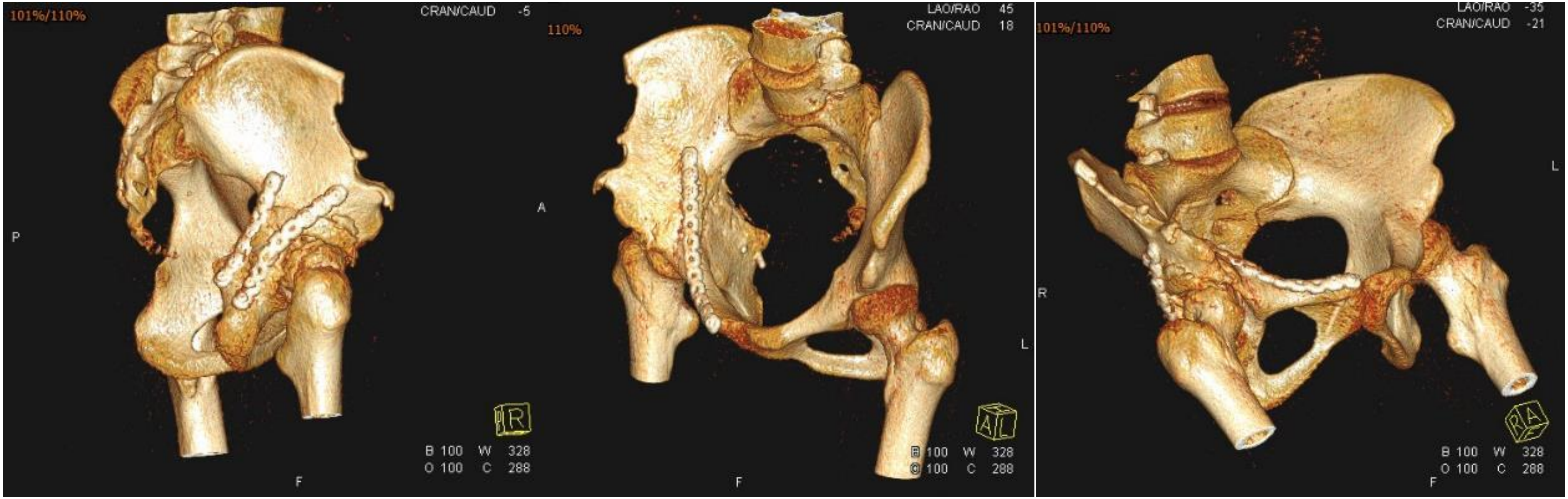
101%/110%
CRAN/CAUD -5
P
A
B 100 W 328
O 100 C 288
F
110%
LAO/RAO 45
CRAN/CAUD 18
L
B 100 W 328
O 100 C 288
F
101%/110%
CRAN/CAUD -21
L
R
B 100 W 328
O 100 C 288
F
