# Supplementary material for: A combination of the K-L and S-P approaches for treating acetabular posterior wall factures accompanied by femoral head fractures with open reduction and internal fixation
Source: BMC Surg. 2022 May 10;22:165. doi: 10.1186/s12893-022-01597-w (PMC9092786; doi:10.1186/s12893-022-01597-w)
Supplement: Supplementary file 2 — Additional file 2. Statistical data. [file 12893_2022_1597_MOESM2_ESM.pdf]

Statistical data (from spss)

Statistical description

	Number of cases	Mean	Standard deviation	Standard error	95% confidence interval of the mean		Minimum value	Maximum value
					Lower limit	Upper limit		
1.00	8	57.3750	4.77905	1.68965	53.3796	61.3704	50.00	65.00
2.00	8	76.1250	3.68152	1.30161	73.0472	79.2028	70.00	80.00
3.00	8	88.2500	3.49489	1.23563	85.3282	91.1718	83.00	93.00
4.00	8	92.1250	2.23207	.78916	90.2589	93.9911	89.00	95.00
Total	32	78.4688	14.18310	2.50724	73.3552	83.5823	50.00	95.00

Variance homogeneity test

		Levin statistics	Degree of freedom 1	Degree of freedom 2	Significance
	Based on mean	1.656	3	28	.199
	Based on median	1.261	3	28	.307
	Based on median and with adjusted degrees of freedom	1.261	3	23.059	.311
	Based on the mean value after clipping	1.676	3	28	.195

ANOVA

	Sum of squares	Degree of freedom	Mean square	F	Significance
Inter-group	5860.844	3	1953.615	145.821	.000
Intra-group	375.125	28	13.397		
Total	6235.969	31			

Multiple comparison

						95% confidence interval	
	(I)	(J)	Mean deviation (I-J)	Standard error	Significance	Lower limit	Upper limit
LSD	1.00	2.00	-18.75000*	1.83012	.000	-22.4988	-15.0012
		3.00	-30.87500*	1.83012	.000	-34.6238	-27.1262
		4.00	-34.75000*	1.83012	.000	-38.4988	-31.0012
	2.00	1.00	18.75000*	1.83012	.000	15.0012	22.4988
		3.00	-12.12500*	1.83012	.000	-15.8738	-8.3762
		4.00	-16.00000*	1.83012	.000	-19.7488	-12.2512
	3.00	1.00	30.87500*	1.83012	.000	27.1262	34.6238
		2.00	12.12500*	1.83012	.000	8.3762	15.8738
		4.00	-3.87500*	1.83012	.043	-7.6238	-.1262
	4.00	1.00	34.75000*	1.83012	.000	31.0012	38.4988
		2.00	16.00000*	1.83012	.000	12.2512	19.7488
		3.00	3.87500*	1.83012	.043	.1262	7.6238

Tamhane	1.00	2.00	-18.75000*	2.13286	.000	-25.3408	-12.1592
		3.00	-30.87500*	2.09325	.000	-37.3713	-24.3787
		4.00	-34.75000*	1.86485	.000	-40.8496	-28.6504
	2.00	1.00	18.75000*	2.13286	.000	12.1592	25.3408
		3.00	-12.12500*	1.79471	.000	-17.6157	-6.6343
		4.00	-16.00000*	1.52216	.000	-20.8183	-11.1817
	3.00	1.00	30.87500*	2.09325	.000	24.3787	37.3713
		2.00	12.12500*	1.79471	.000	6.6343	17.6157
		4.00	-3.87500	1.46614	.123	-8.4881	.7381
	4.00	1.00	34.75000*	1.86485	.000	28.6504	40.8496
		2.00	16.00000*	1.52216	.000	11.1817	20.8183
		3.00	3.87500	1.46614	.123	-.7381	8.4881
Dunnett T3	1.00	2.00	-18.75000*	2.13286	.000	-25.2557	-12.2443
		3.00	-30.87500*	2.09325	.000	-37.2841	-24.4659
		4.00	-34.75000*	1.86485	.000	-40.7264	-28.7736
	2.00	1.00	18.75000*	2.13286	.000	12.2443	25.2557
		3.00	-12.12500*	1.79471	.000	-17.5511	-6.6989
		4.00	-16.00000*	1.52216	.000	-20.7420	-11.2580
	3.00	1.00	30.87500*	2.09325	.000	24.4659	37.2841
		2.00	12.12500*	1.79471	.000	6.6989	17.5511
		4.00	-3.87500	1.46614	.111	-8.4185	.6685
	4.00	1.00	34.75000*	1.86485	.000	28.7736	40.7264
		2.00	16.00000*	1.52216	.000	11.2580	20.7420
		3.00	3.87500	1.46614	.111	-.6685	8.4185

*. The significance level of the mean difference is 0.05。
