# Supplementary material for: A combination of the K-L and S-P approaches for treating acetabular posterior wall factures accompanied by femoral head fractures with open reduction and internal fixation
Source: BMC Surg. 2022 May 10;22:165. doi: 10.1186/s12893-022-01597-w (PMC9092786; doi:10.1186/s12893-022-01597-w)
Supplement: Supplementary file 3 — Additional file 3: Table data. [file 12893_2022_1597_MOESM3_ESM.pdf]

Table 1 (raw data) Main characteristics of 8 patients included studies.

SN	Name	Gender	COI	CWSNI	IBT	Age (Y)	PT(D)	IB (ML)	OT (MIN)
1	-LIU	M	TAI	N	RBC 4U / Plasma 200 ML	42	5	300	240
2	-WANG	M	FI	Y	RBC 2U / Plasma 200 ML	46	4	200	140
3	-CHEN	M	FI	Y	RBC 4U / Plasma 200 ML	52	5	300	130
4	-DAI	M	FI	N	RBC 4U / Plasma 200 ML	52	4	300	120
5	-YU	M	FI	N	RBC 2U / Plasma 200 ML	53	4	200	150
6	-LIANG	M	TAI	N	RBC 2U / Plasma 200 ML	32	6	200	90
7	-ZHANG	M	FI	Y	RBC 2U / Plasma 200 ML	30	7	100	160
8	-LI	M	FI	N	RBC 2U / Plasma 200 ML	24	4	200	120
**DS**						**41.38 ± 11.35**	**4.875 ± 1.126**	**225 ± 70.71**	**143.8 ± 44.38**

SN = serial number, M = male, COI = cause of injury, TAI = traffic accident injury, FI = falling injury, CWSNI = combined with sciatic nerve injury, Y = yes, N = no, IBT = intraoperative blood transfusion, RBC = red blood cell, U = unit, ML = milliliter, Y = Year, PT = preoperative time, D = day, IB = intraoperative bleeding, OT = operation time, MIN = minute, DS = descriptive statistics.

Table 1 Demographic data of eight patients with Pipkin type IV fractures undergoing the K-L approach combined with the S-P approach for ORIF

Category (n=8)	Mean ± SD/ n(%)
Age	41.38 ± 11.35
Preoperative time (day)	4.875 ± 1.126
Intraoperative bleeding (ml)	225.0 ± 70.71
Operation time (min)	143.8 ± 44.38
Intraoperative bleeding	
Red blood cell (U)	2.75 ± 1.035
Plasma (ml)	200.0 ± 0.00
Sex	
Male	8(100.0%)
Female	0(0.0%)
Cause of injury	
Traffic accident injury	2(25.0%)
Falling injury	6(75.0%)
Combined with sciatic nerve injury	
Yes	3(37.5%)
No	5(62.5%)

K-L Kocher-Langenbeck approach

S-P Smith-Petersen approach

ORIF open reduction and internal fixation

Table 2 (raw data) Harris scores of 8 patients postoperative.

SN	Name	Postoperative months and scores (Points)			
		3M	6M	12M	36M
1	-LIU	56	75	85	90
2	-WANG	53	80	85	91
3	-CHEN	50	80	89	93
4	-DAI	65	70	83	89
5	-YU	60	75	90	95
6	-LIANG	60	80	91	91
7	-ZHANG	60	73	93	95
8	-LI	55	76	90	93
**DS**		**57.38 ± 4.779**	**76.13 ± 3.682**	**88.25 ± 3.495**	**92.13 ± 2.232**

SN = serial number, M = months, DS = descriptive statistics.

Table 2 Harris scores of eight patients postoperatively

Category (n=8)	Mean ± SD
3 months	57.38 ± 4.779
6 months	76.13 ± 3.682
12 months	88.25 ± 3.495
36 months	92.13 ± 2.232

Table 3 Imaging evaluation of eight patients up to the last follow-up

Category (n=8)	n(%)
Matta standard	
Excellent (A)	6(75.0%)
Good (B)	2(25.0%)
Joint space stenosis	
Normal	6(75.0%)
Mild stenosis	1(12.5.0%)
Obvious stenosis	1(12.5.0%)
Brooker criteria	
Heterotopic ossification	0
Without heterotopic ossification	8(100.0%)
Ficat-Arlet staging	
Femoral head necrosis	0
Without necrosis of femoral head	8(100.0%)
